# Supplementary figures and images for: Unveiling the domain-specific and RAS isoform-specific details of BRAF kinase regulation
Source: eLife. 2023 Dec 27;12:RP88836. doi: 10.7554/eLife.88836 (PMC10752582; doi:10.7554/eLife.88836)

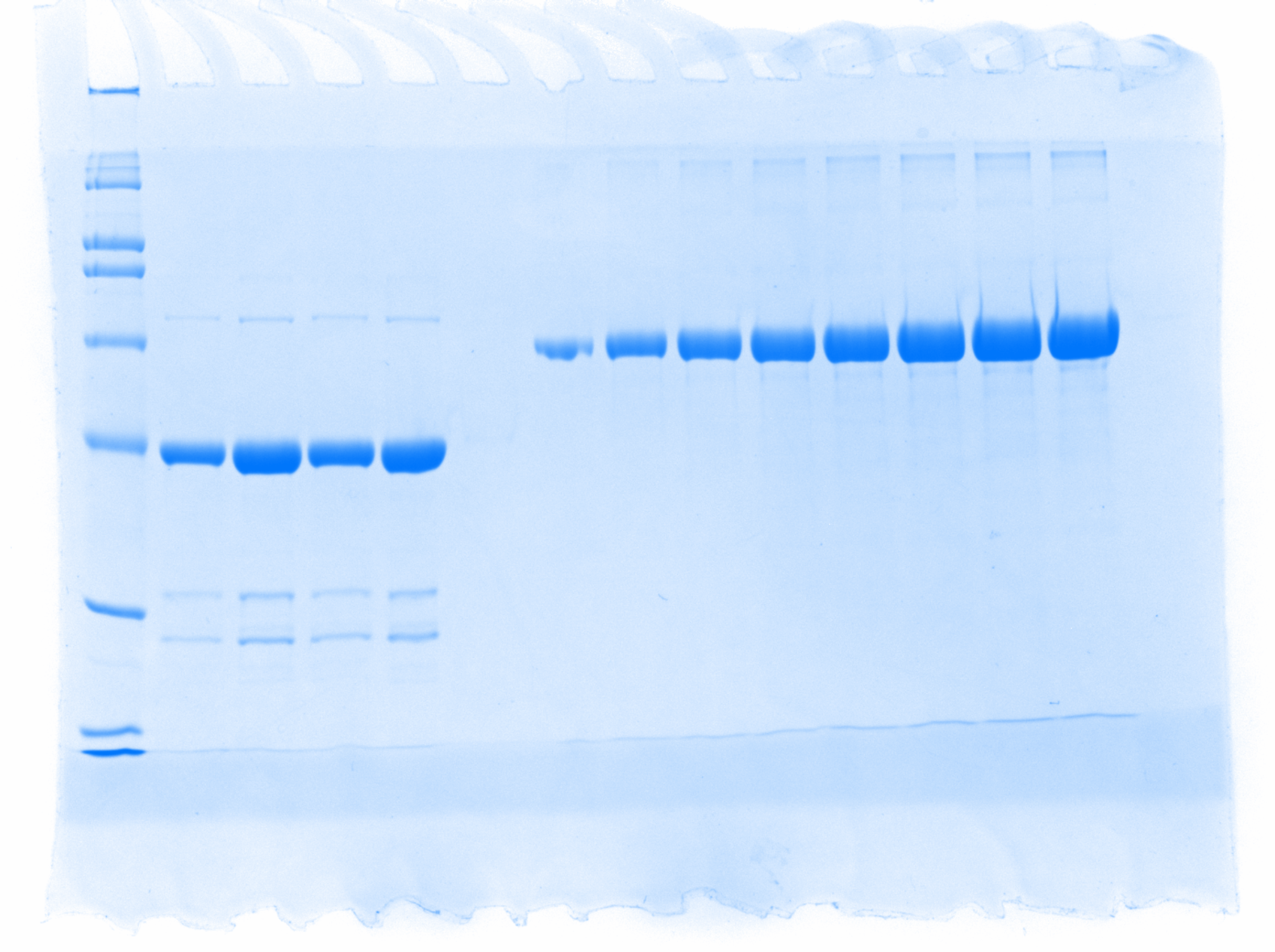

Supplement: Figure 1—source data 1. [file elife-88836-fig1-data1.zip › Figure 1- source data 1/HRAS final.tif]

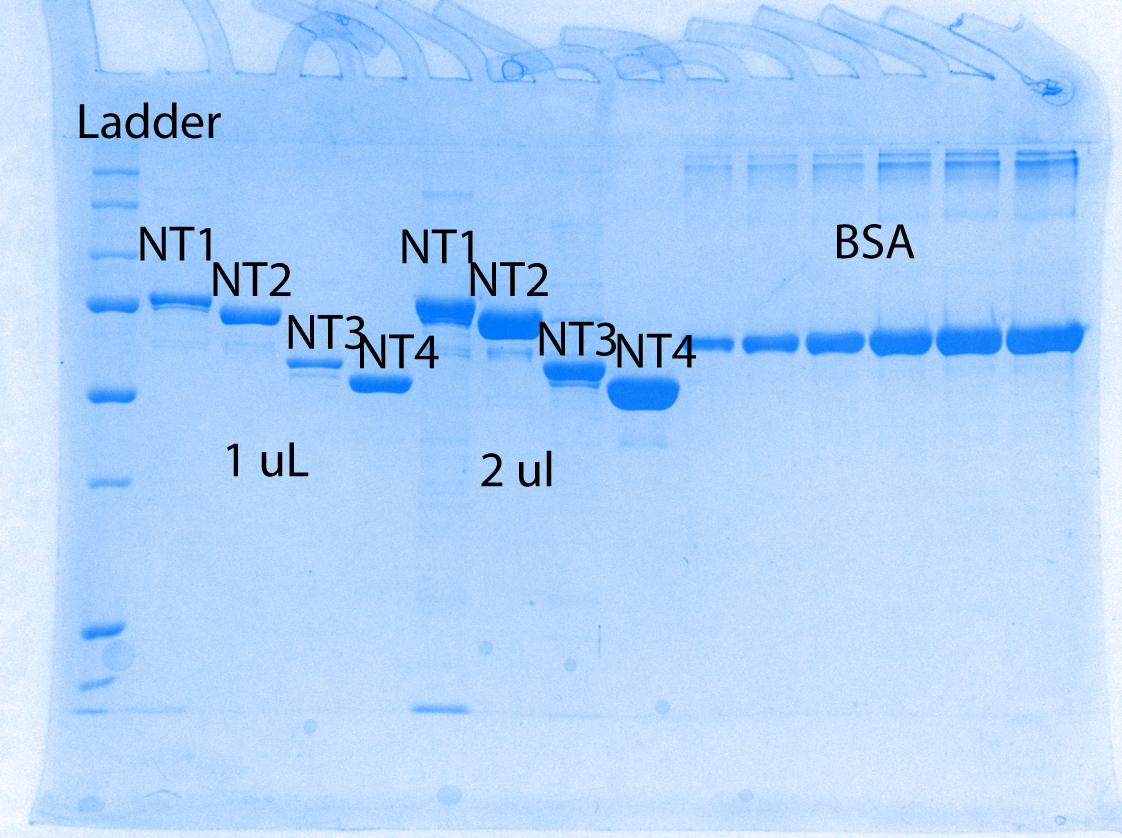

Supplement: Figure 1—source data 1. [file elife-88836-fig1-data1.zip › Figure 1- source data 1/BRAF NTs-01.png]

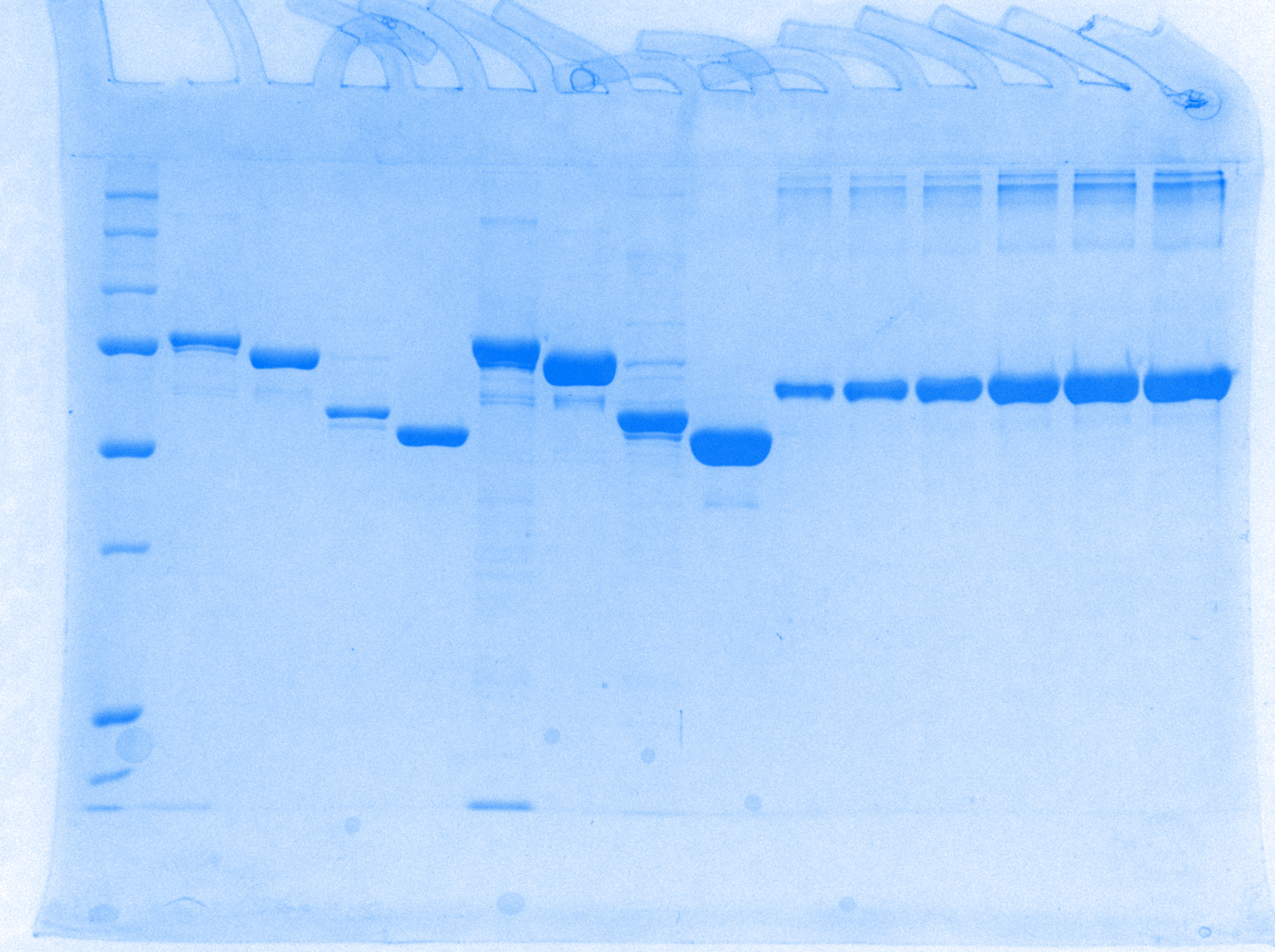

Supplement: Figure 1—source data 1. [file elife-88836-fig1-data1.zip › Figure 1- source data 1/BRAF NTs.tif]

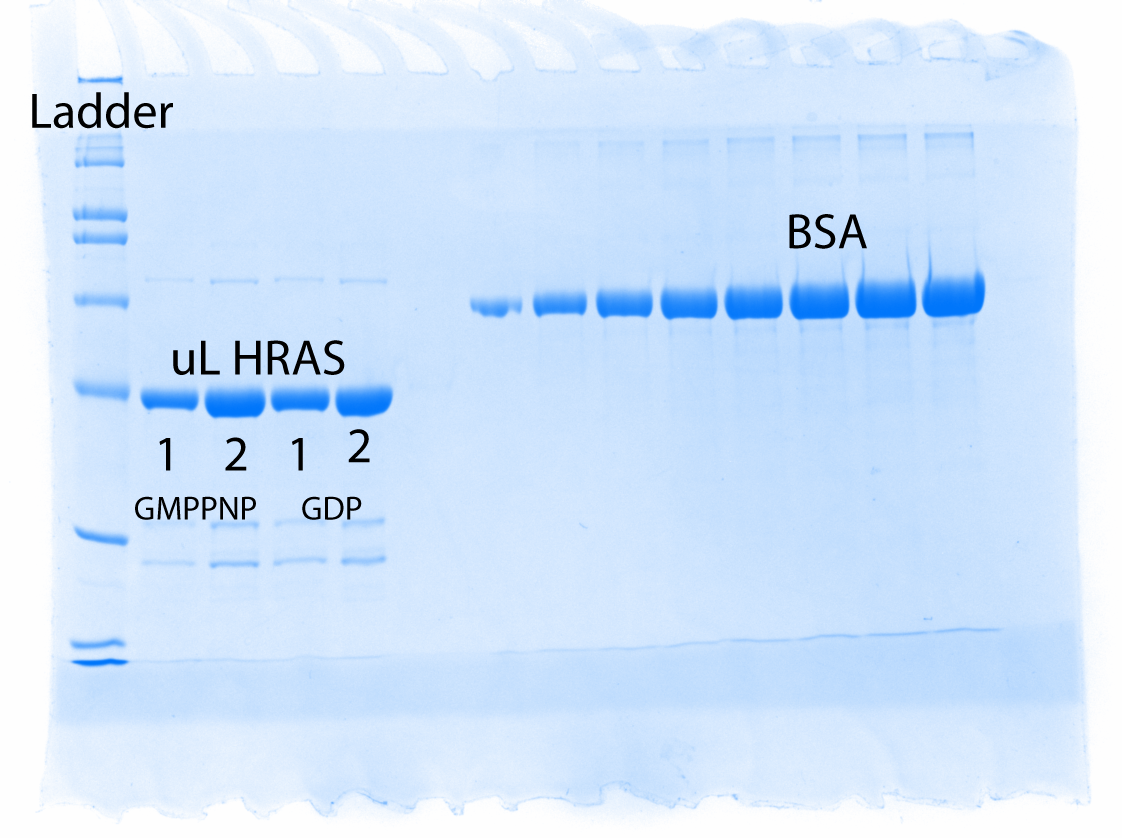

Supplement: Figure 1—source data 1. [file elife-88836-fig1-data1.zip › Figure 1- source data 1/HRAS final-01.png]

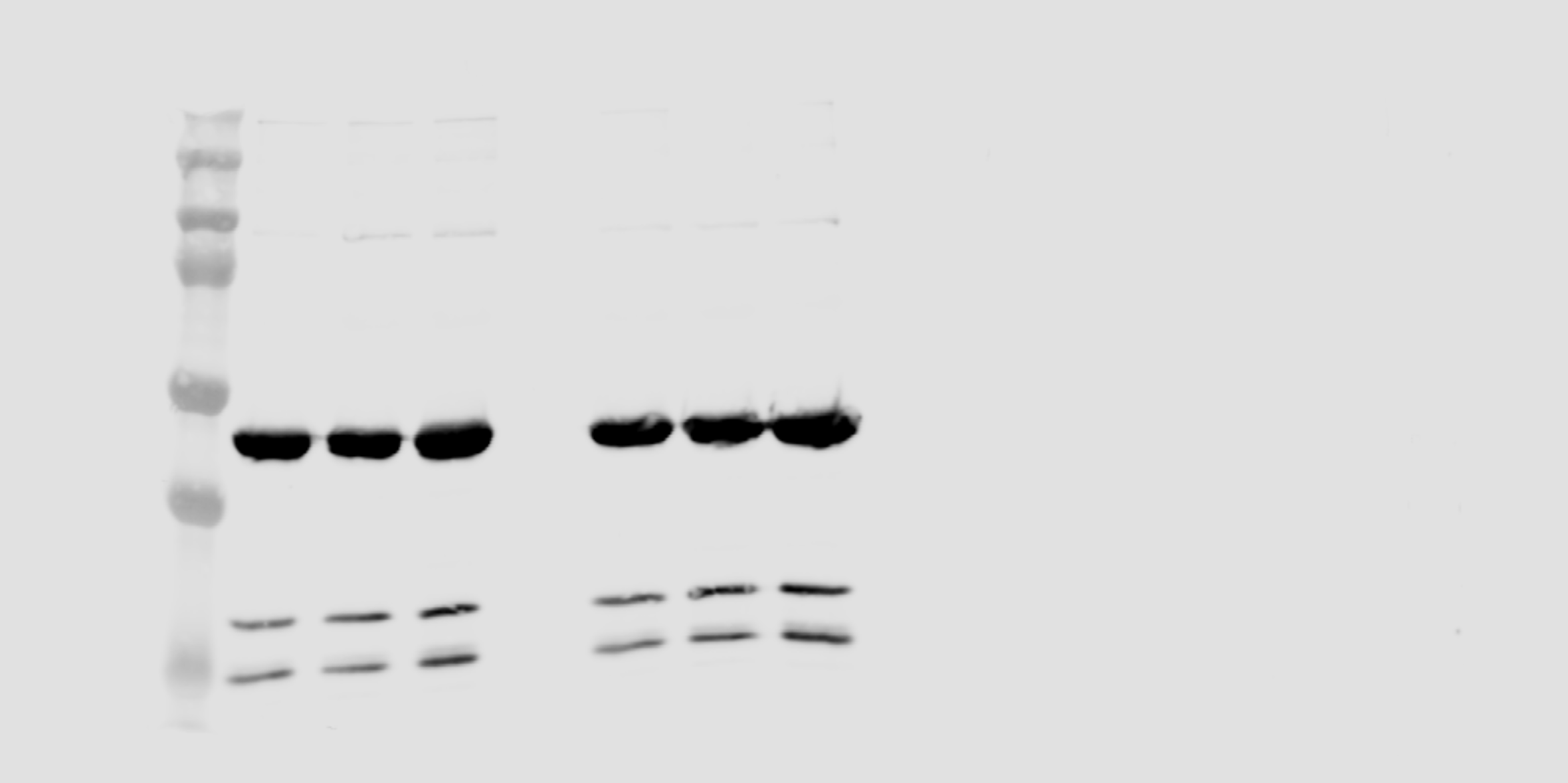

Supplement: Figure 1—source data 2. — HRAS probed with anti-GST antibody; NT1 probed with anti-His antibody. [file elife-88836-fig1-data2.zip › Figure 1- source data 2/GST-HRAS PD.tif]

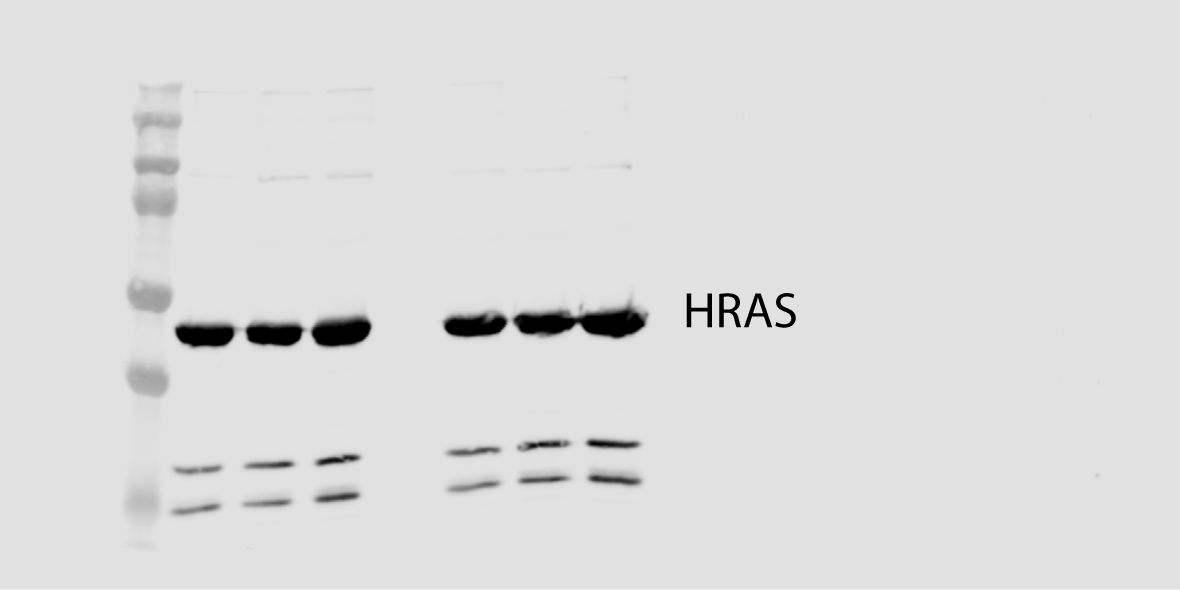

Supplement: Figure 1—source data 2. — HRAS probed with anti-GST antibody; NT1 probed with anti-His antibody. [file elife-88836-fig1-data2.zip › Figure 1- source data 2/GST-HRAS PD-01.png]

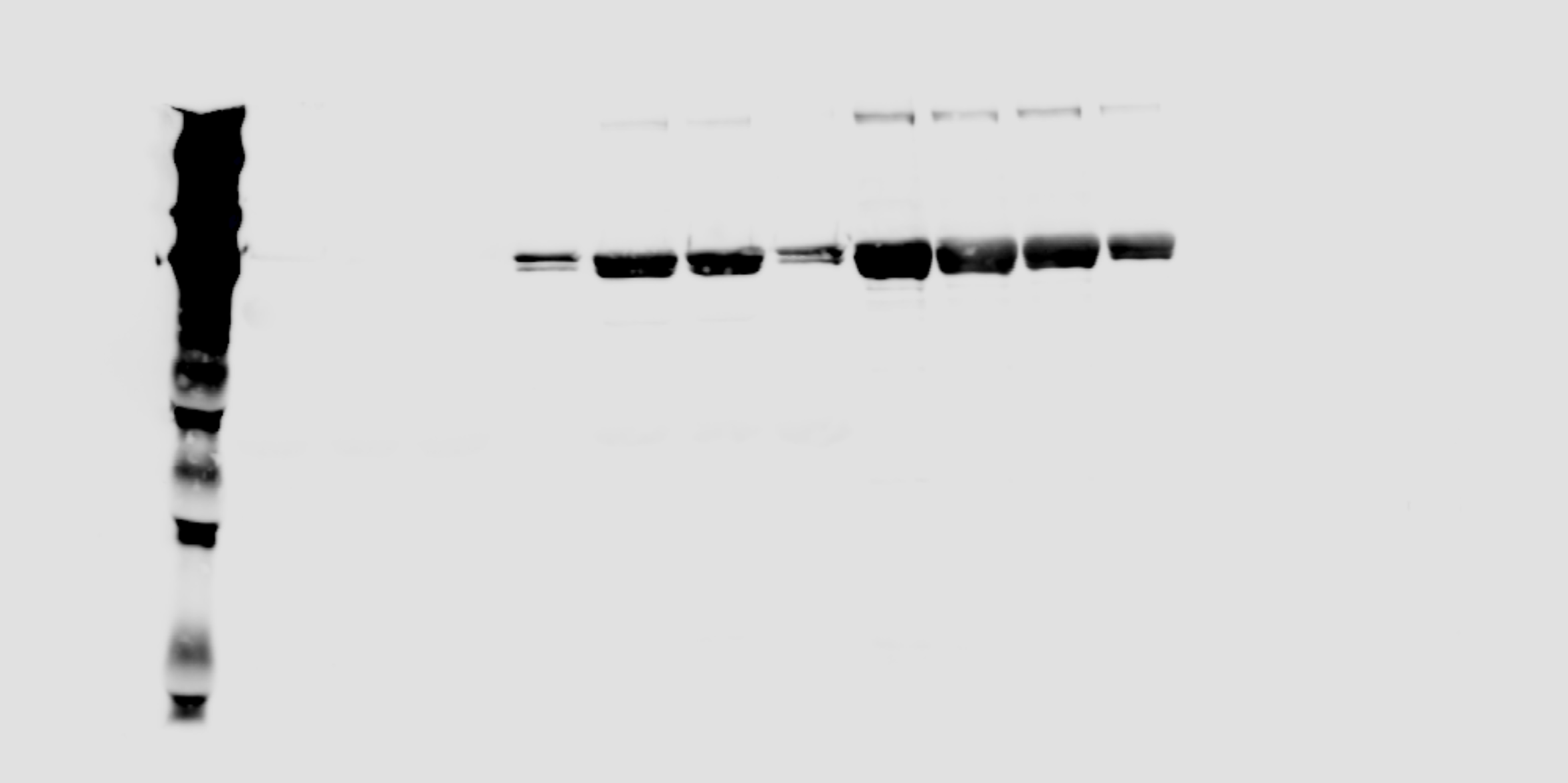

Supplement: Figure 1—source data 2. — HRAS probed with anti-GST antibody; NT1 probed with anti-His antibody. [file elife-88836-fig1-data2.zip › Figure 1- source data 2/NT1 PD.tif]

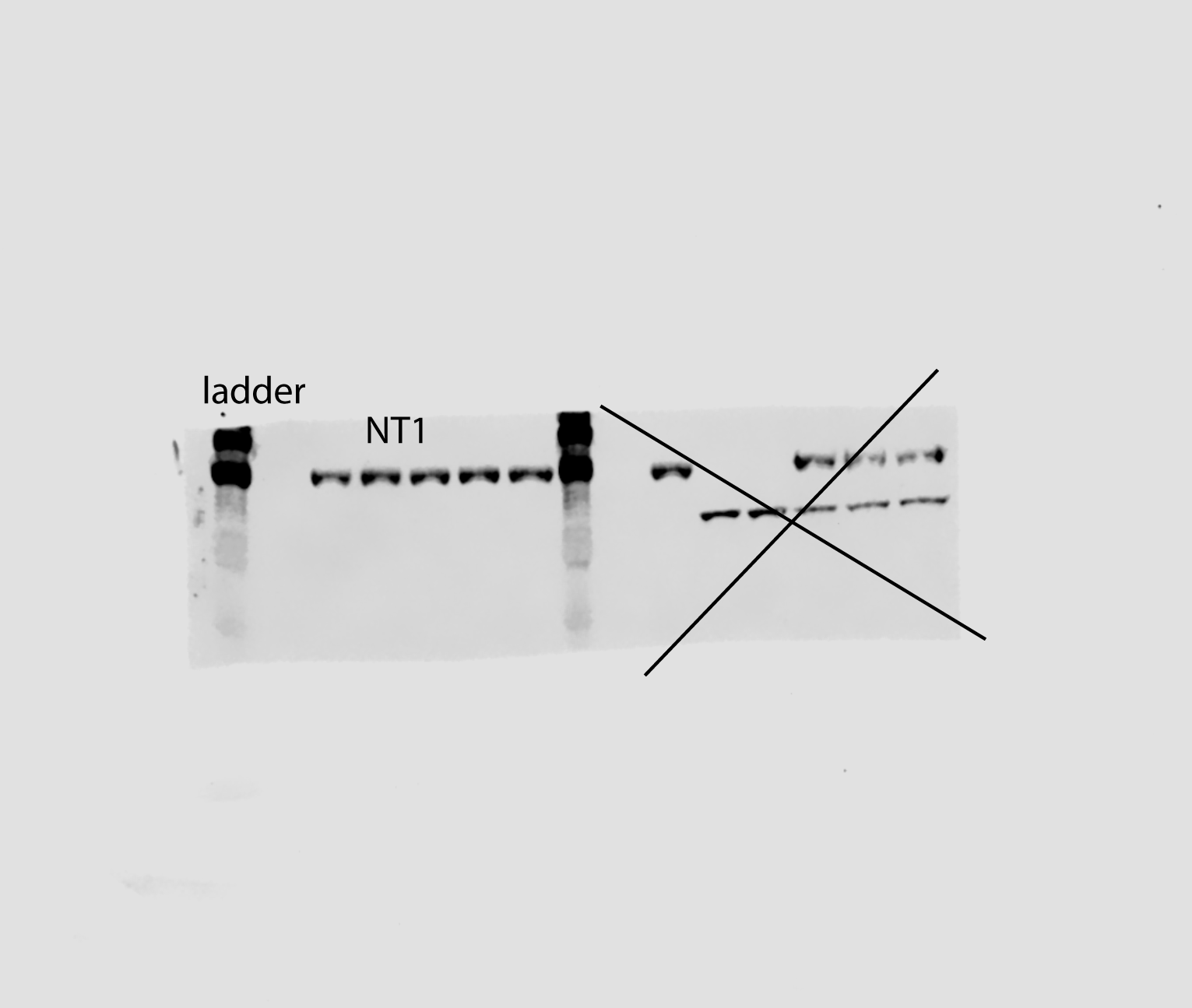

Supplement: Figure 1—source data 2. — HRAS probed with anti-GST antibody; NT1 probed with anti-His antibody. [file elife-88836-fig1-data2.zip › Figure 1- source data 2/NT1 input (left)-01.png]

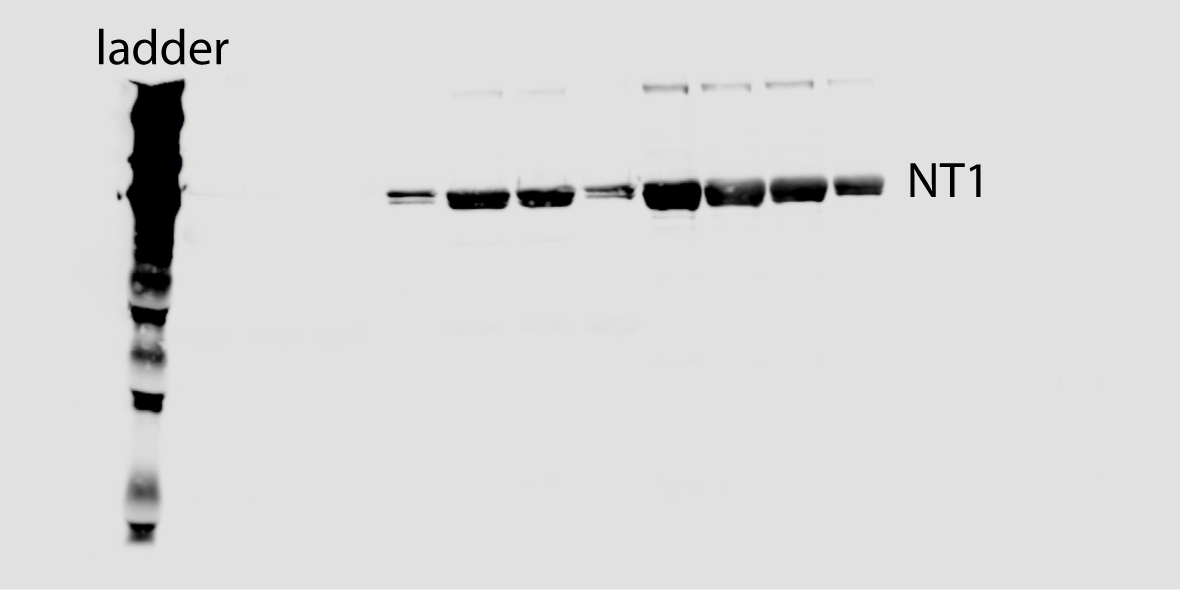

Supplement: Figure 1—source data 2. — HRAS probed with anti-GST antibody; NT1 probed with anti-His antibody. [file elife-88836-fig1-data2.zip › Figure 1- source data 2/NT1 PD-01.png]

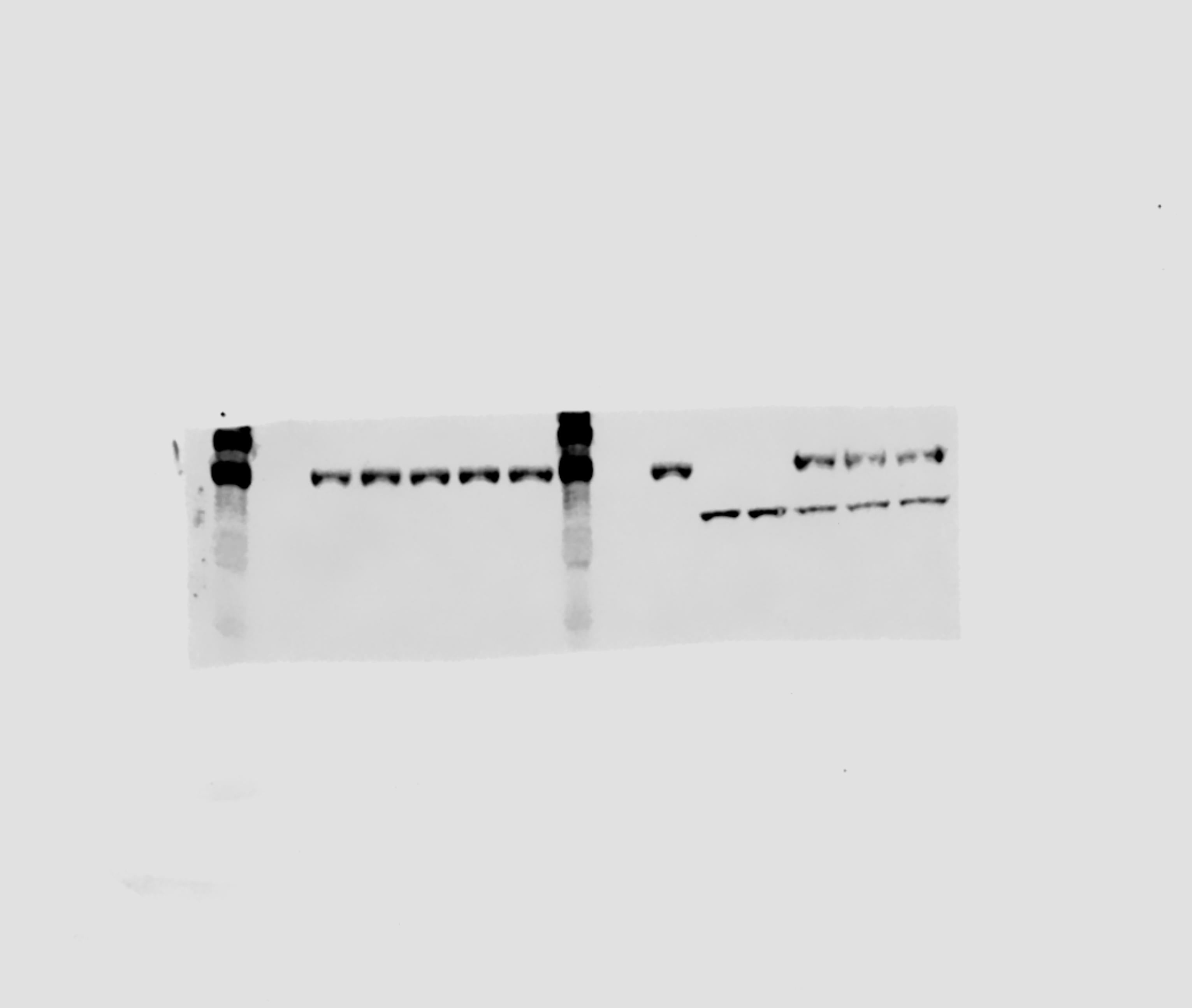

Supplement: Figure 1—source data 2. — HRAS probed with anti-GST antibody; NT1 probed with anti-His antibody. [file elife-88836-fig1-data2.zip › Figure 1- source data 2/NT1 input (left).tif]

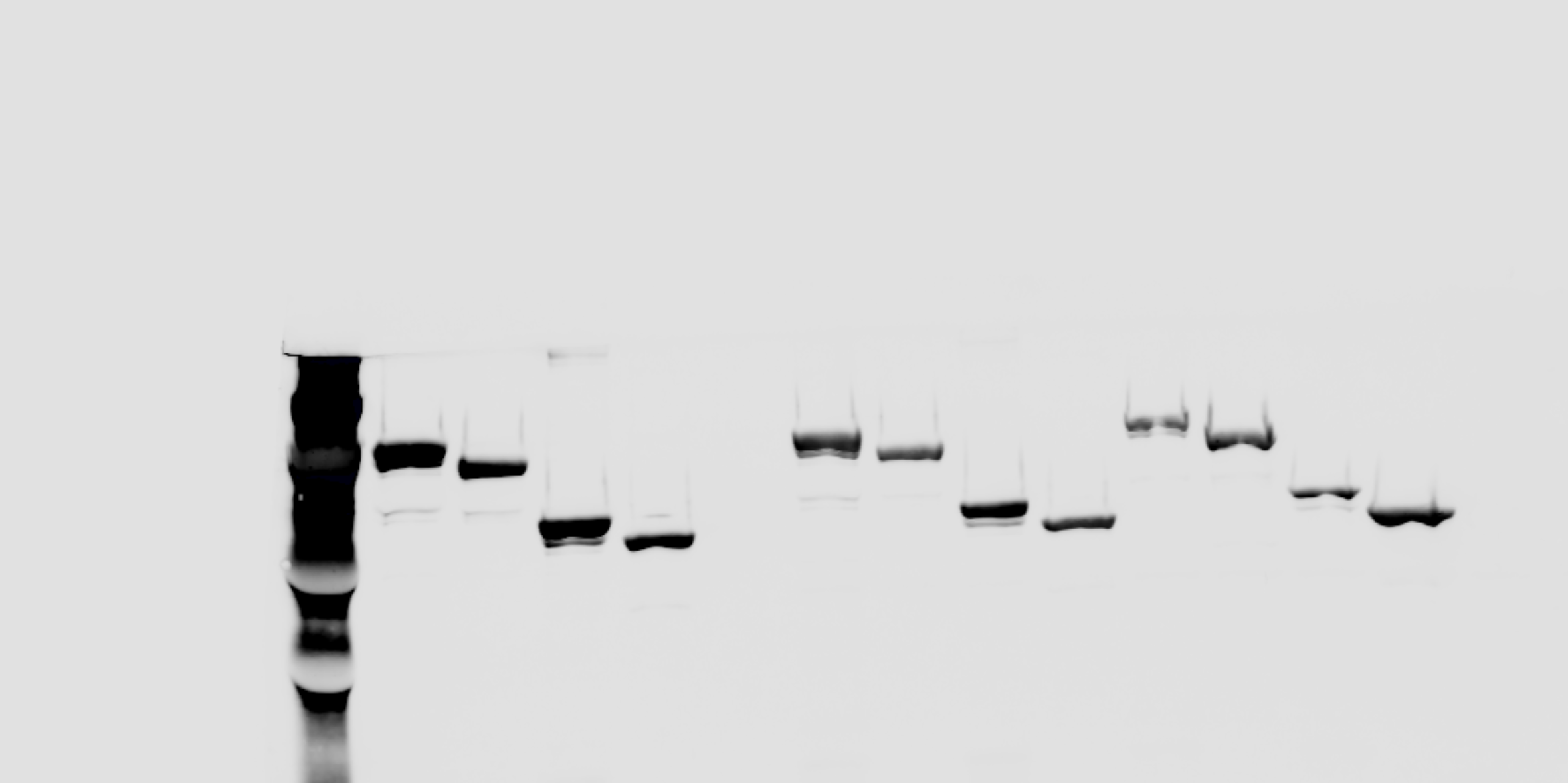

Supplement: Figure 1—source data 3. — HRAS probed with anti-GST antibody; NT1 probed with anti-His antibody. [file elife-88836-fig1-data3.zip › Figure 1- source data 3/NTs PD.tif]

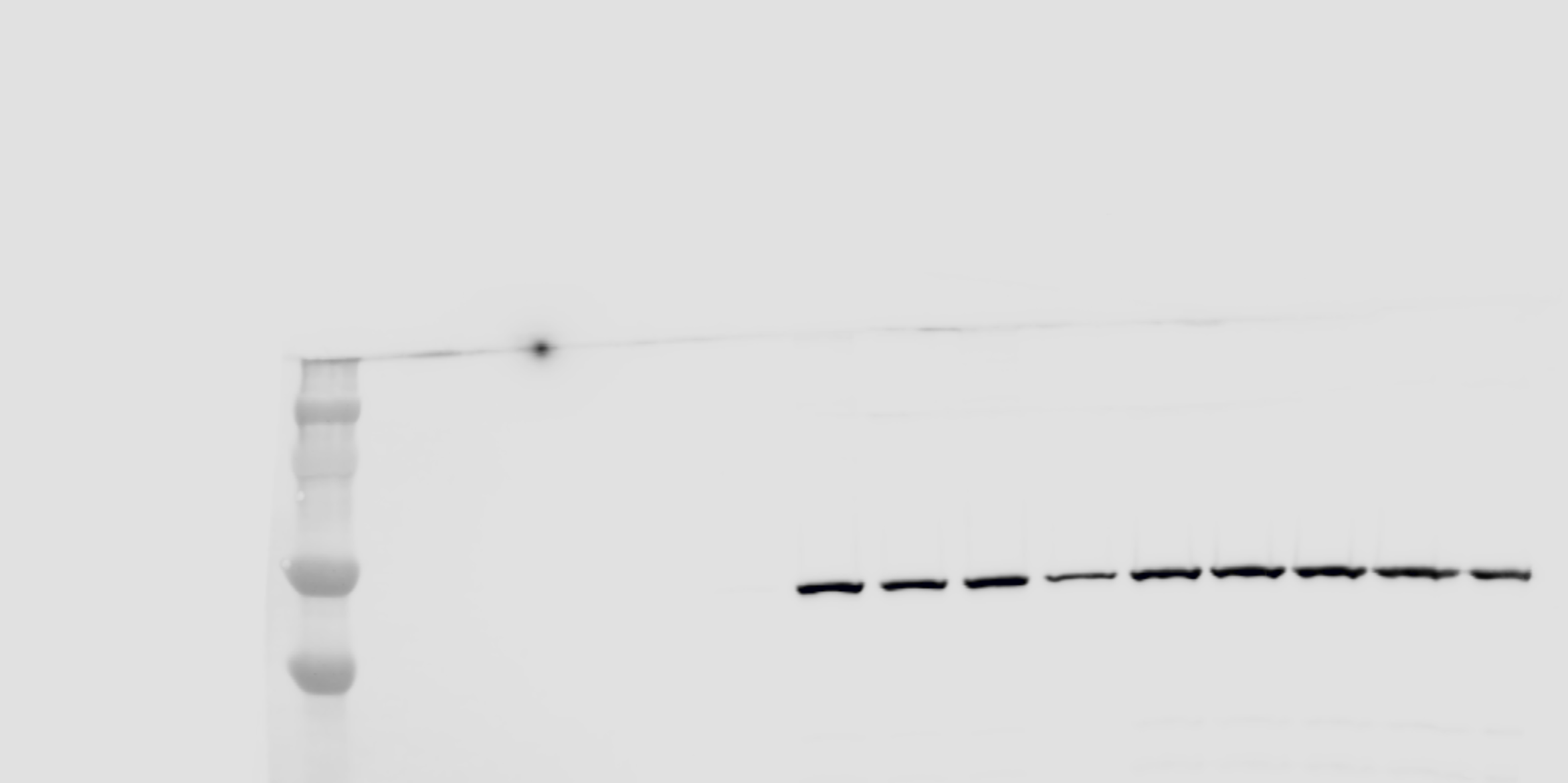

Supplement: Figure 1—source data 3. — HRAS probed with anti-GST antibody; NT1 probed with anti-His antibody. [file elife-88836-fig1-data3.zip › Figure 1- source data 3/GST-HRAS PD.tif]

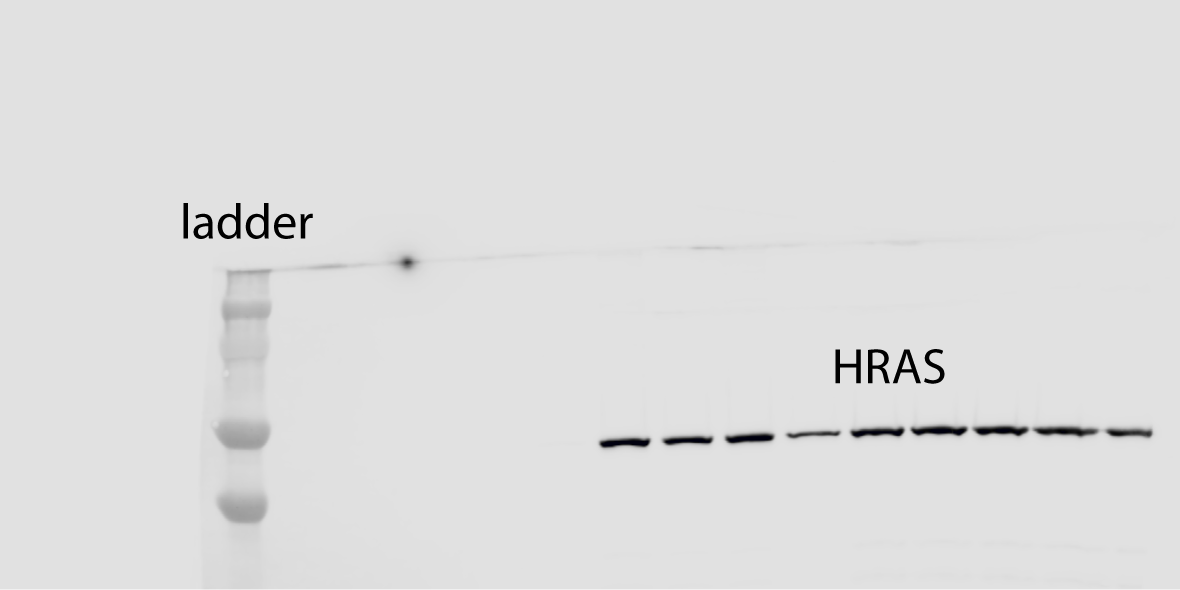

Supplement: Figure 1—source data 3. — HRAS probed with anti-GST antibody; NT1 probed with anti-His antibody. [file elife-88836-fig1-data3.zip › Figure 1- source data 3/GST-HRAS PD-01.png]

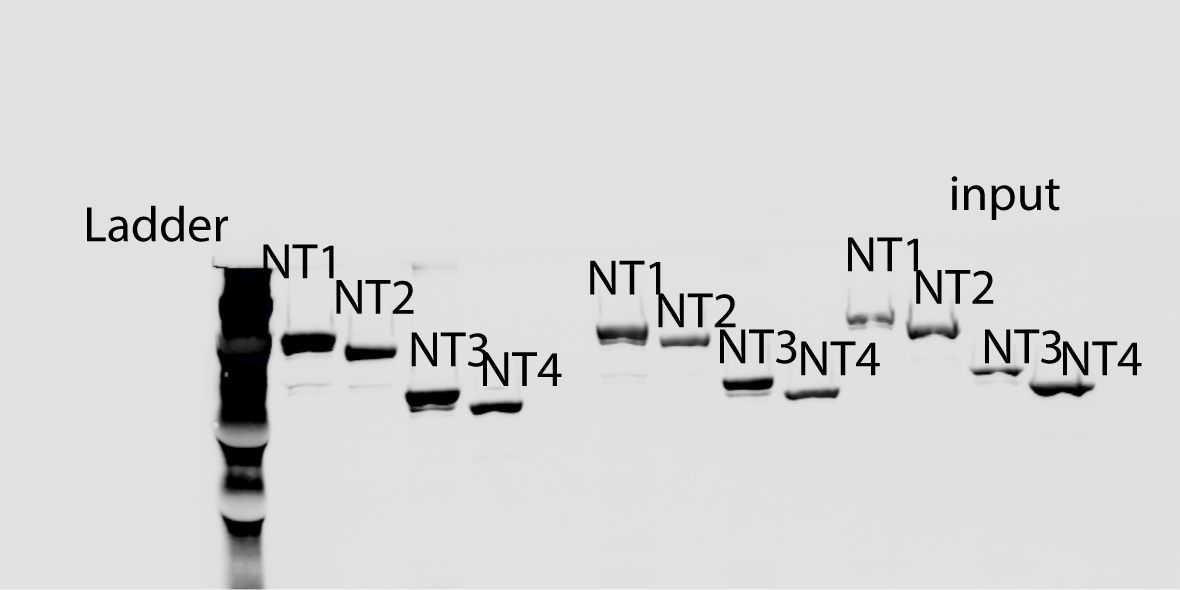

Supplement: Figure 1—source data 3. — HRAS probed with anti-GST antibody; NT1 probed with anti-His antibody. [file elife-88836-fig1-data3.zip › Figure 1- source data 3/NTs PD-01.png]

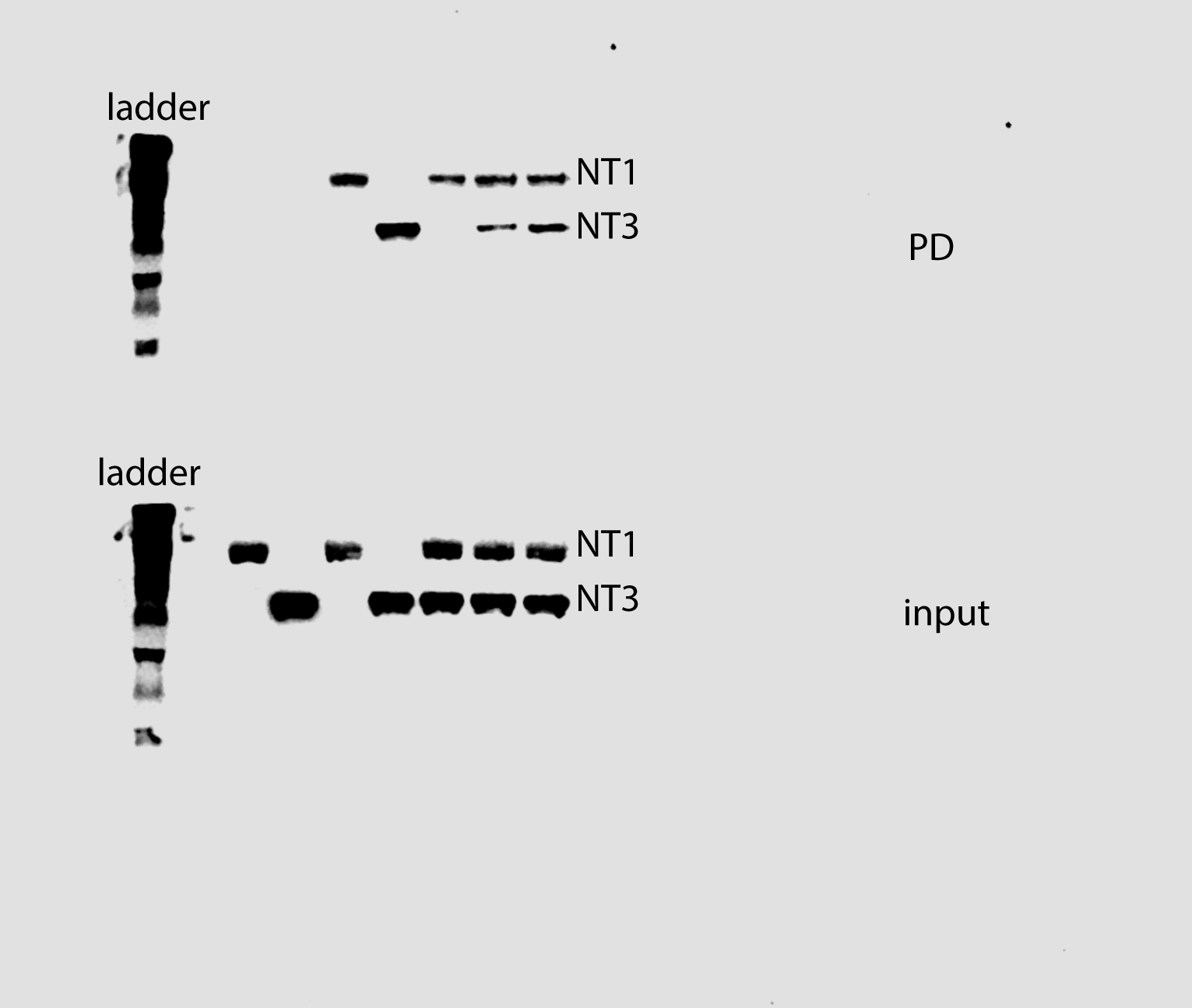

Supplement: Figure 3—source data 2. — HRAS probed with anti-GST antibody; NT1 probed with anti-His antibody. [file elife-88836-fig3-data2.zip › Figure 3- source data 2/NT1_NT3 PD (top) INPUT (bottom)-01.png]

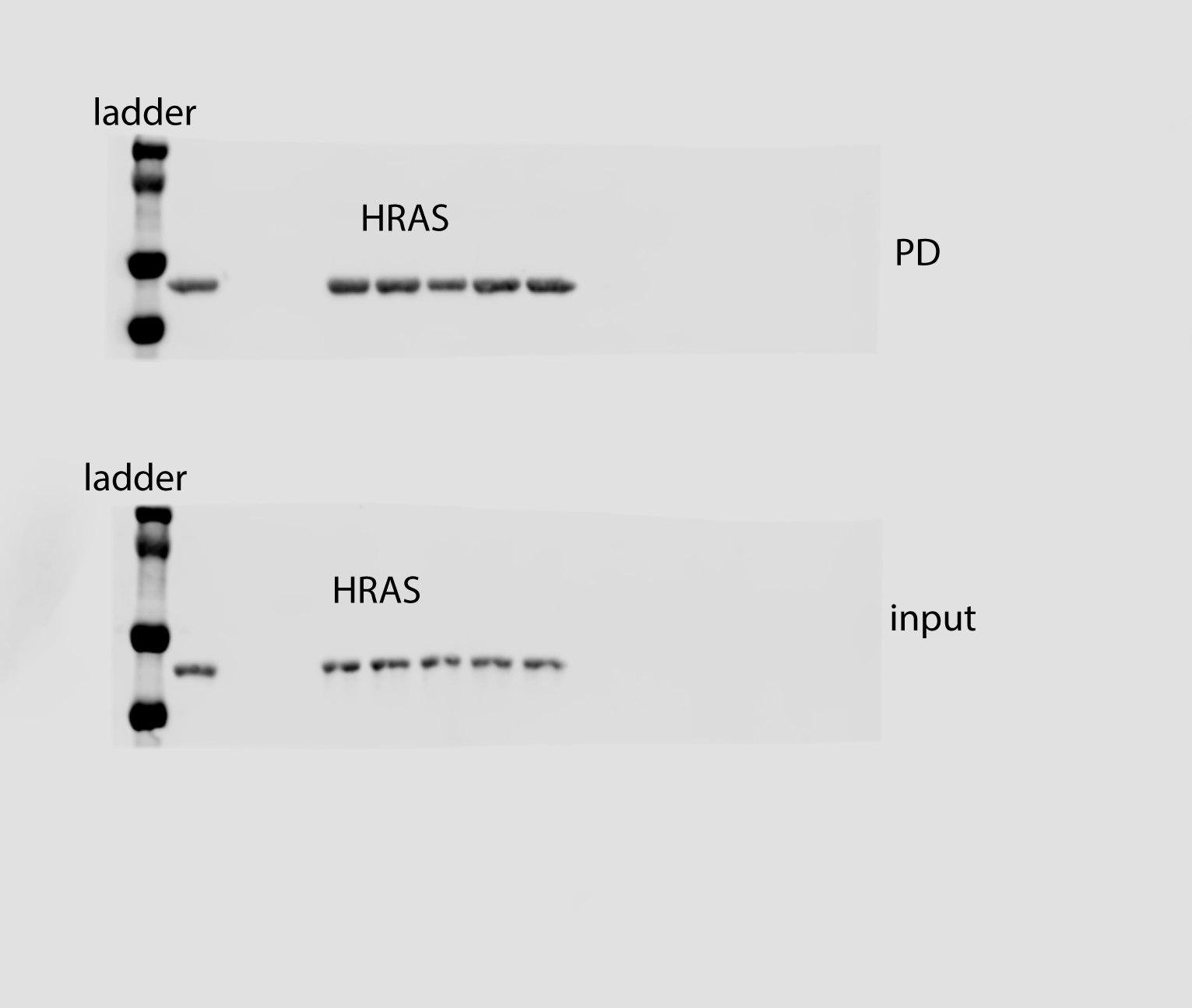

Supplement: Figure 3—source data 2. — HRAS probed with anti-GST antibody; NT1 probed with anti-His antibody. [file elife-88836-fig3-data2.zip › Figure 3- source data 2/GST-HRAS PD (top) INPUT (bottom)-01.png]

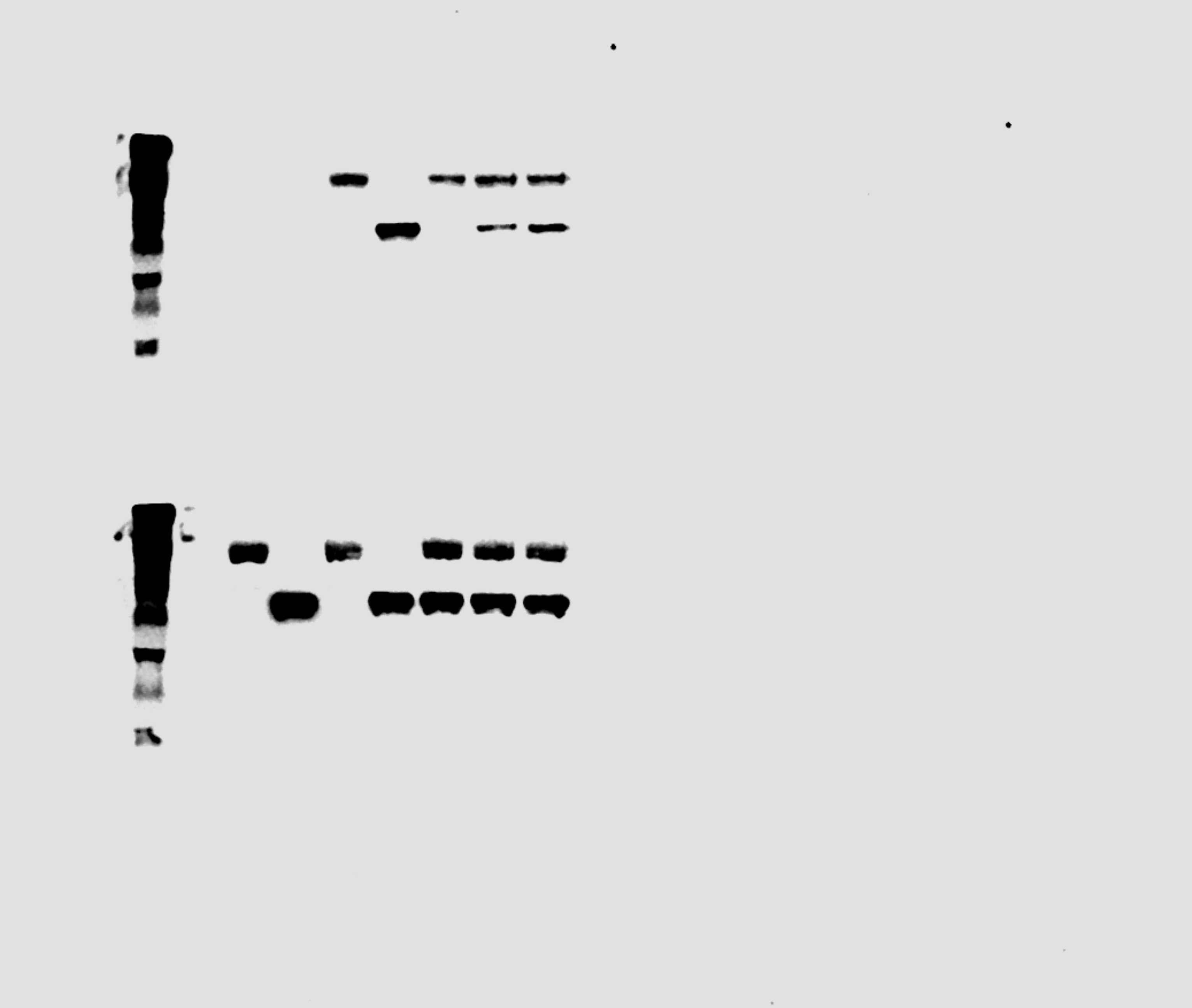

Supplement: Figure 3—source data 2. — HRAS probed with anti-GST antibody; NT1 probed with anti-His antibody. [file elife-88836-fig3-data2.zip › Figure 3- source data 2/NT1_NT3 PD (top) INPUT (bottom).tif]

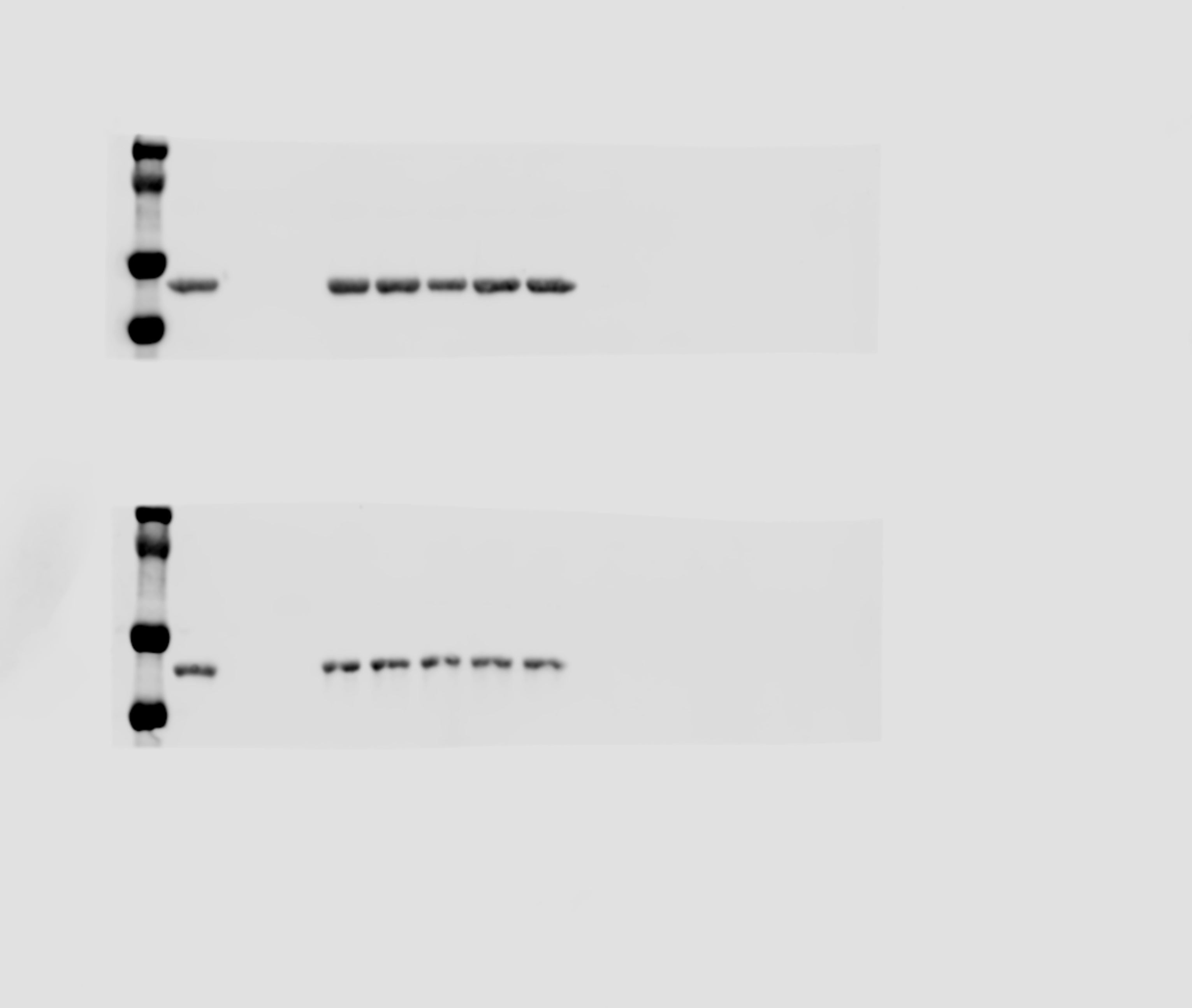

Supplement: Figure 3—source data 2. — HRAS probed with anti-GST antibody; NT1 probed with anti-His antibody. [file elife-88836-fig3-data2.zip › Figure 3- source data 2/GST-HRAS PD (top) INPUT (bottom).tif]

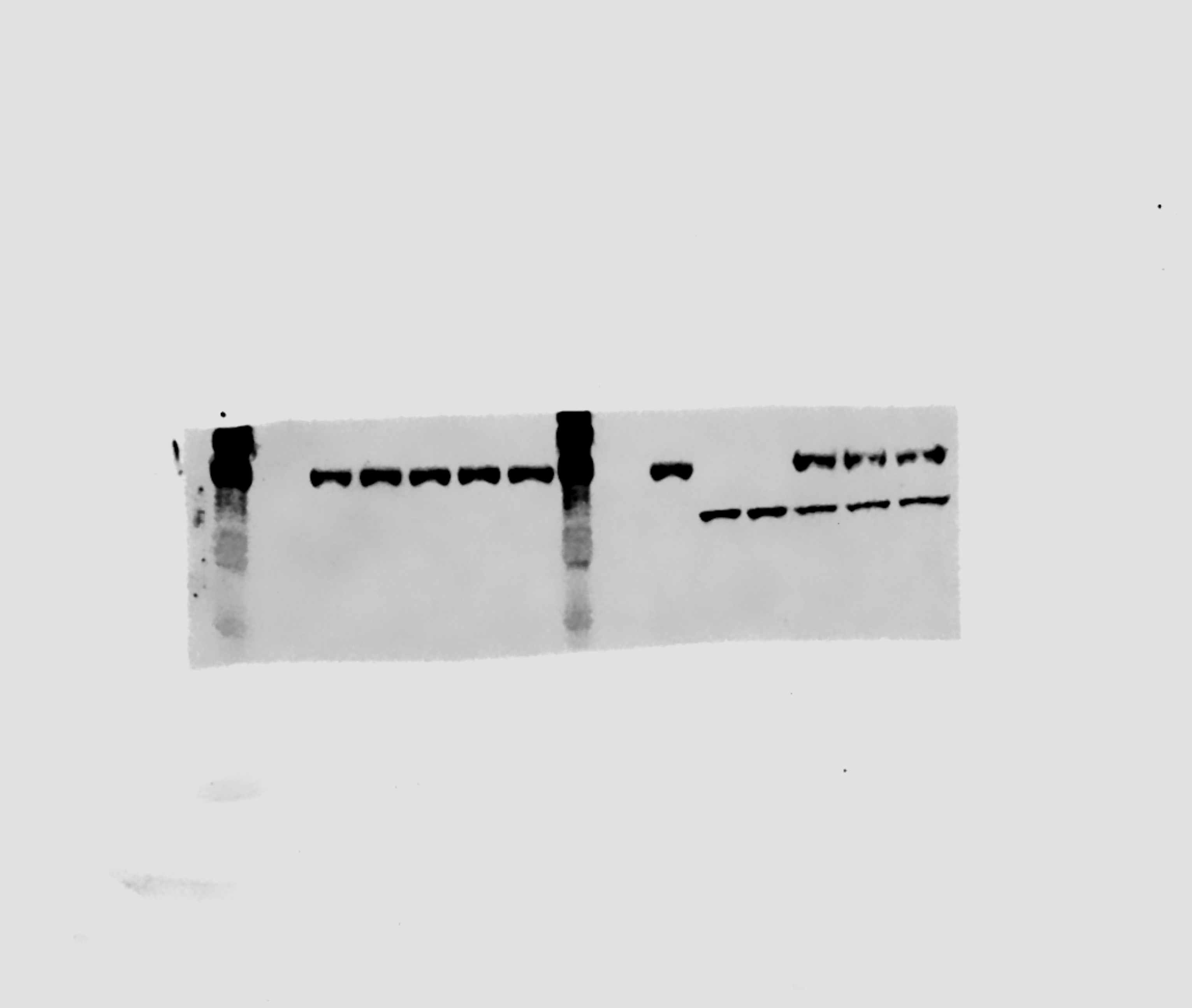

Supplement: Figure 3—source data 3. — HRAS probed with anti-GST antibody; NT1 probed with anti-His antibody. [file elife-88836-fig3-data3.zip › Figure 3- source data 3/NT1_NT3 INPUT (right).tif]

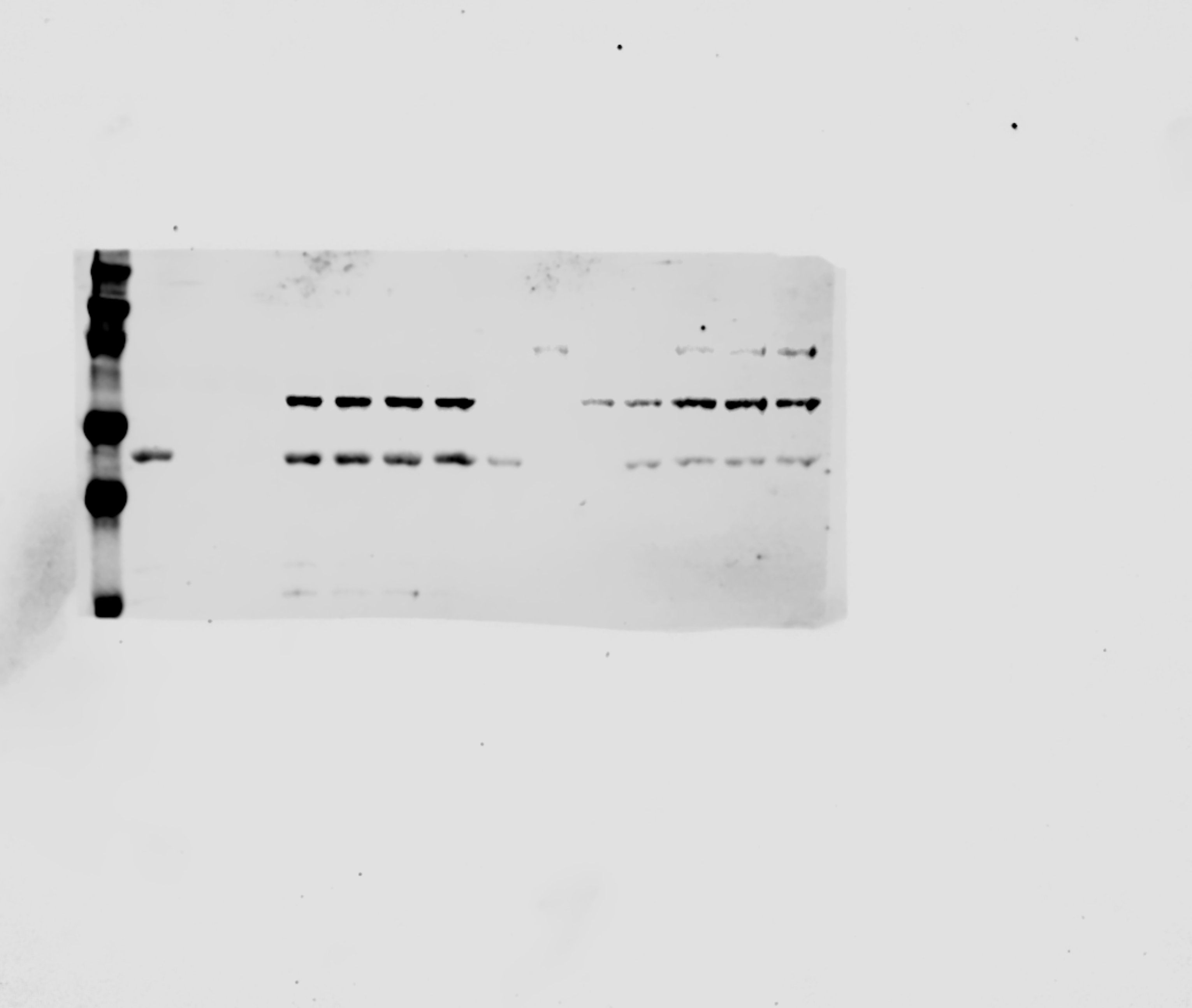

Supplement: Figure 3—source data 3. — HRAS probed with anti-GST antibody; NT1 probed with anti-His antibody. [file elife-88836-fig3-data3.zip › Figure 3- source data 3/HRAS_NT1vNT3 PD (left).tif]

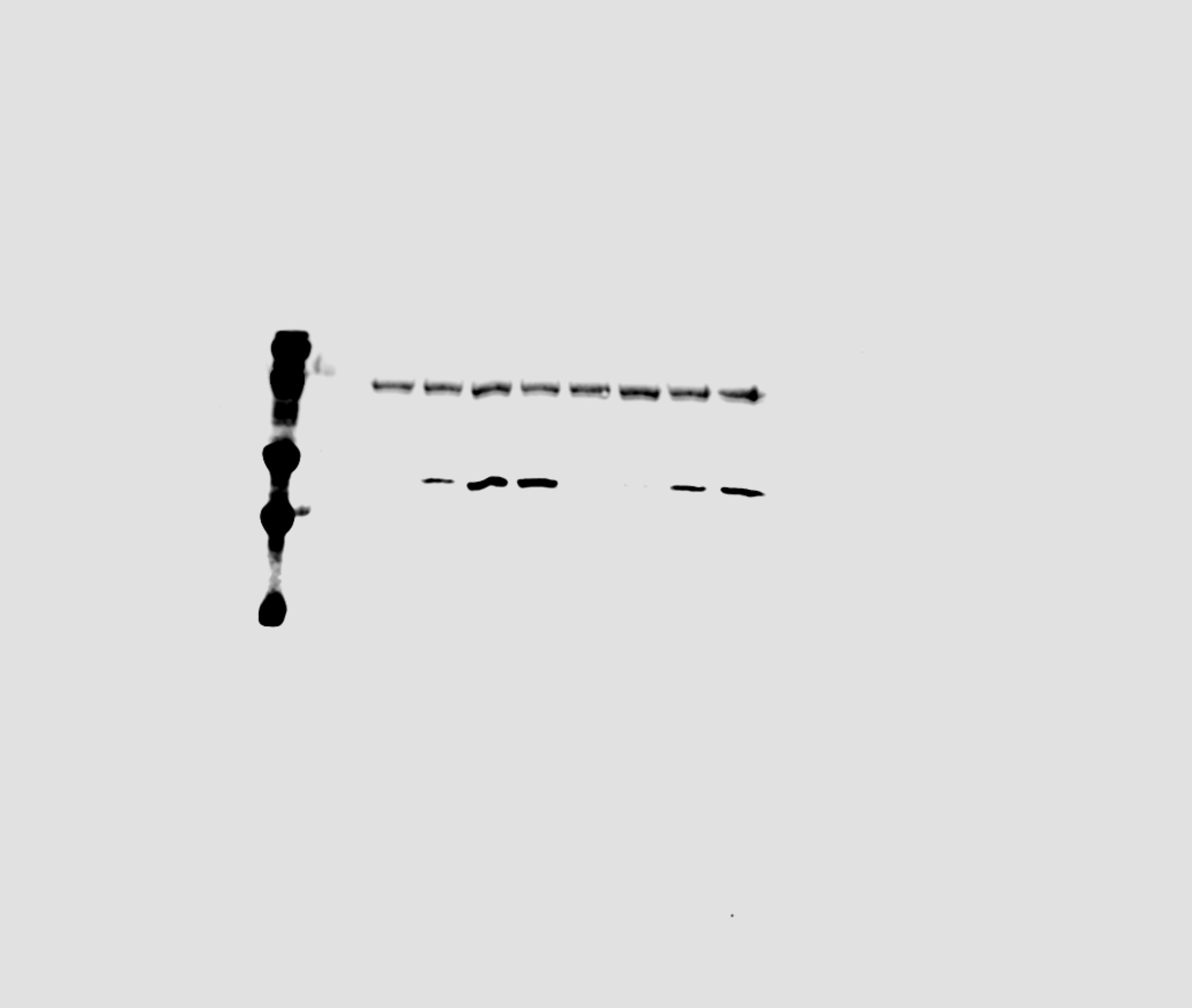

Supplement: Figure 3—figure supplement 1—source data 1. — HRAS was first added to resin for 1 hr. After washing to remove unbound HRAS, NT1 was added in a 1:1 molar ratio and incubated at 4°C for 5, 30, and 60 min. HRAS was probed with GST antibody and NT1 with His antibody. [file elife-88836-fig3-figsupp1-data1.zip › Figure 3- figure supplement 1- source data 1/NT1_HRAS PD_.tif]

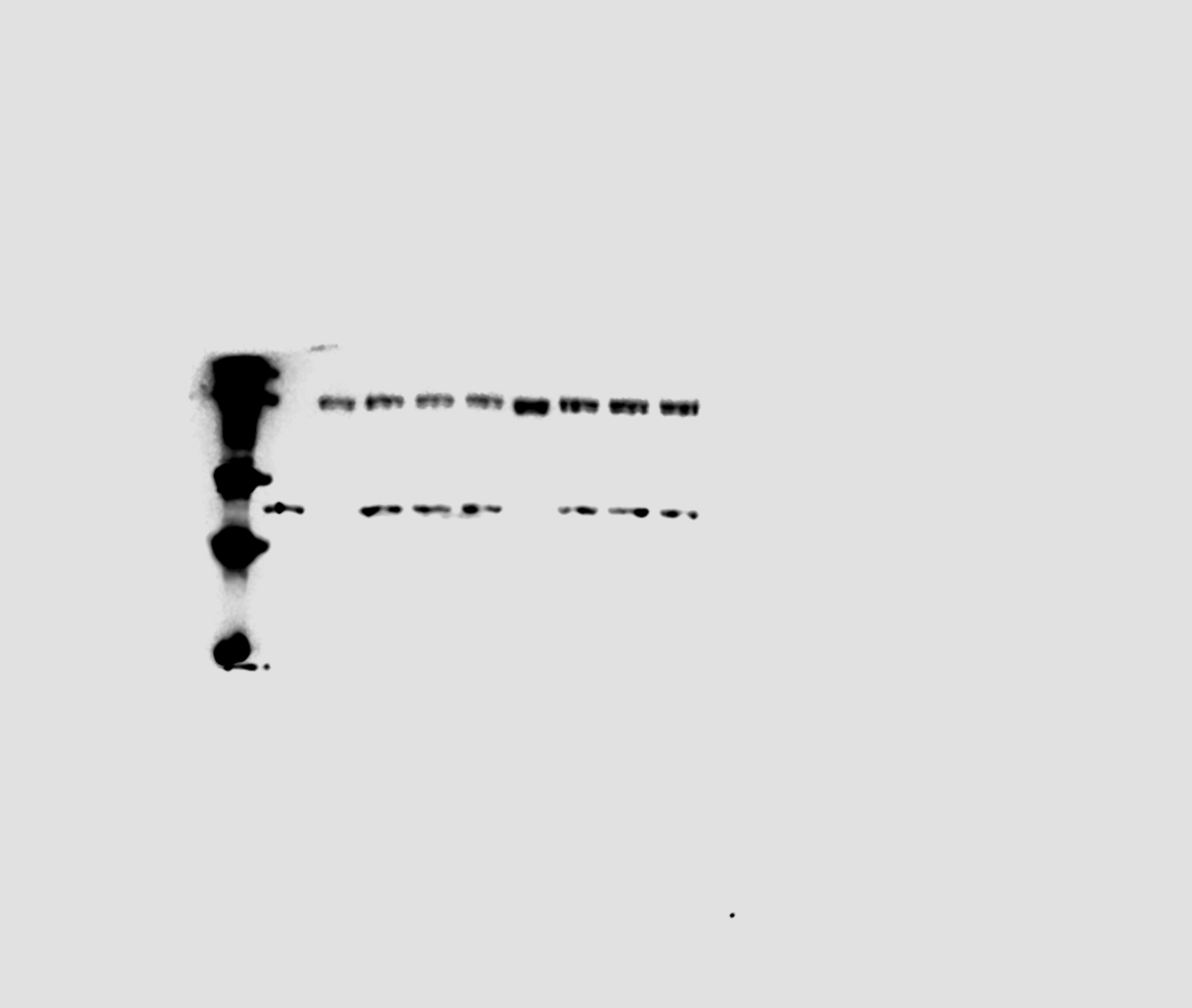

Supplement: Figure 3—figure supplement 1—source data 1. — HRAS was first added to resin for 1 hr. After washing to remove unbound HRAS, NT1 was added in a 1:1 molar ratio and incubated at 4°C for 5, 30, and 60 min. HRAS was probed with GST antibody and NT1 with His antibody. [file elife-88836-fig3-figsupp1-data1.zip › Figure 3- figure supplement 1- source data 1/NT1_HRAS PD input.tif]

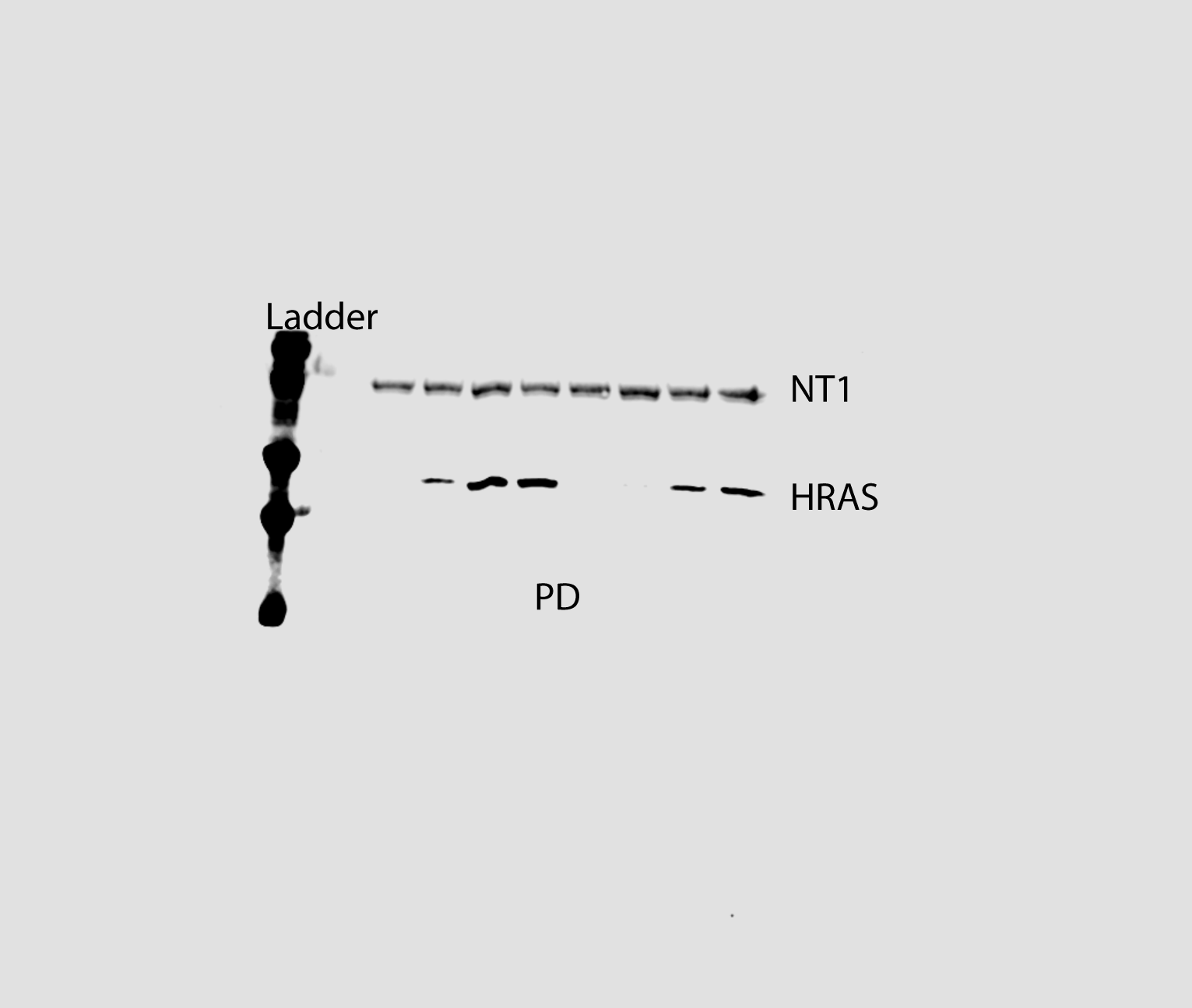

Supplement: Figure 3—figure supplement 1—source data 1. — HRAS was first added to resin for 1 hr. After washing to remove unbound HRAS, NT1 was added in a 1:1 molar ratio and incubated at 4°C for 5, 30, and 60 min. HRAS was probed with GST antibody and NT1 with His antibody. [file elife-88836-fig3-figsupp1-data1.zip › Figure 3- figure supplement 1- source data 1/NT1_HRAS PD_-01.png]

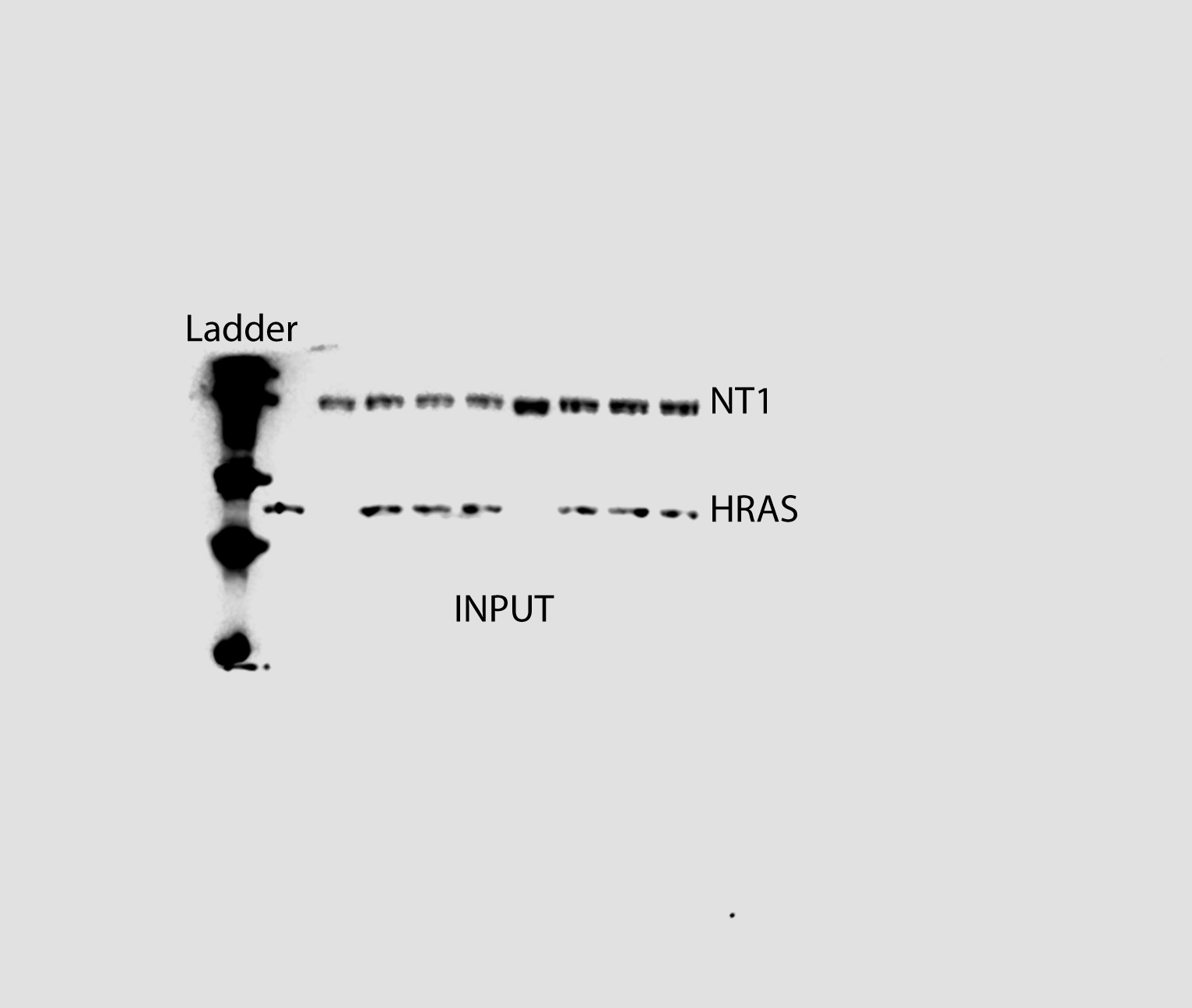

Supplement: Figure 3—figure supplement 1—source data 1. — HRAS was first added to resin for 1 hr. After washing to remove unbound HRAS, NT1 was added in a 1:1 molar ratio and incubated at 4°C for 5, 30, and 60 min. HRAS was probed with GST antibody and NT1 with His antibody. [file elife-88836-fig3-figsupp1-data1.zip › Figure 3- figure supplement 1- source data 1/NT1_HRAS PD input-01.png]

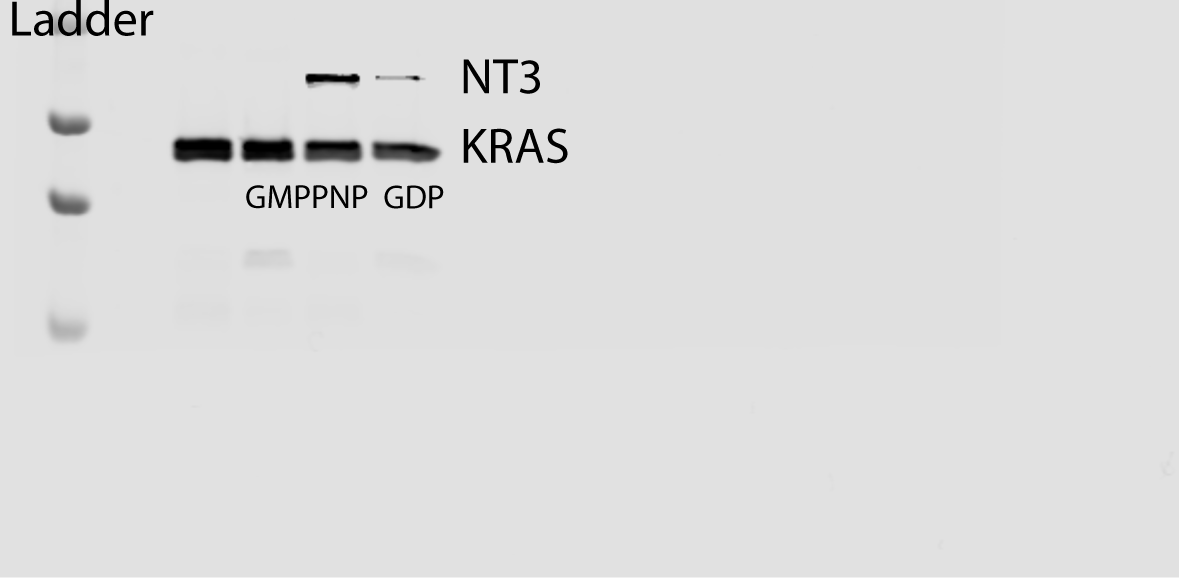

Supplement: Figure 4—source data 1. — KRAS probed with anti-GST antibody; NTs probed with anti-His antibody. [file elife-88836-fig4-data1.zip › Figure 4- source data 1/NT3_GST-KRAS_PD-01.png]

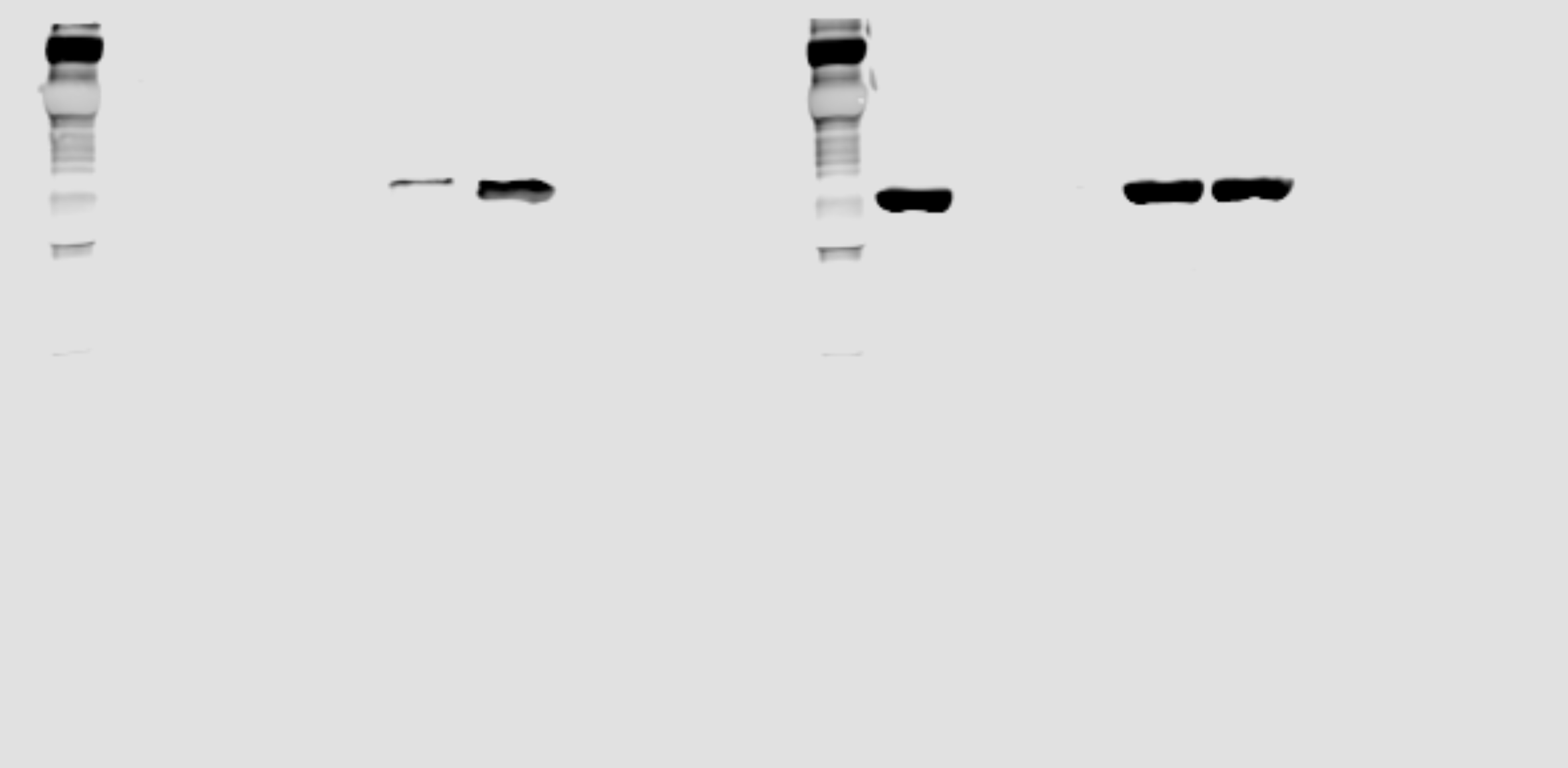

Supplement: Figure 4—source data 1. — KRAS probed with anti-GST antibody; NTs probed with anti-His antibody. [file elife-88836-fig4-data1.zip › Figure 4- source data 1/NT4_GST-KRAS_PD-NT4 left_INPUT-NT4 right.tif]

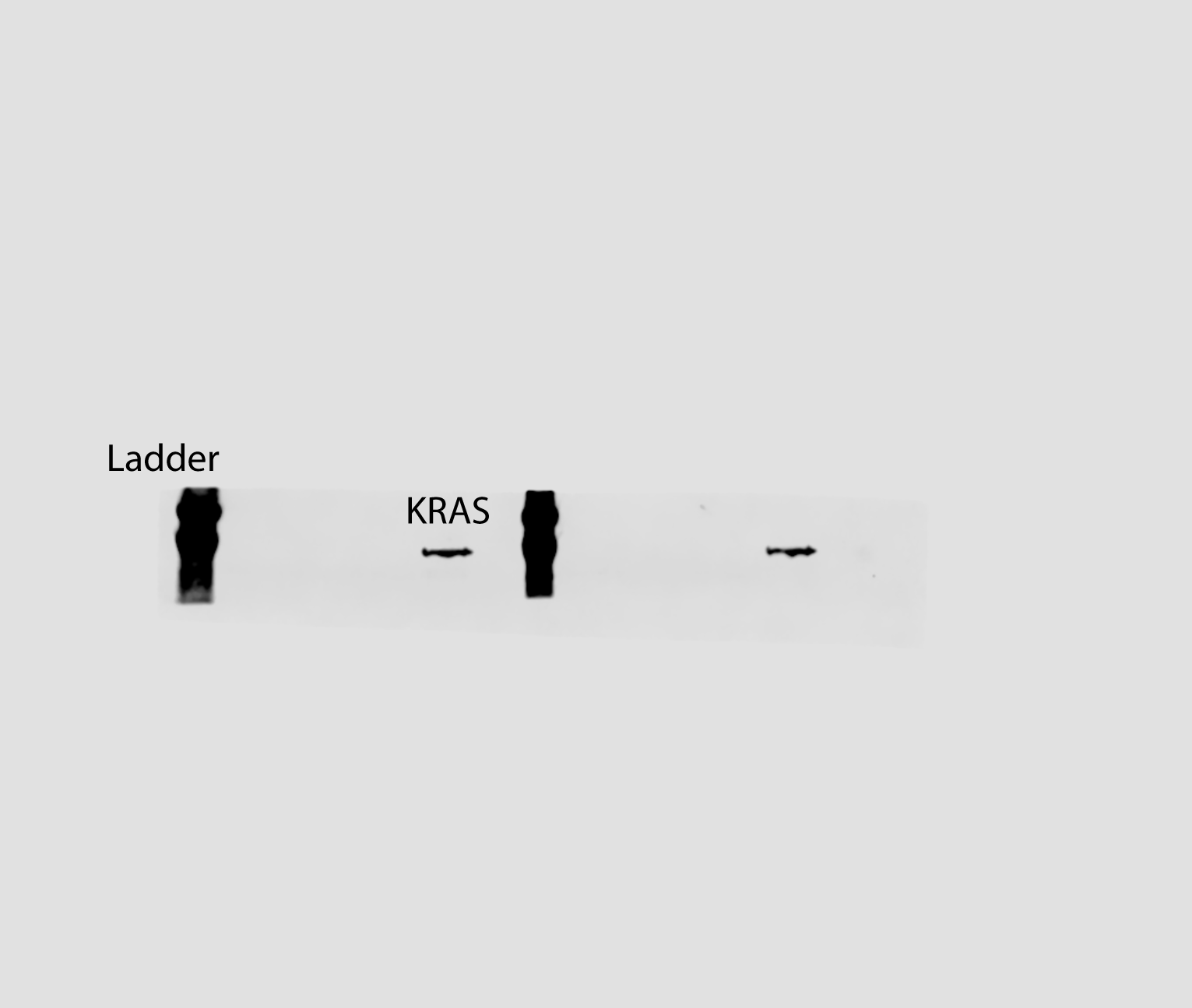

Supplement: Figure 4—source data 1. — KRAS probed with anti-GST antibody; NTs probed with anti-His antibody. [file elife-88836-fig4-data1.zip › Figure 4- source data 1/NT2_GST-KRAS_PDreruns_his_3-2023-01.png]

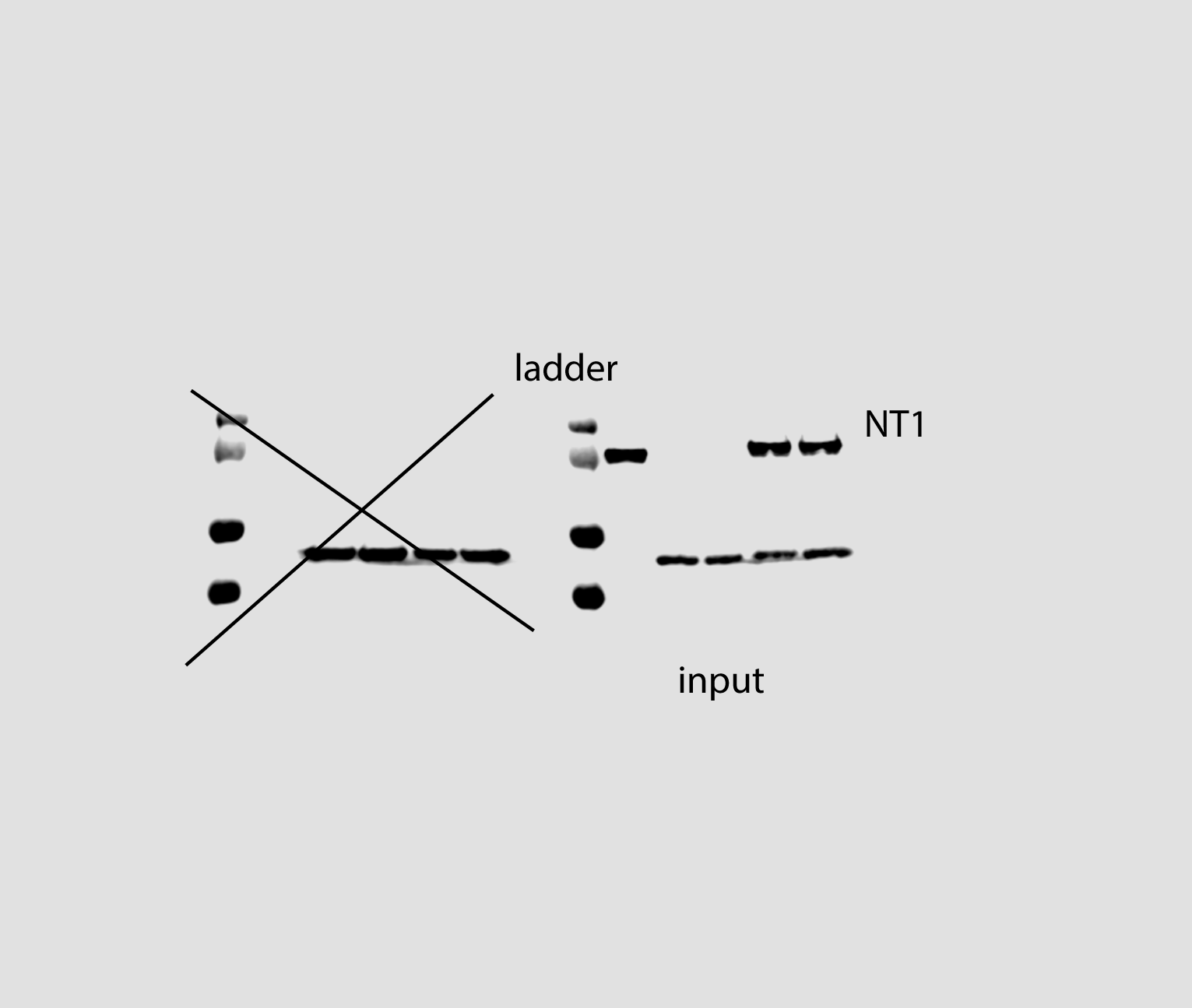

Supplement: Figure 4—source data 1. — KRAS probed with anti-GST antibody; NTs probed with anti-His antibody. [file elife-88836-fig4-data1.zip › Figure 4- source data 1/NT1_GST-KRAS_PD1_input_3-2023-01.png]

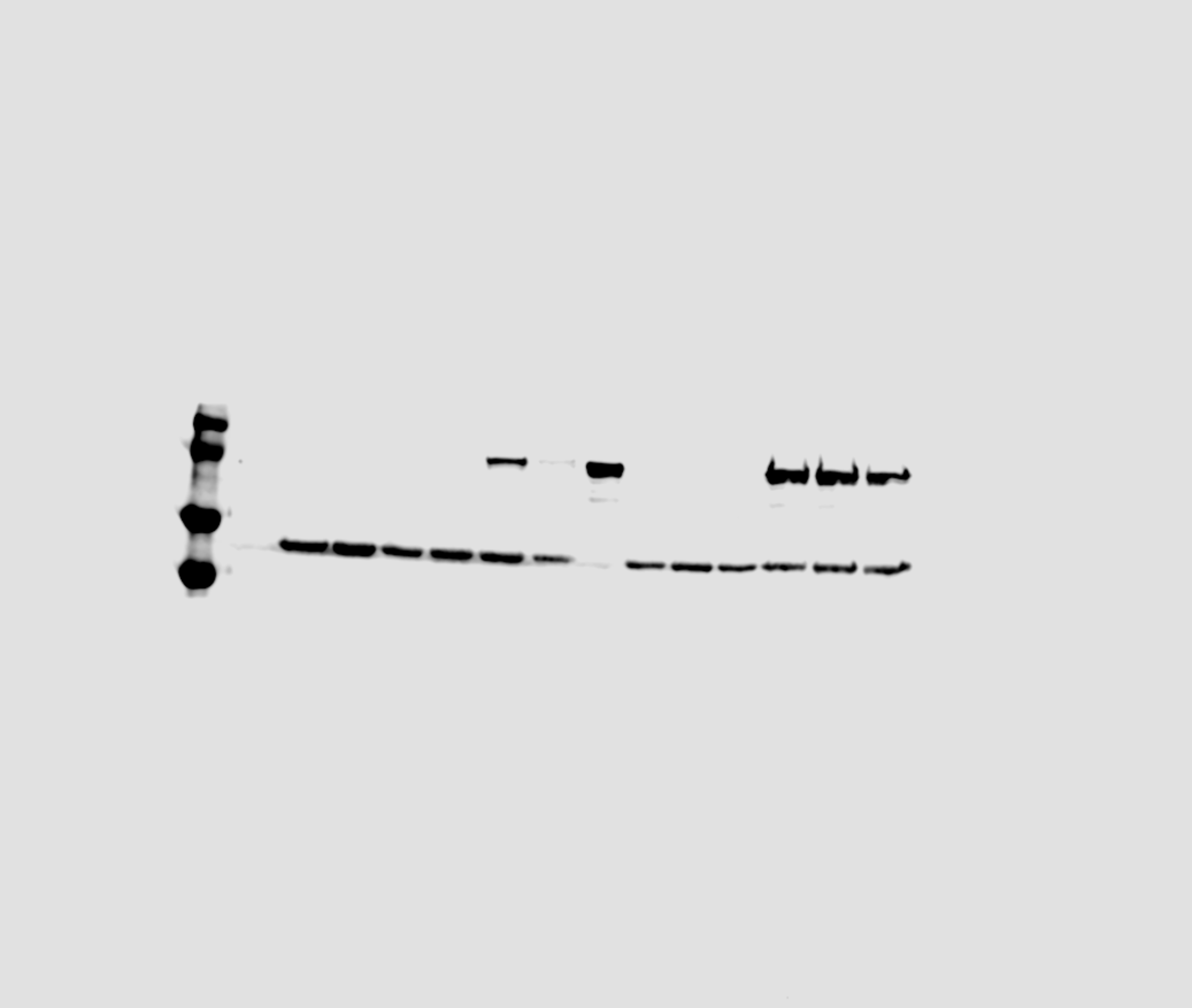

Supplement: Figure 4—source data 1. — KRAS probed with anti-GST antibody; NTs probed with anti-His antibody. [file elife-88836-fig4-data1.zip › Figure 4- source data 1/NT1_GST-KRAS_PD1_KRAS_3-2023.tif]

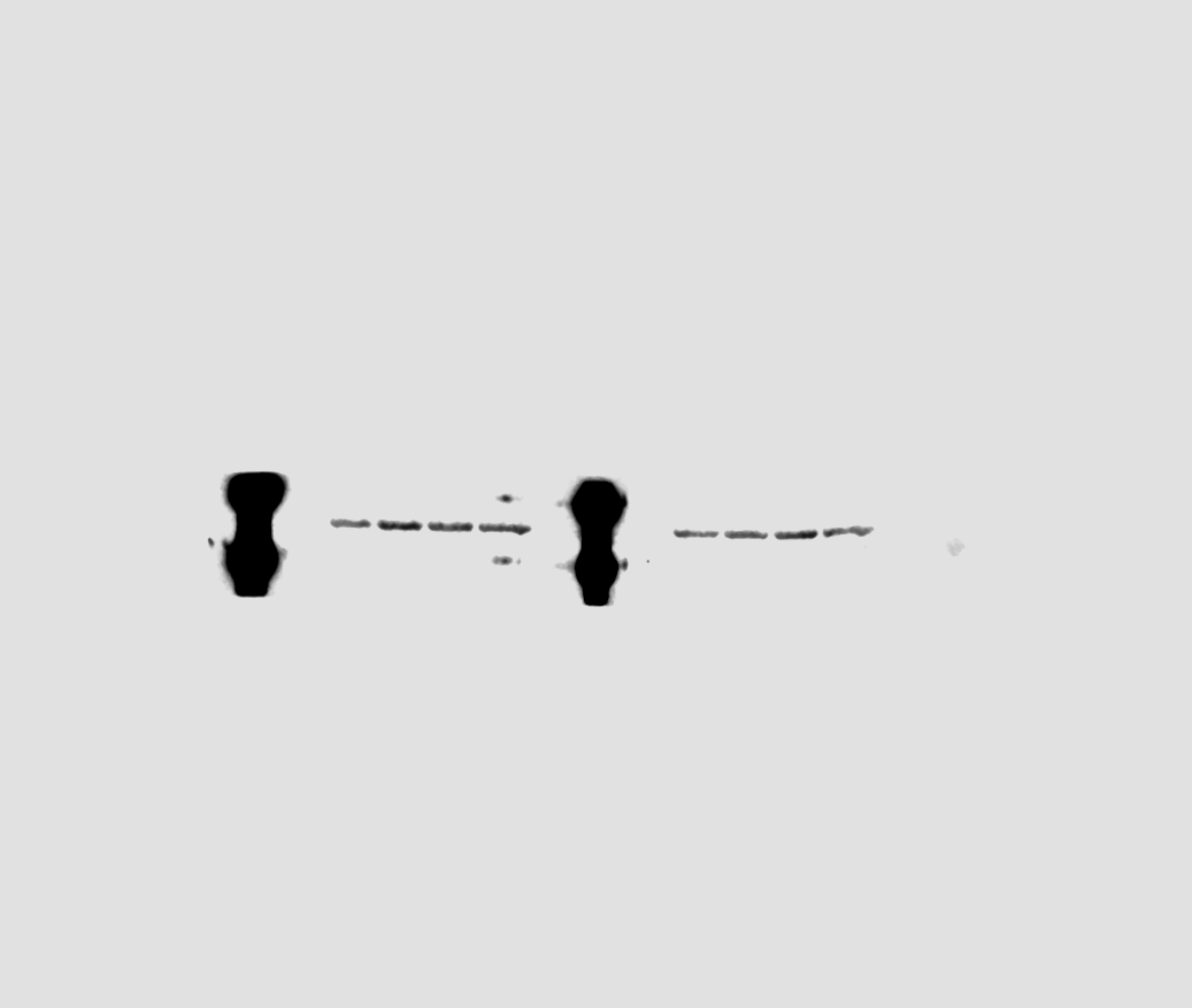

Supplement: Figure 4—source data 1. — KRAS probed with anti-GST antibody; NTs probed with anti-His antibody. [file elife-88836-fig4-data1.zip › Figure 4- source data 1/NT2_GST-KRAS_PDreruns_GST_3-2023.tif]

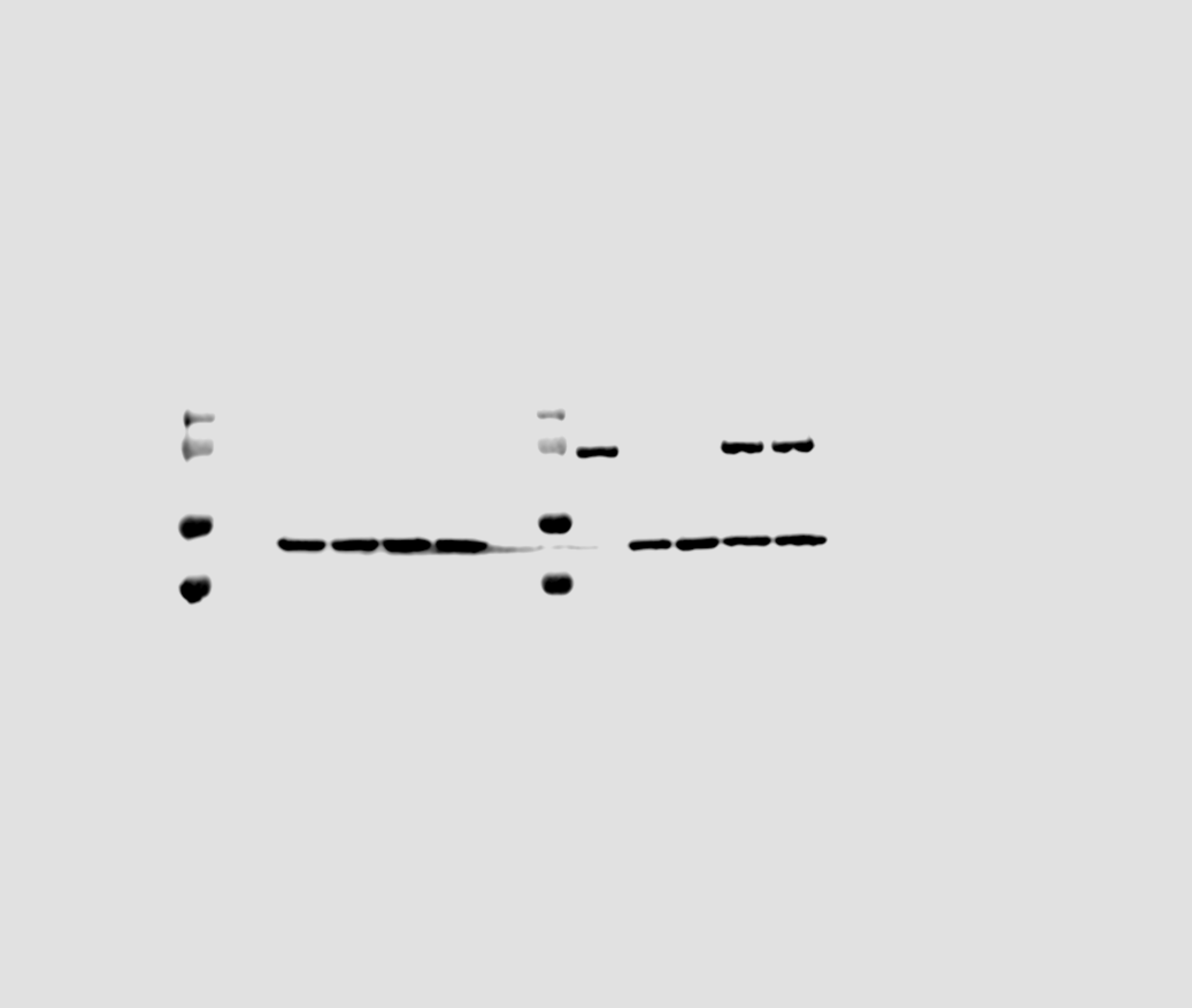

Supplement: Figure 4—source data 1. — KRAS probed with anti-GST antibody; NTs probed with anti-His antibody. [file elife-88836-fig4-data1.zip › Figure 4- source data 1/NT2_GST-KRAS_PDB_input_3-2023.tif]

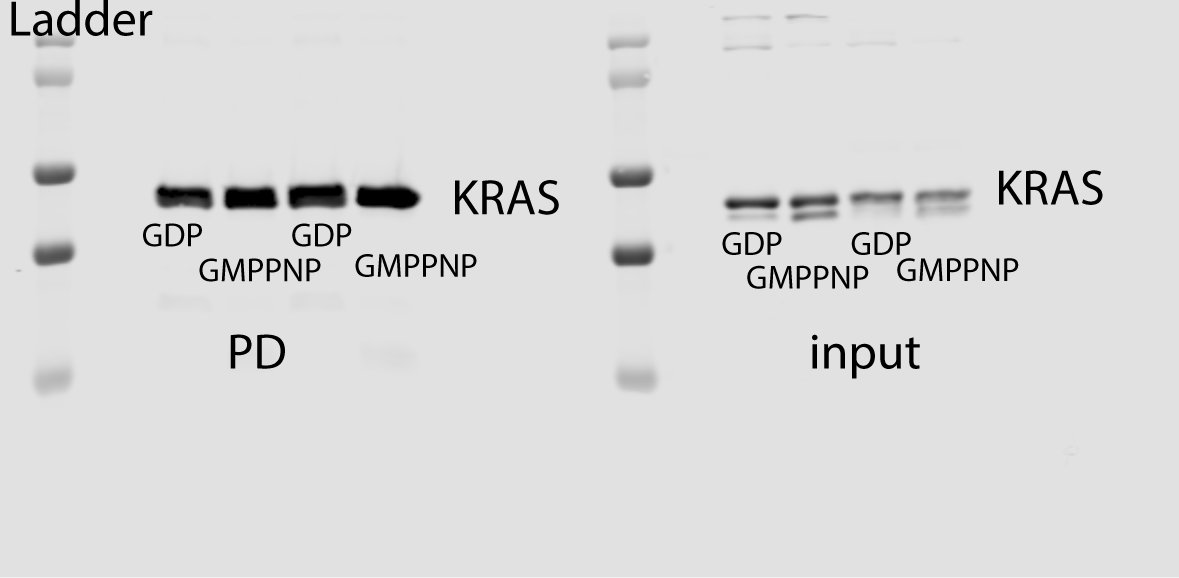

Supplement: Figure 4—source data 1. — KRAS probed with anti-GST antibody; NTs probed with anti-His antibody. [file elife-88836-fig4-data1.zip › Figure 4- source data 1/NT4_GST-KRAS_PD-gst-01.png]

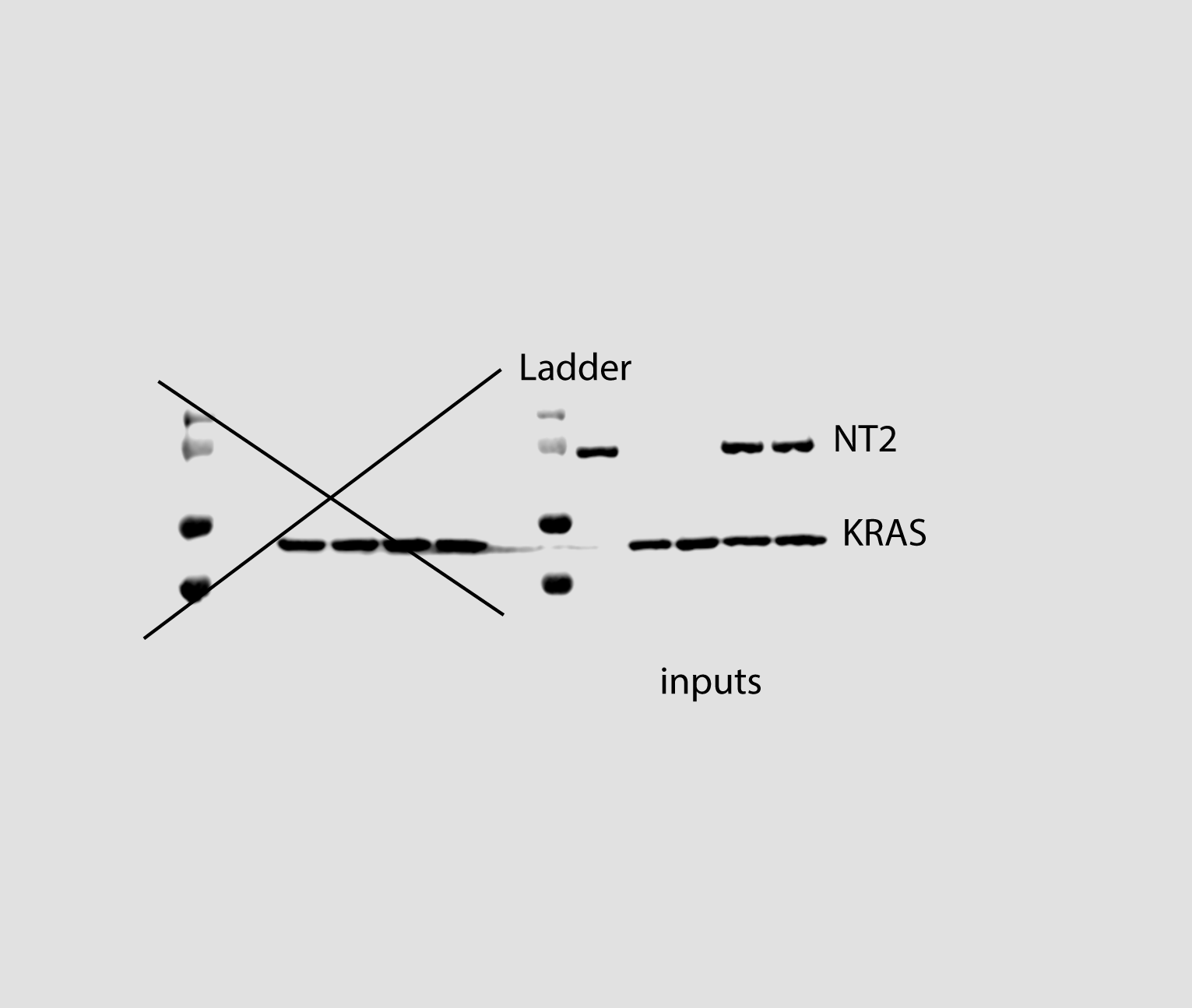

Supplement: Figure 4—source data 1. — KRAS probed with anti-GST antibody; NTs probed with anti-His antibody. [file elife-88836-fig4-data1.zip › Figure 4- source data 1/NT2_GST-KRAS_PDB_input_3-2023-01.png]

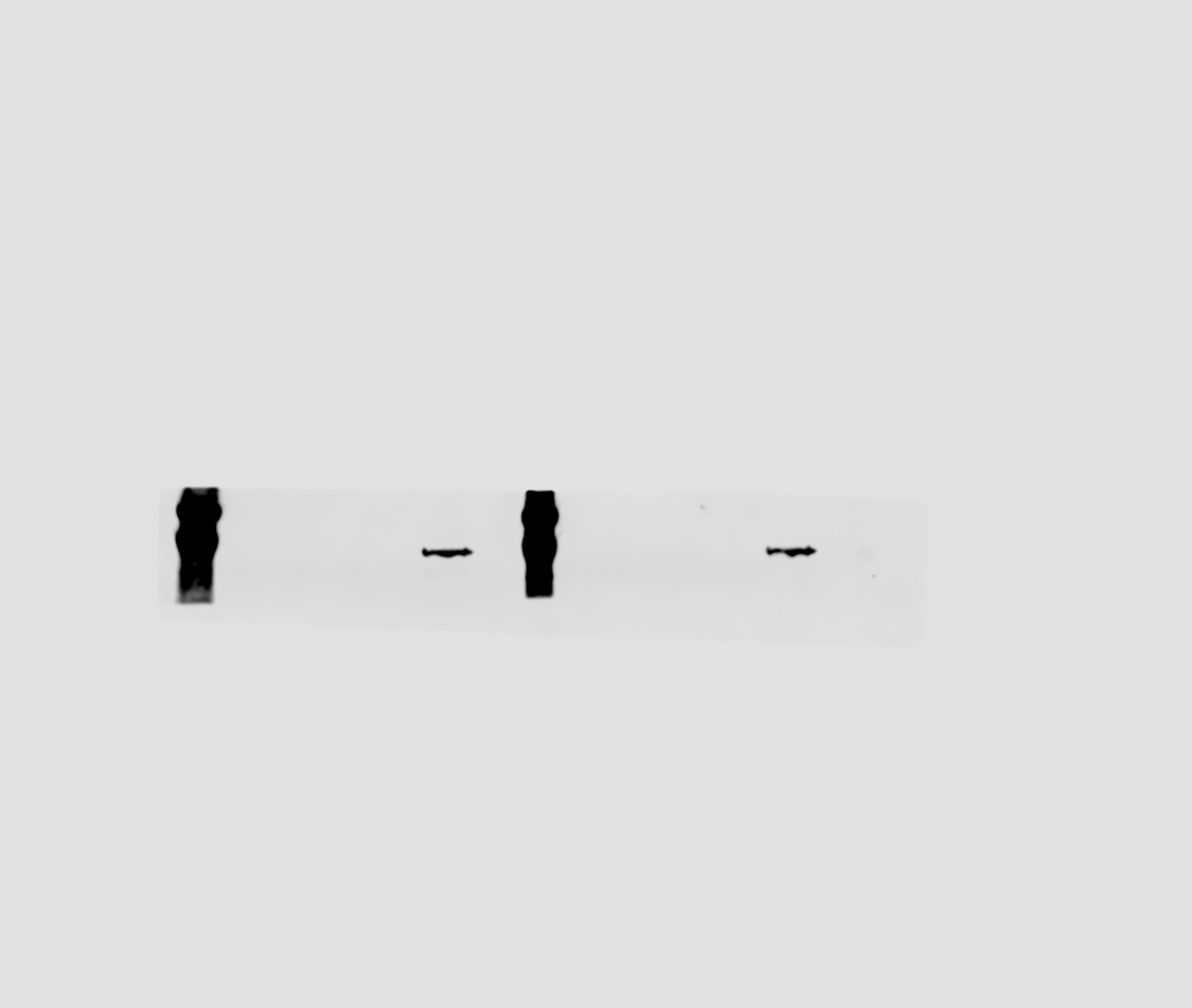

Supplement: Figure 4—source data 1. — KRAS probed with anti-GST antibody; NTs probed with anti-His antibody. [file elife-88836-fig4-data1.zip › Figure 4- source data 1/NT2_GST-KRAS_PDreruns_his_3-2023.tif]

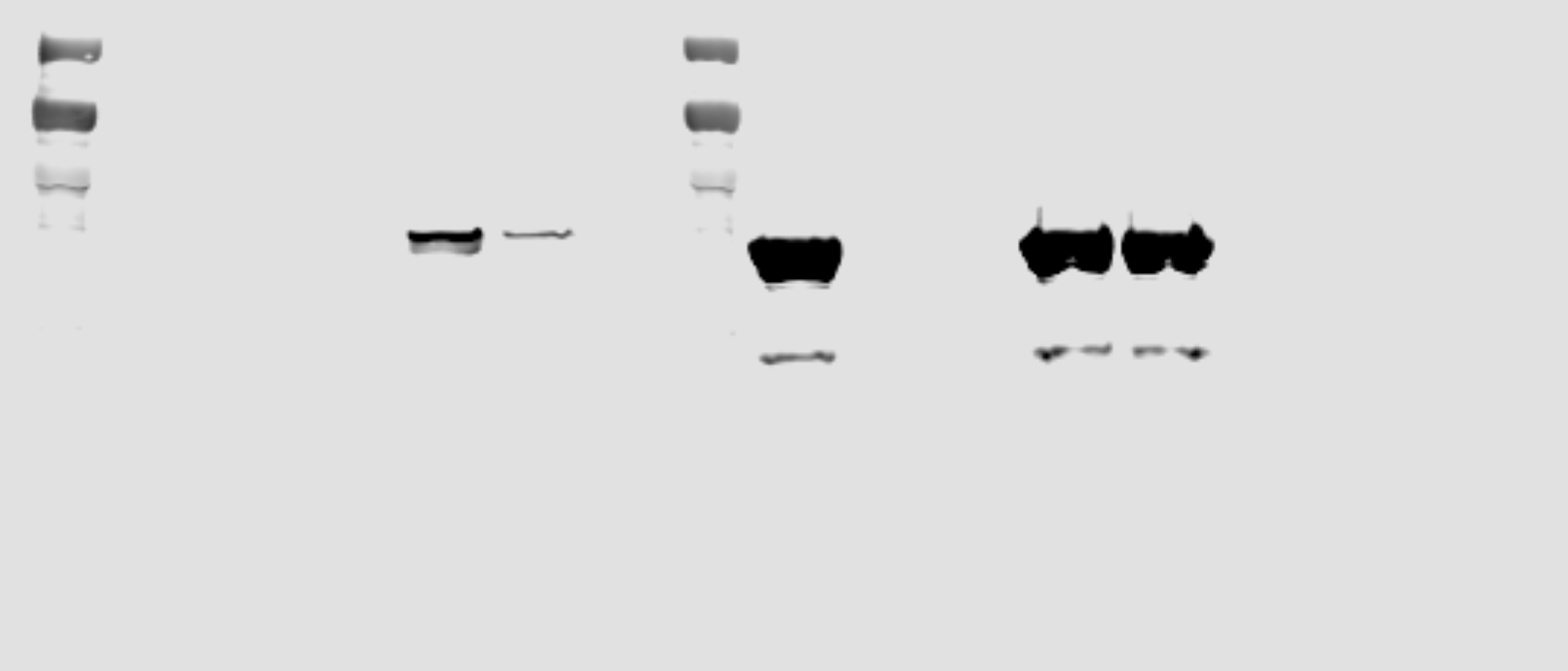

Supplement: Figure 4—source data 1. — KRAS probed with anti-GST antibody; NTs probed with anti-His antibody. [file elife-88836-fig4-data1.zip › Figure 4- source data 1/NT3_GST-KRAS_INPUT (right).tif]

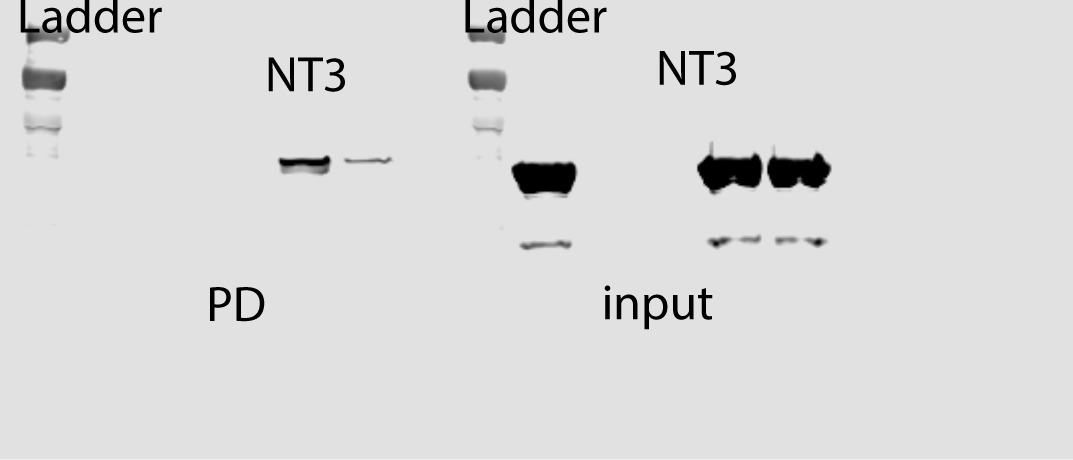

Supplement: Figure 4—source data 1. — KRAS probed with anti-GST antibody; NTs probed with anti-His antibody. [file elife-88836-fig4-data1.zip › Figure 4- source data 1/NT3_GST-KRAS_INPUT (right)-01.png]

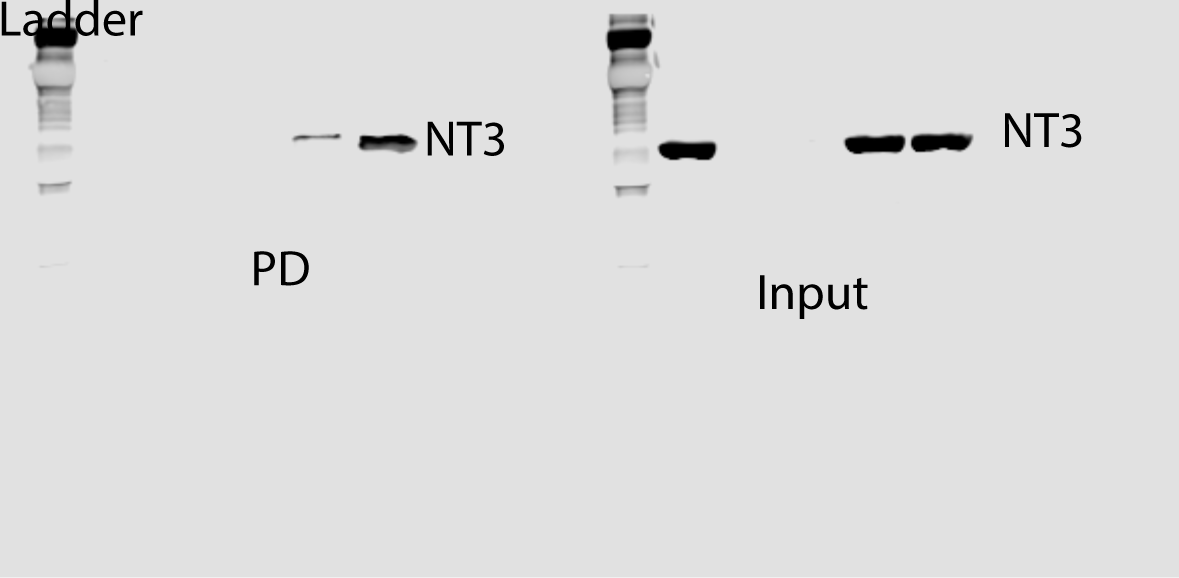

Supplement: Figure 4—source data 1. — KRAS probed with anti-GST antibody; NTs probed with anti-His antibody. [file elife-88836-fig4-data1.zip › Figure 4- source data 1/NT4_GST-KRAS_PD-NT4 left_INPUT-NT4 right-01.png]

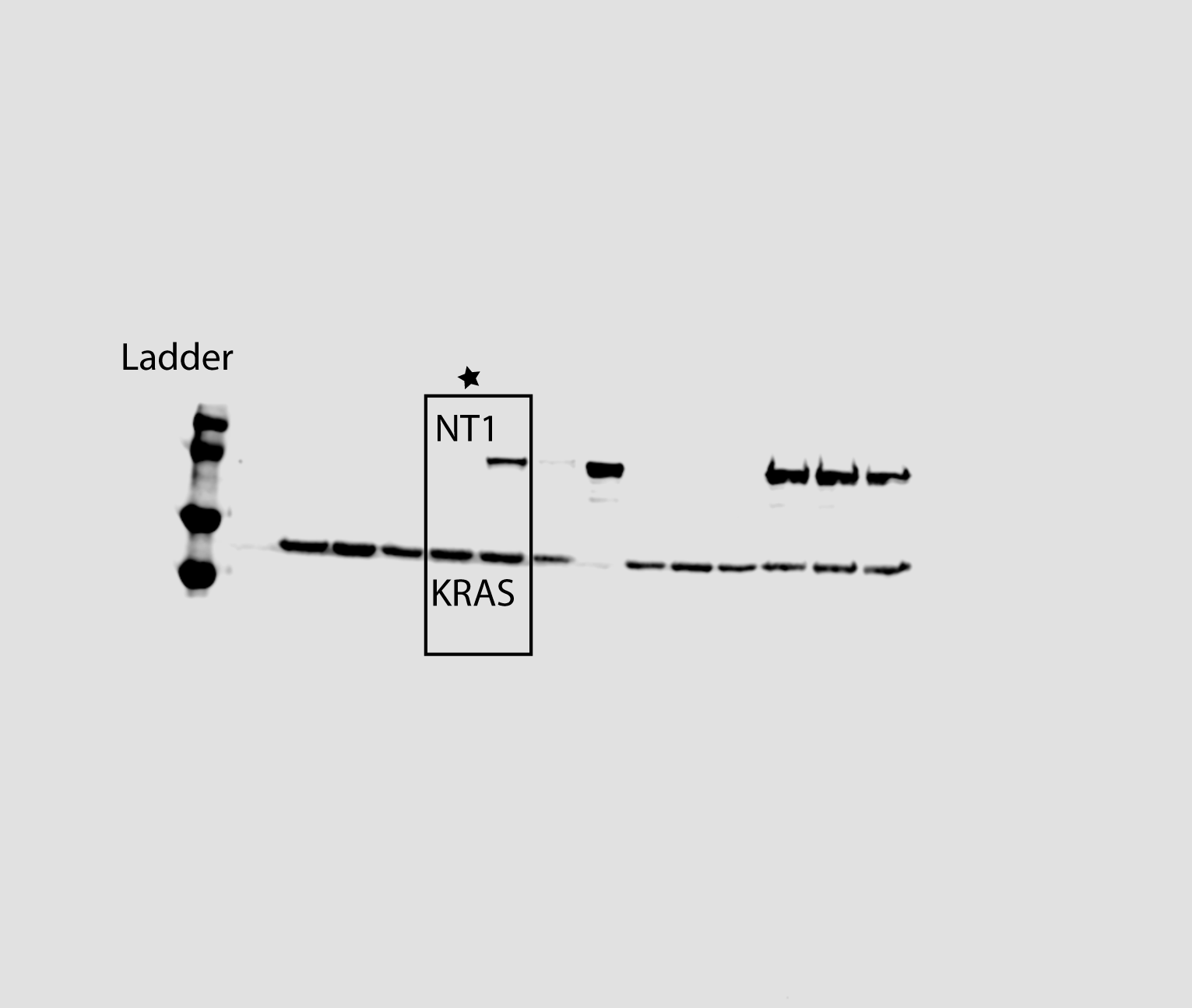

Supplement: Figure 4—source data 1. — KRAS probed with anti-GST antibody; NTs probed with anti-His antibody. [file elife-88836-fig4-data1.zip › Figure 4- source data 1/NT1_GST-KRAS_PD1_KRAS_3-2023-01.png]

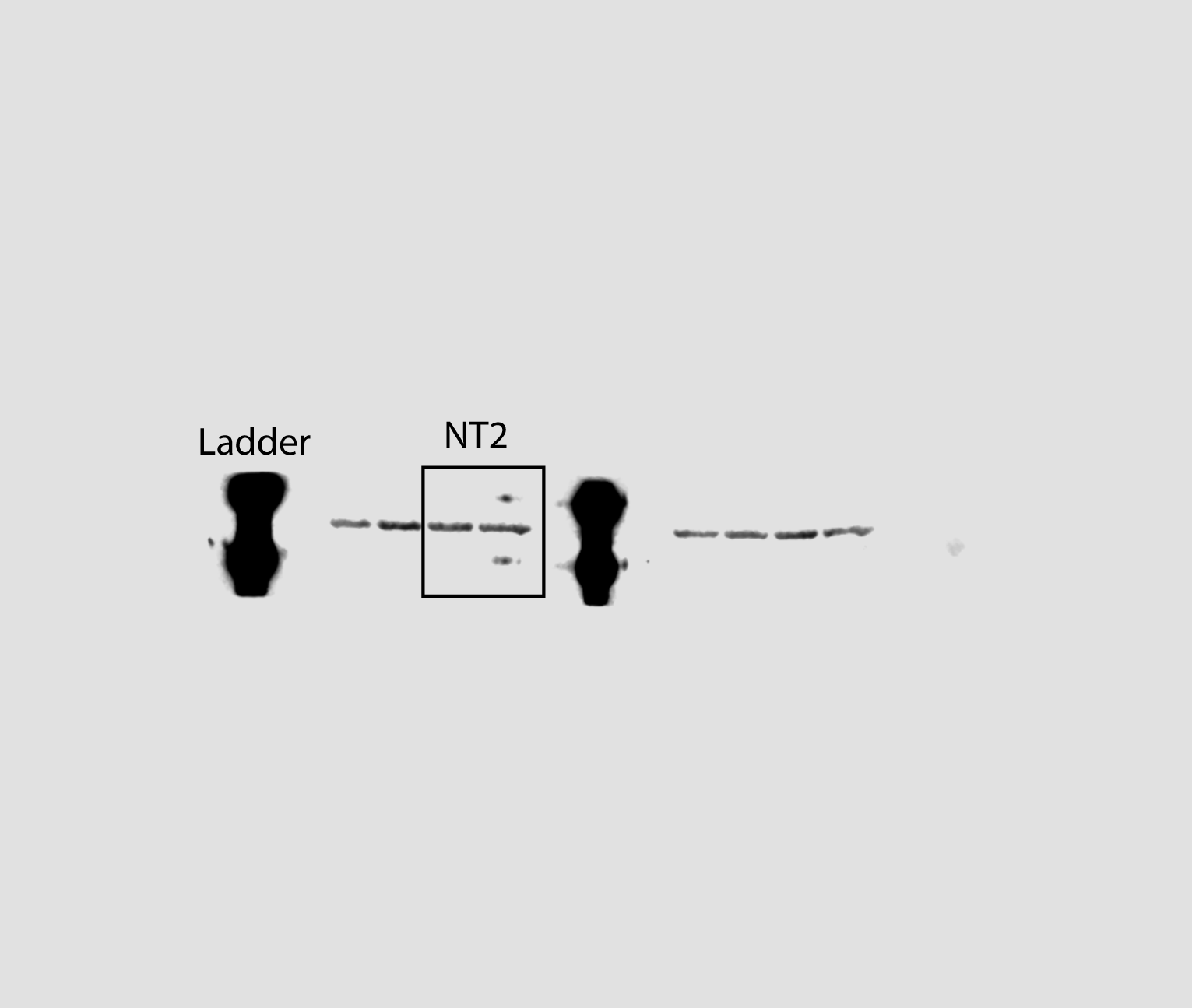

Supplement: Figure 4—source data 1. — KRAS probed with anti-GST antibody; NTs probed with anti-His antibody. [file elife-88836-fig4-data1.zip › Figure 4- source data 1/NT2_GST-KRAS_PDreruns_GST_3-2023-01.png]

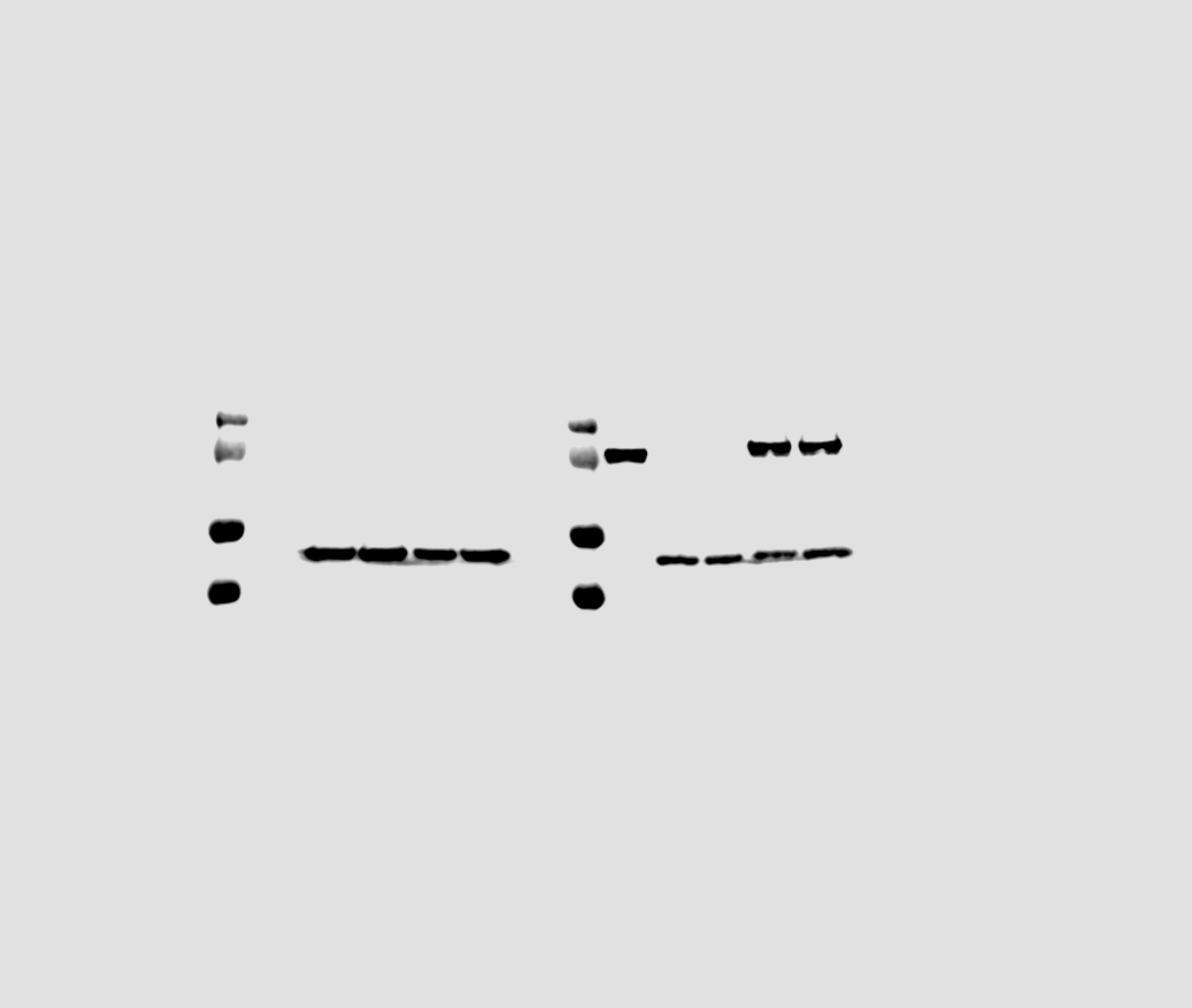

Supplement: Figure 4—source data 1. — KRAS probed with anti-GST antibody; NTs probed with anti-His antibody. [file elife-88836-fig4-data1.zip › Figure 4- source data 1/NT1_GST-KRAS_PD1_input_3-2023.tif]

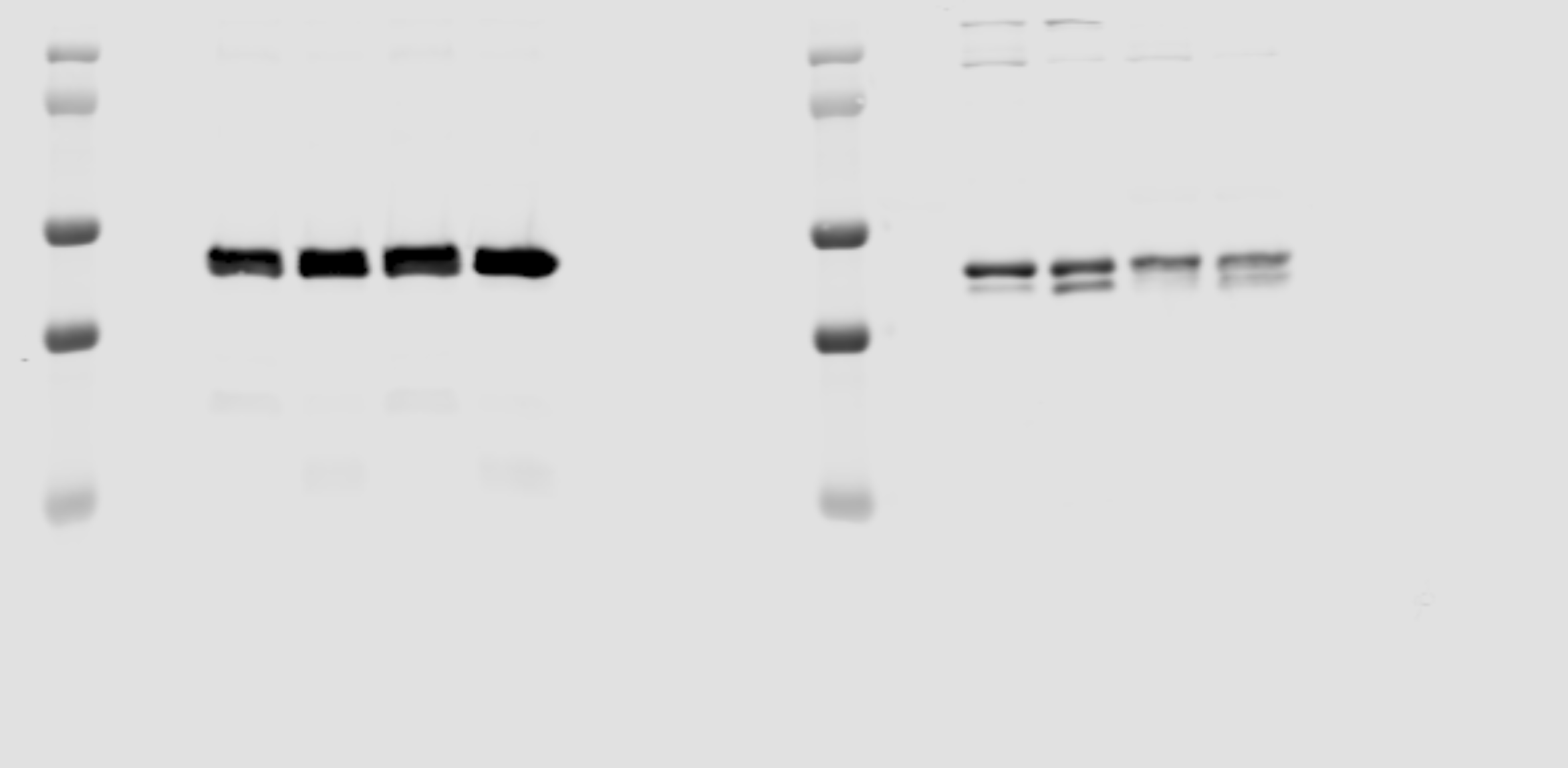

Supplement: Figure 4—source data 1. — KRAS probed with anti-GST antibody; NTs probed with anti-His antibody. [file elife-88836-fig4-data1.zip › Figure 4- source data 1/NT4_GST-KRAS_PD-gst.tif]

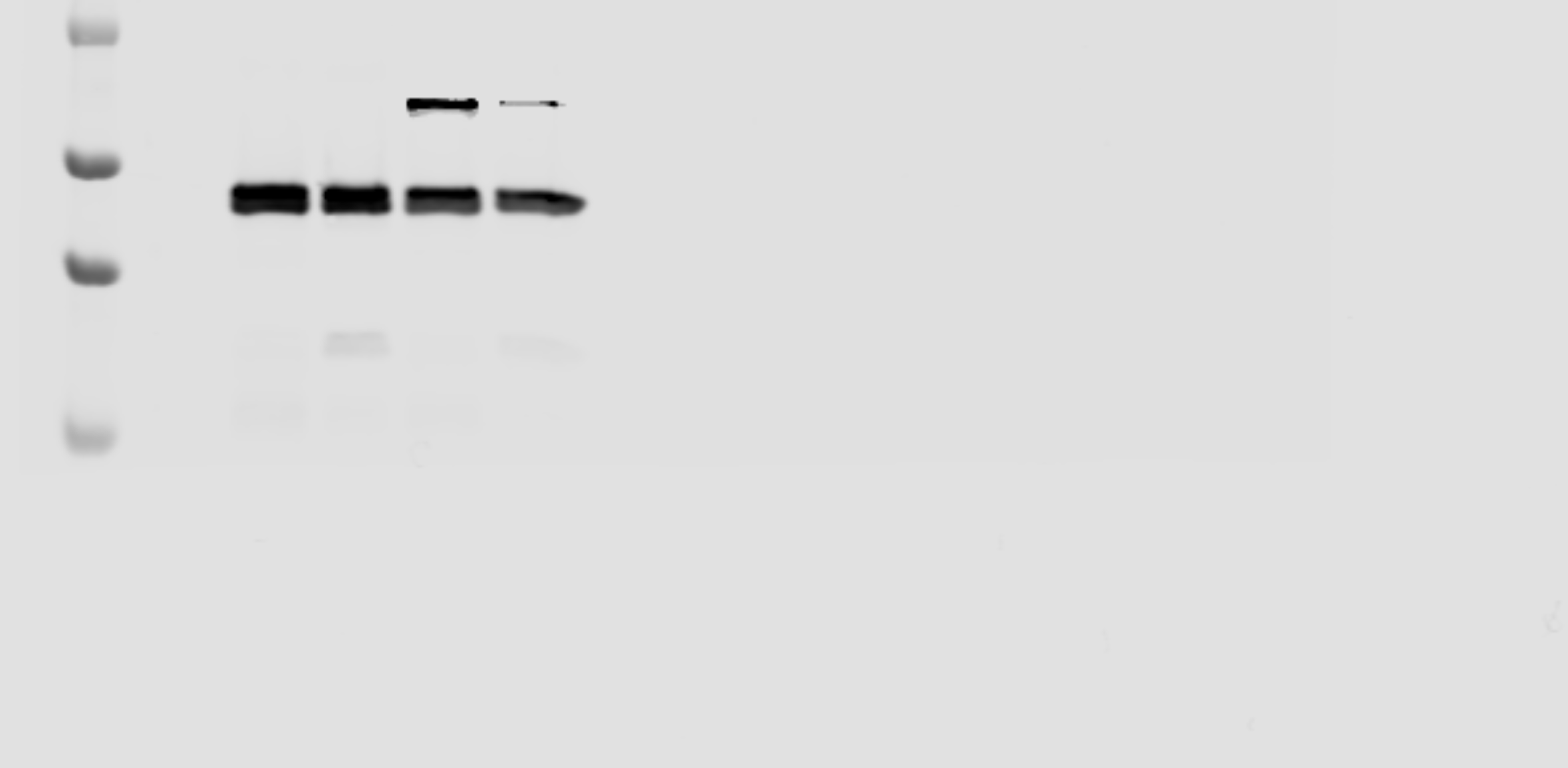

Supplement: Figure 4—source data 1. — KRAS probed with anti-GST antibody; NTs probed with anti-His antibody. [file elife-88836-fig4-data1.zip › Figure 4- source data 1/NT3_GST-KRAS_PD.tif]

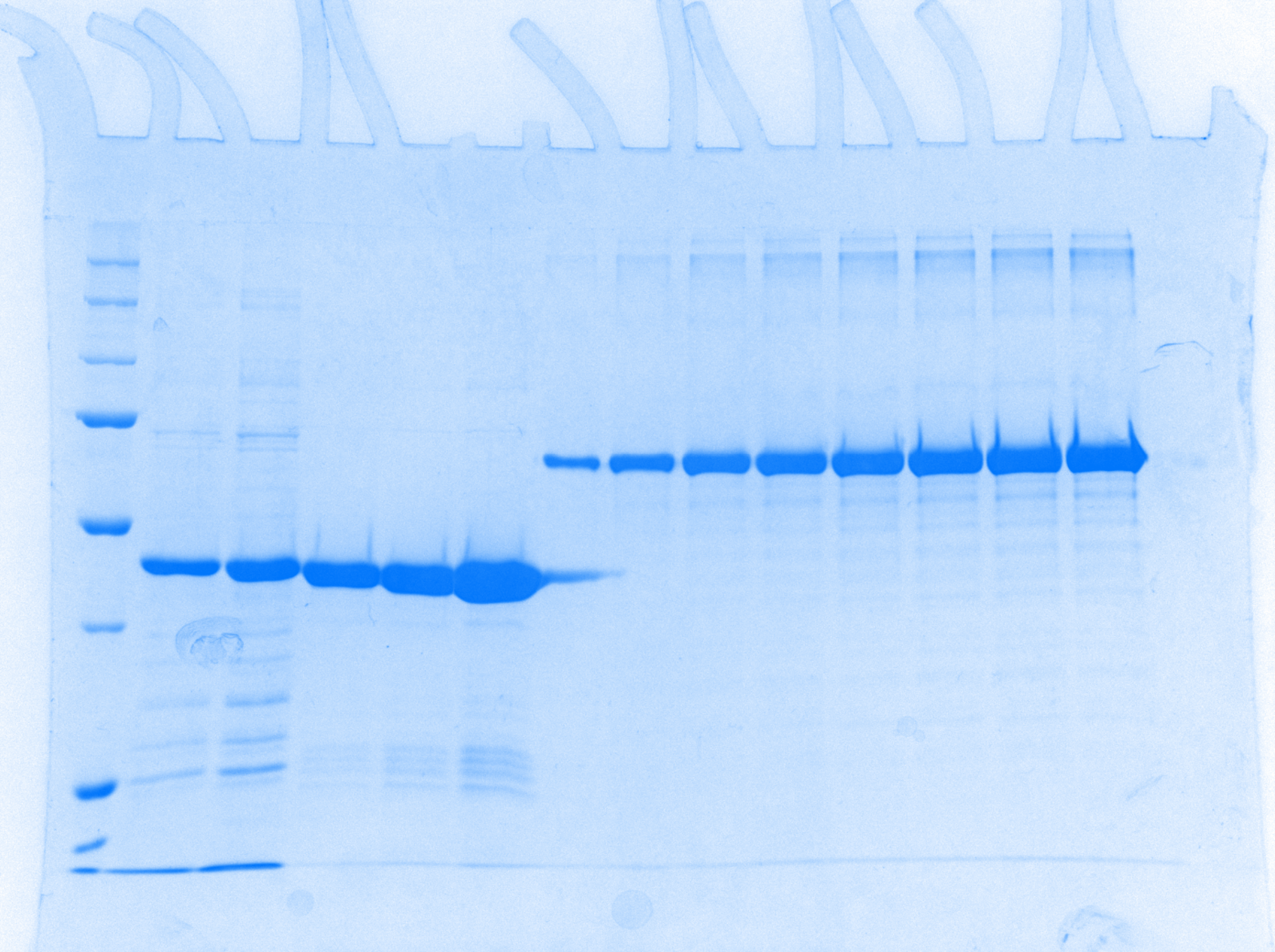

Supplement: Figure 4—figure supplement 1—source data 1. [file elife-88836-fig4-figsupp1-data1.zip › Figure 4- figure supplement 1- source data 1/GST KRAS.tif]

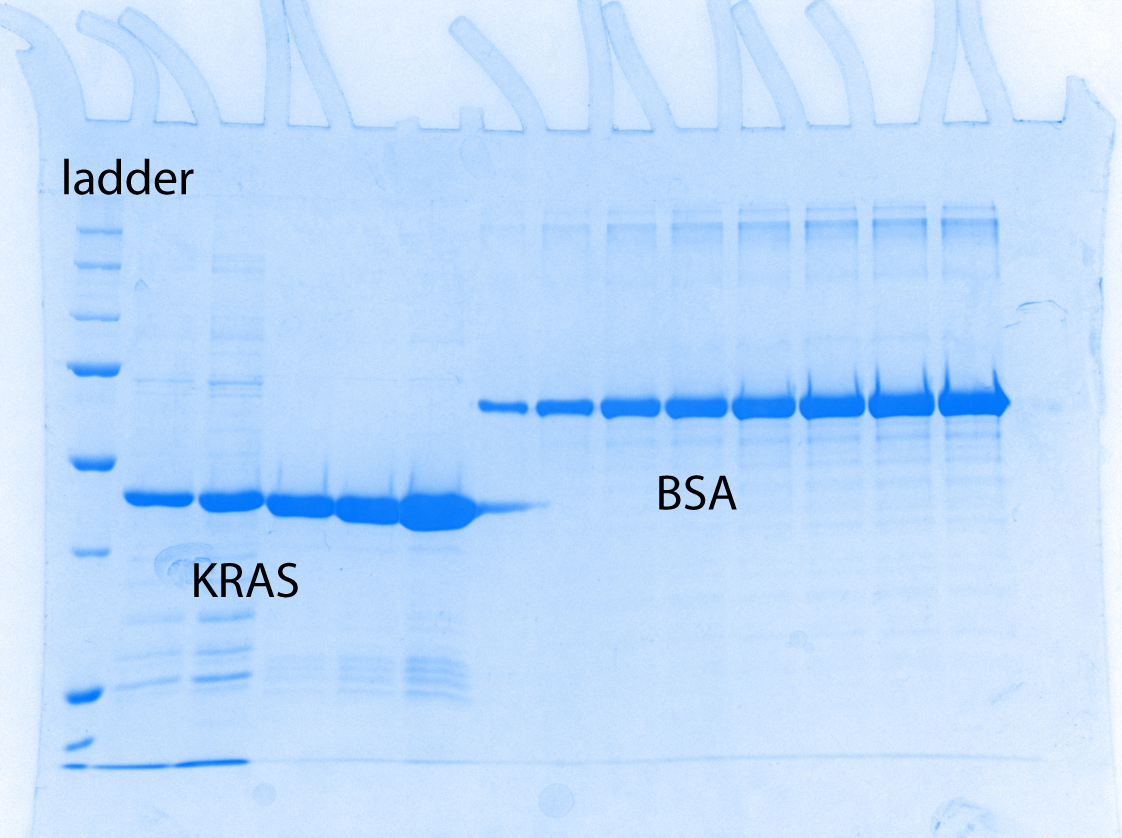

Supplement: Figure 4—figure supplement 1—source data 1. [file elife-88836-fig4-figsupp1-data1.zip › Figure 4- figure supplement 1- source data 1/GST KRAS-01.png]

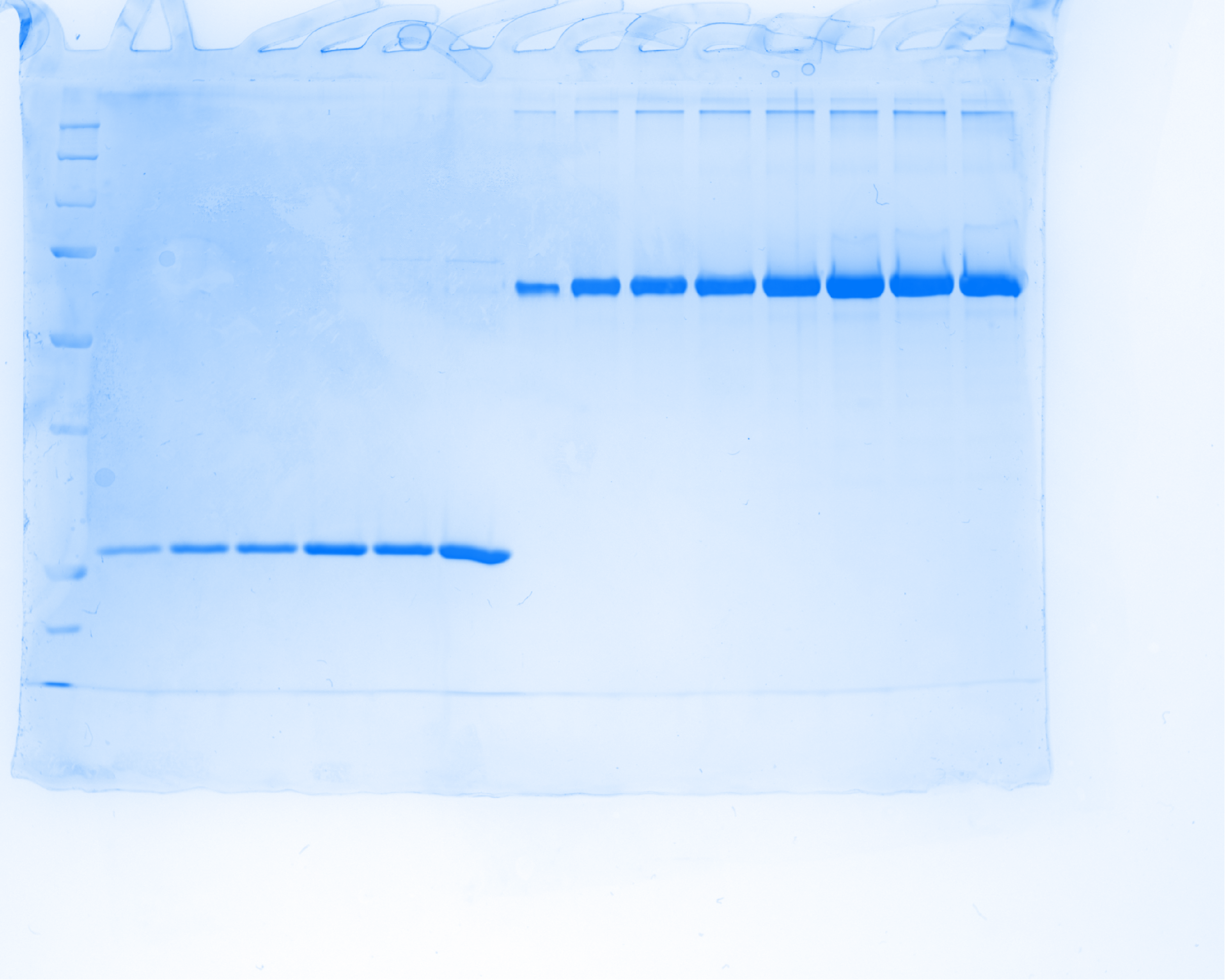

Supplement: Figure 4—figure supplement 2—source data 1. [file elife-88836-fig4-figsupp2-data1.zip › Figure 4- figure supplement 2- source data 1/GSTquant.tif]

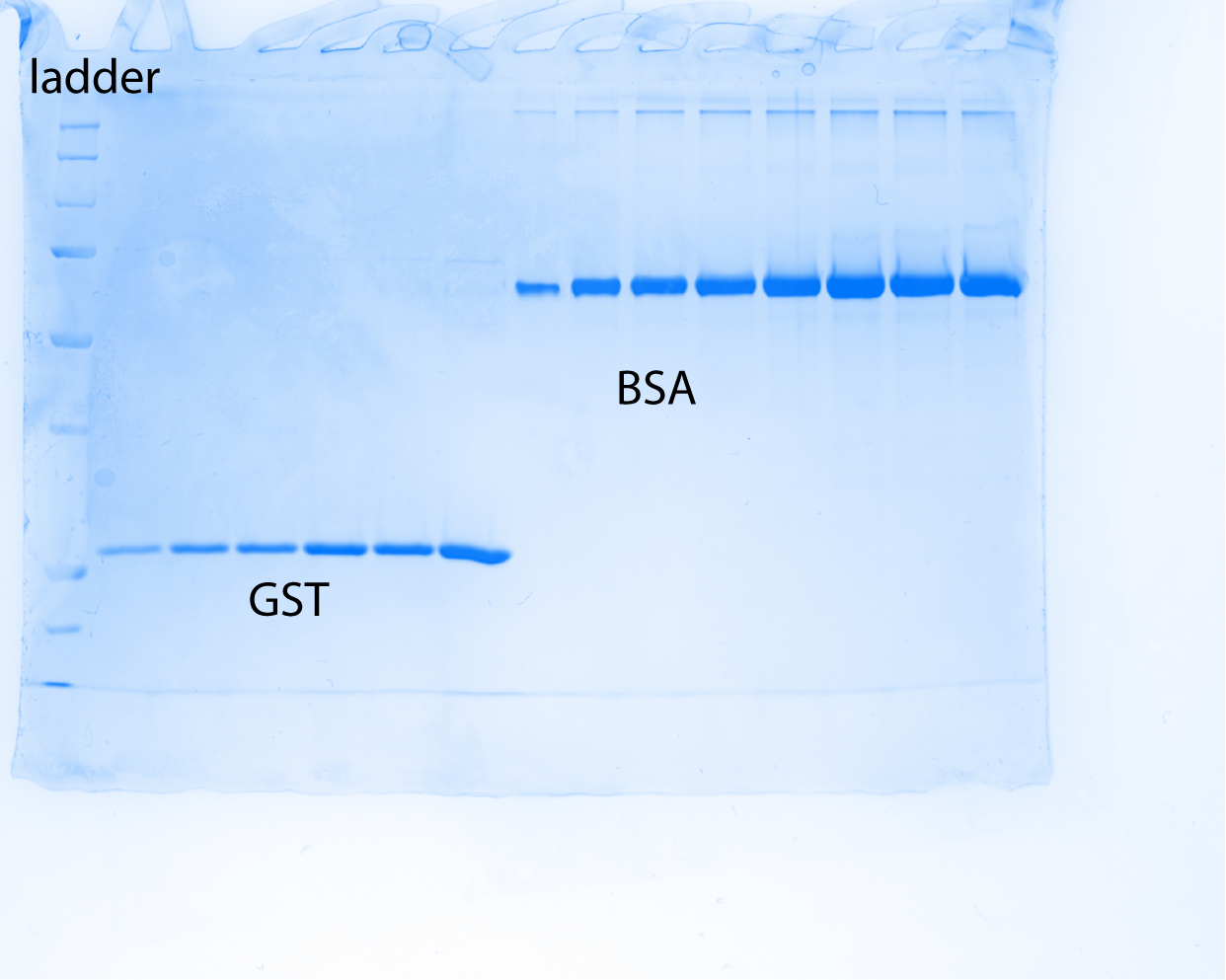

Supplement: Figure 4—figure supplement 2—source data 1. [file elife-88836-fig4-figsupp2-data1.zip › Figure 4- figure supplement 2- source data 1/GSTquant-01.png]

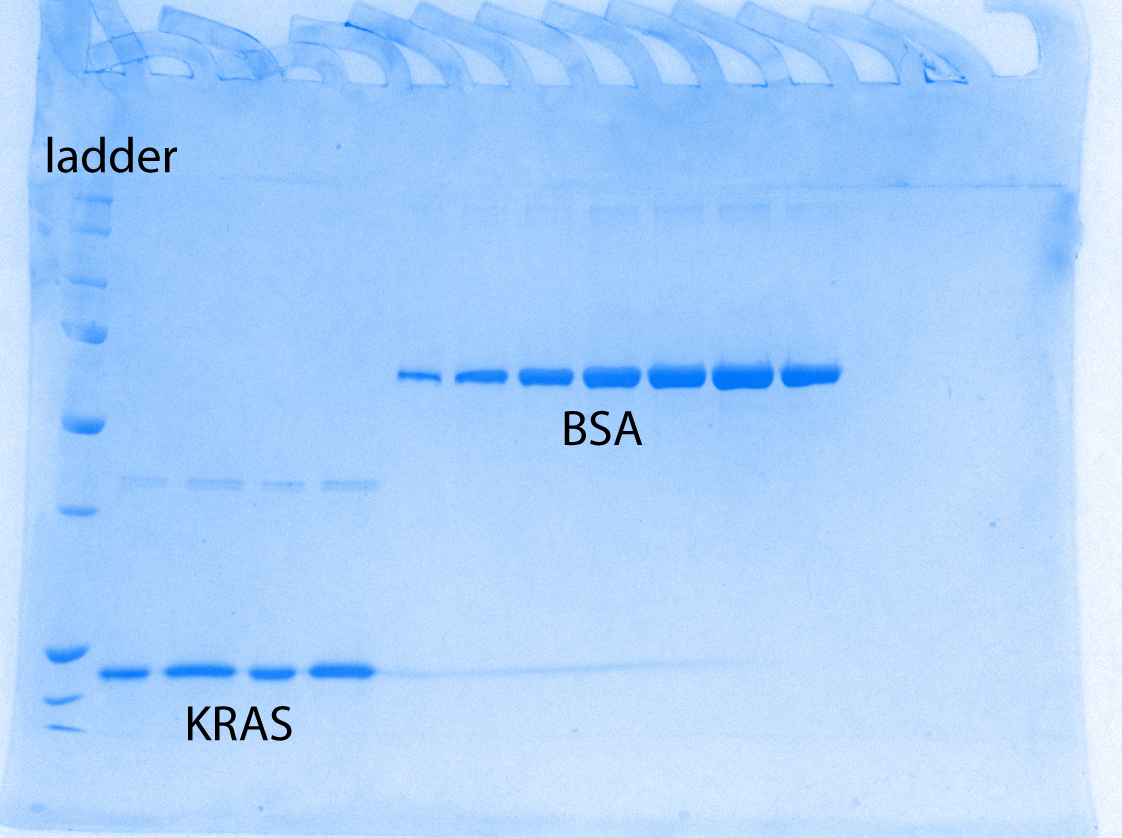

Supplement: Figure 4—figure supplement 3—source data 1. — KRAS probed with RAS (E8N8L) XP Rabbit mAb (Cell Signaling #67648); NTs probed with anti-His antibody. [file elife-88836-fig4-figsupp3-data1.zip › Figure 4- figure supplement 3- source data 1/KRAS quant-01.png]

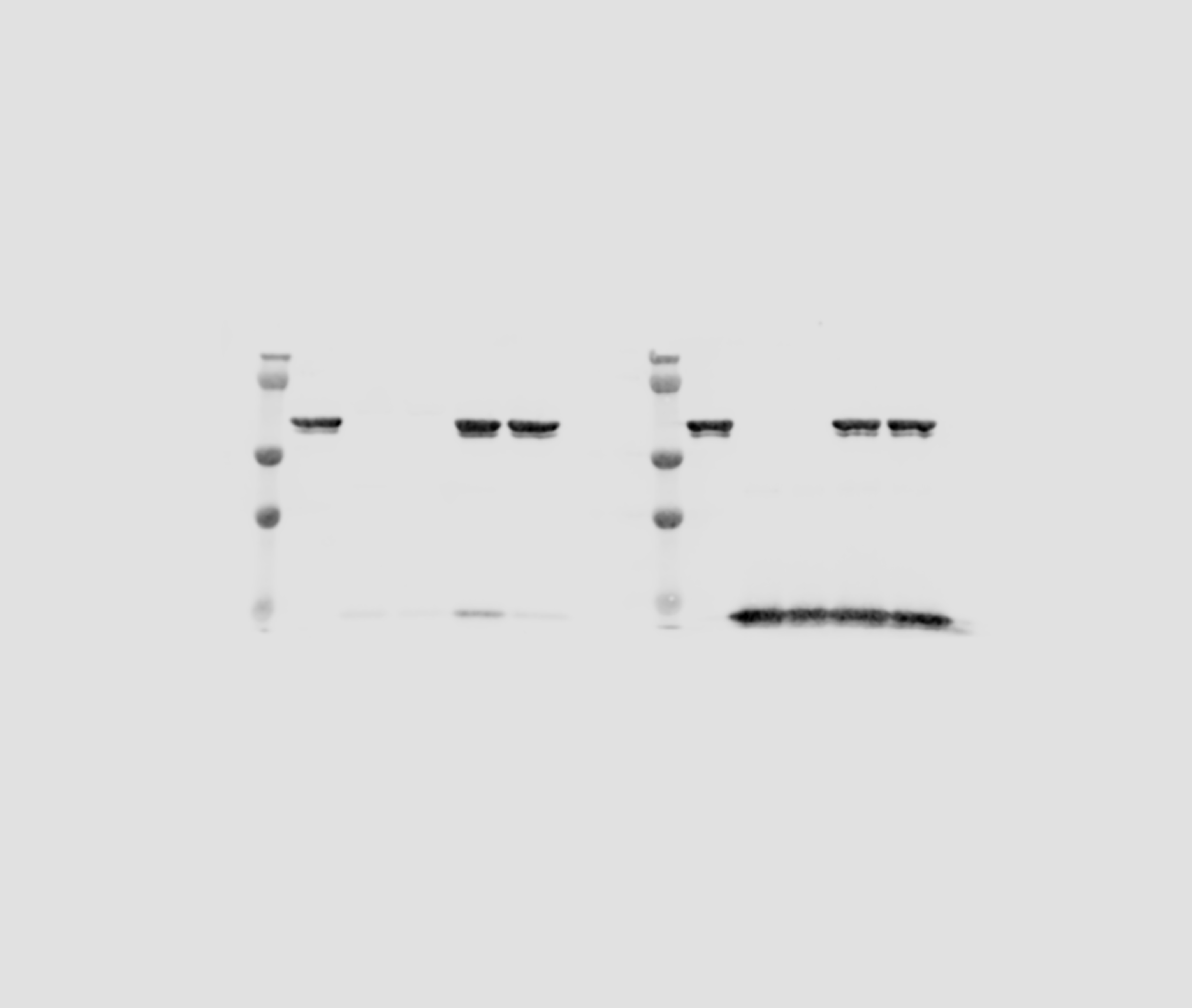

Supplement: Figure 4—figure supplement 3—source data 1. — KRAS probed with RAS (E8N8L) XP Rabbit mAb (Cell Signaling #67648); NTs probed with anti-His antibody. [file elife-88836-fig4-figsupp3-data1.zip › Figure 4- figure supplement 3- source data 1/NT3_K2_PD-left_inp-right.tif]

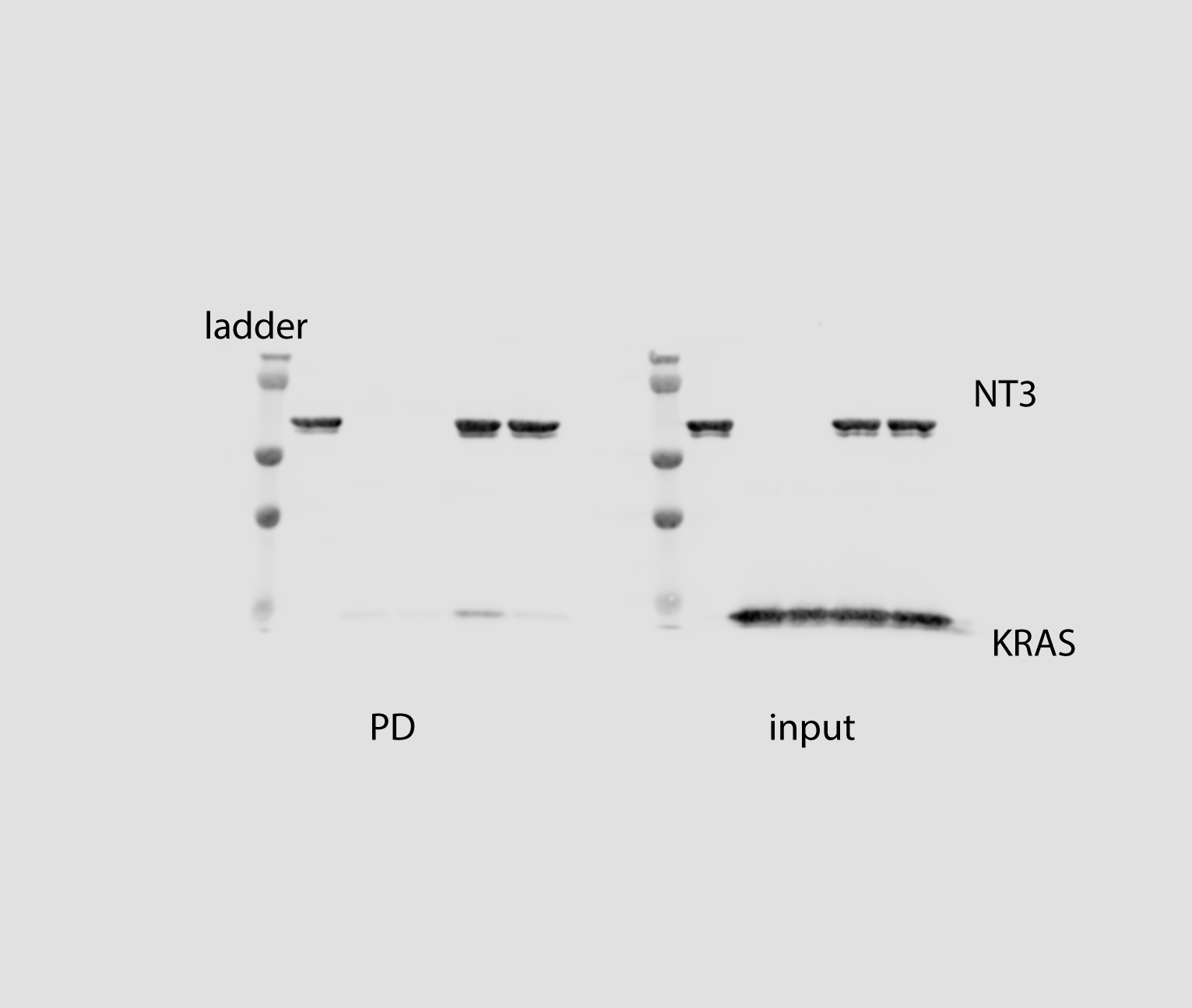

Supplement: Figure 4—figure supplement 3—source data 1. — KRAS probed with RAS (E8N8L) XP Rabbit mAb (Cell Signaling #67648); NTs probed with anti-His antibody. [file elife-88836-fig4-figsupp3-data1.zip › Figure 4- figure supplement 3- source data 1/NT3_K2_PD-left_inp-right-01.png]

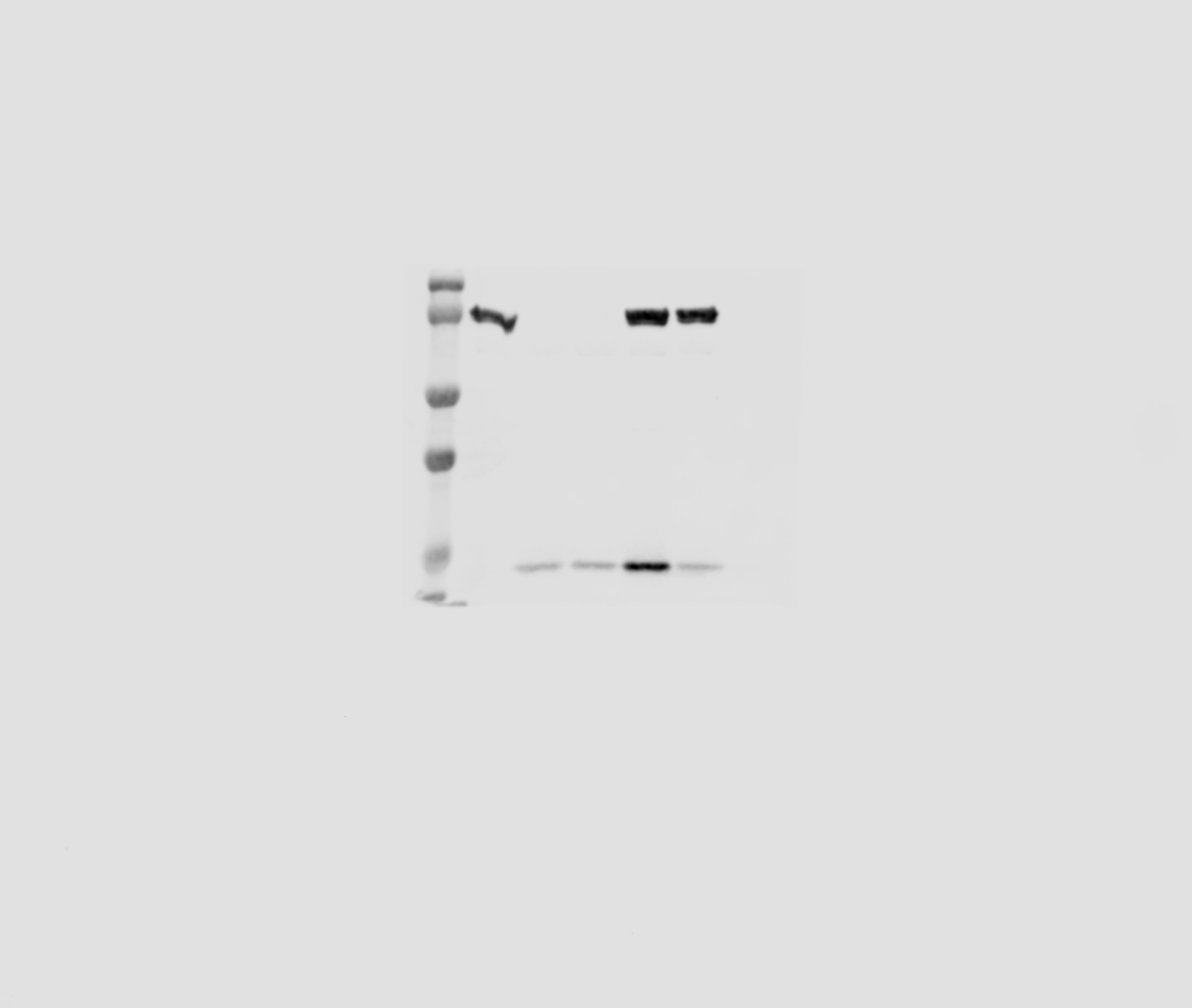

Supplement: Figure 4—figure supplement 3—source data 1. — KRAS probed with RAS (E8N8L) XP Rabbit mAb (Cell Signaling #67648); NTs probed with anti-His antibody. [file elife-88836-fig4-figsupp3-data1.zip › Figure 4- figure supplement 3- source data 1/NT1_K2_PD.tif]

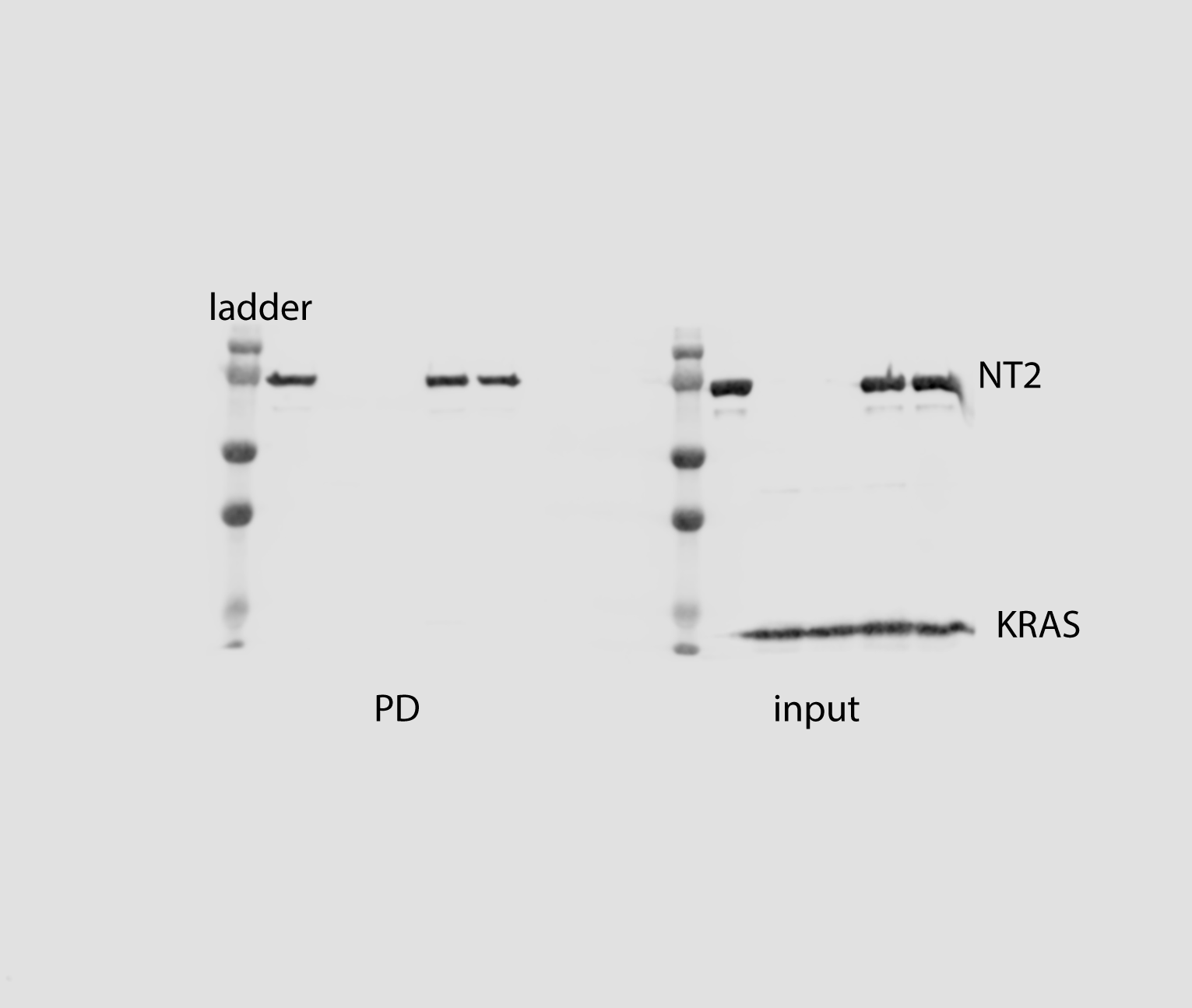

Supplement: Figure 4—figure supplement 3—source data 1. — KRAS probed with RAS (E8N8L) XP Rabbit mAb (Cell Signaling #67648); NTs probed with anti-His antibody. [file elife-88836-fig4-figsupp3-data1.zip › Figure 4- figure supplement 3- source data 1/NT2_K2_inp-right-01.png]

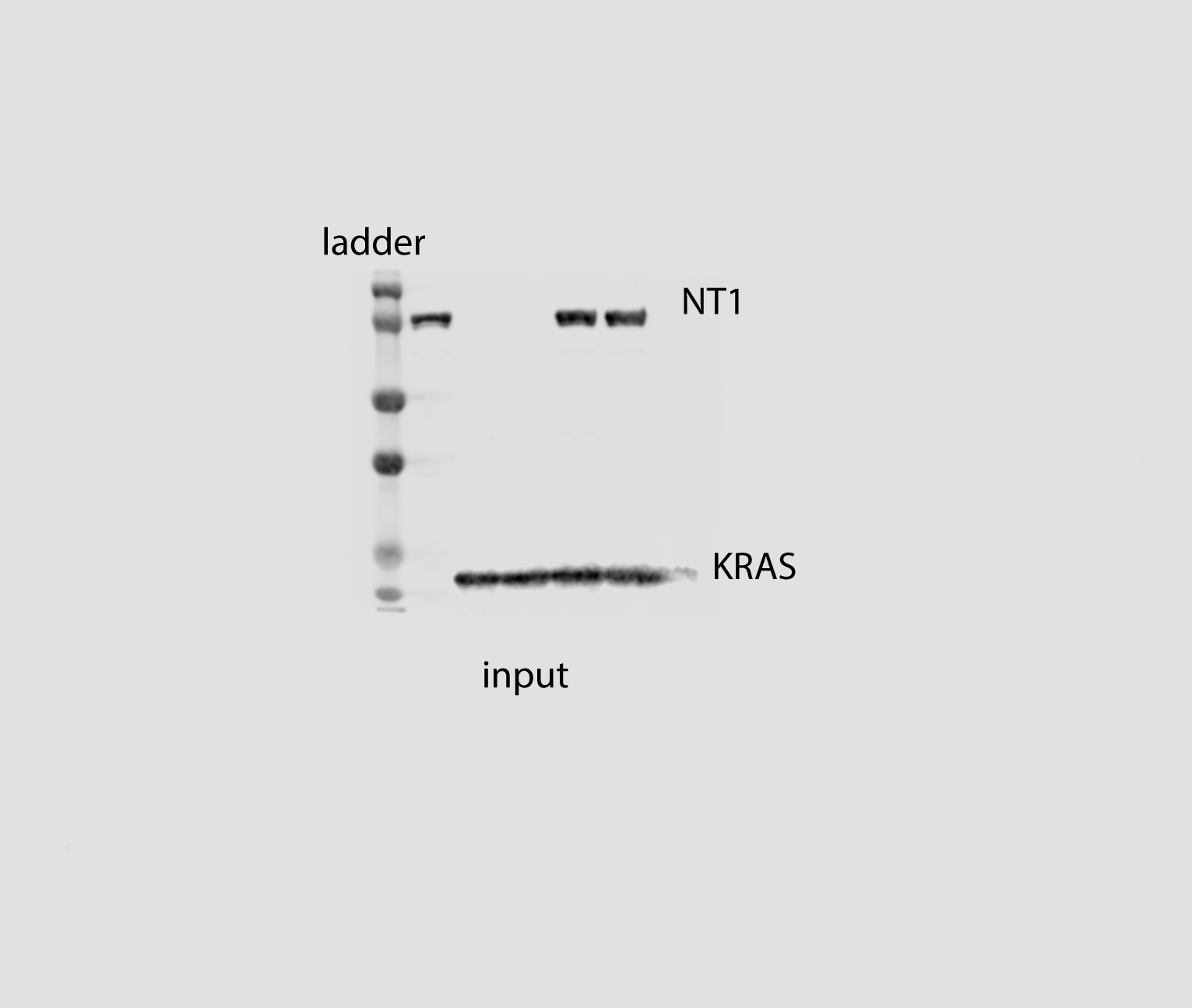

Supplement: Figure 4—figure supplement 3—source data 1. — KRAS probed with RAS (E8N8L) XP Rabbit mAb (Cell Signaling #67648); NTs probed with anti-His antibody. [file elife-88836-fig4-figsupp3-data1.zip › Figure 4- figure supplement 3- source data 1/NT1_K2_inp-01.png]

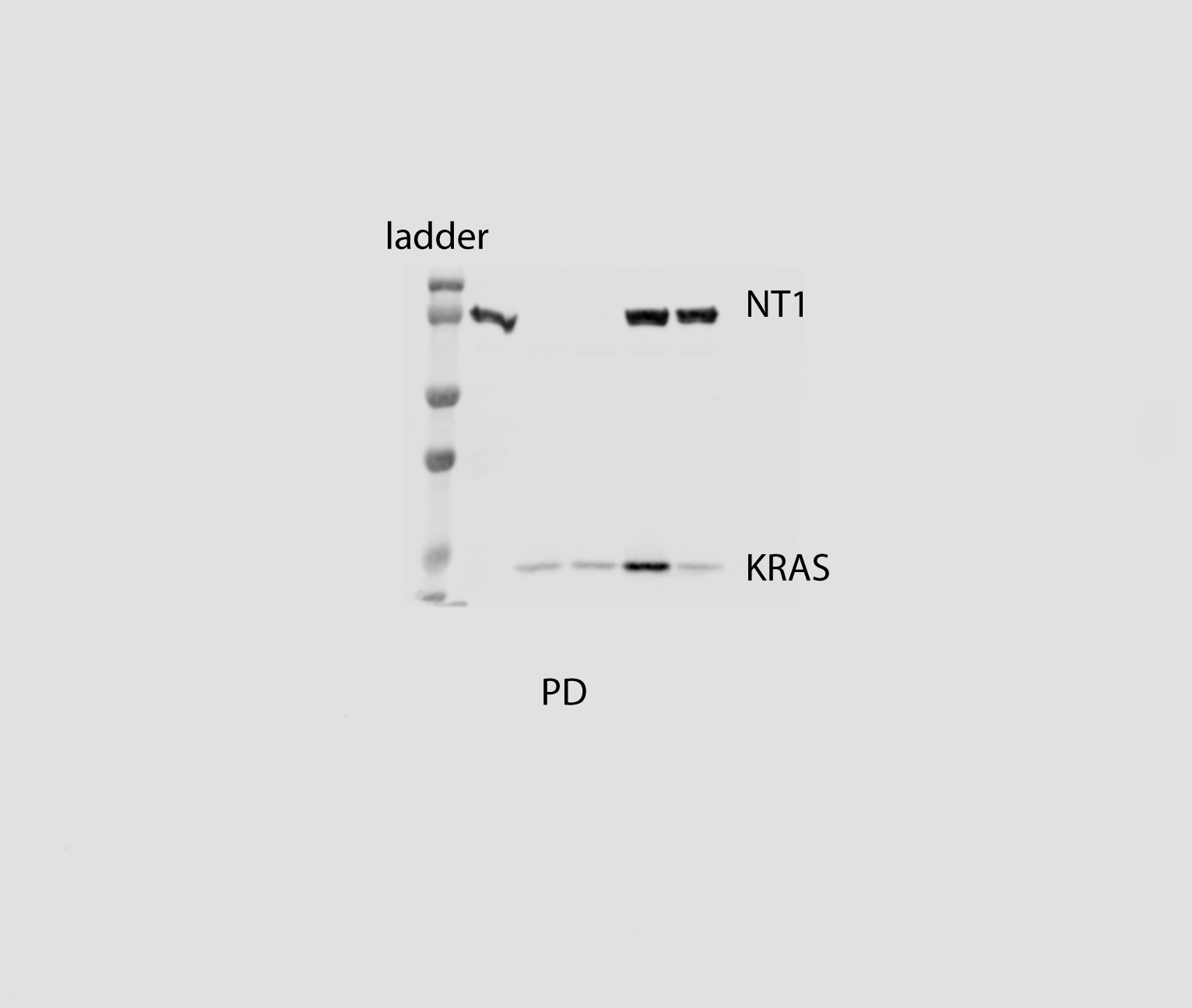

Supplement: Figure 4—figure supplement 3—source data 1. — KRAS probed with RAS (E8N8L) XP Rabbit mAb (Cell Signaling #67648); NTs probed with anti-His antibody. [file elife-88836-fig4-figsupp3-data1.zip › Figure 4- figure supplement 3- source data 1/NT1_K2_PD-01.png]

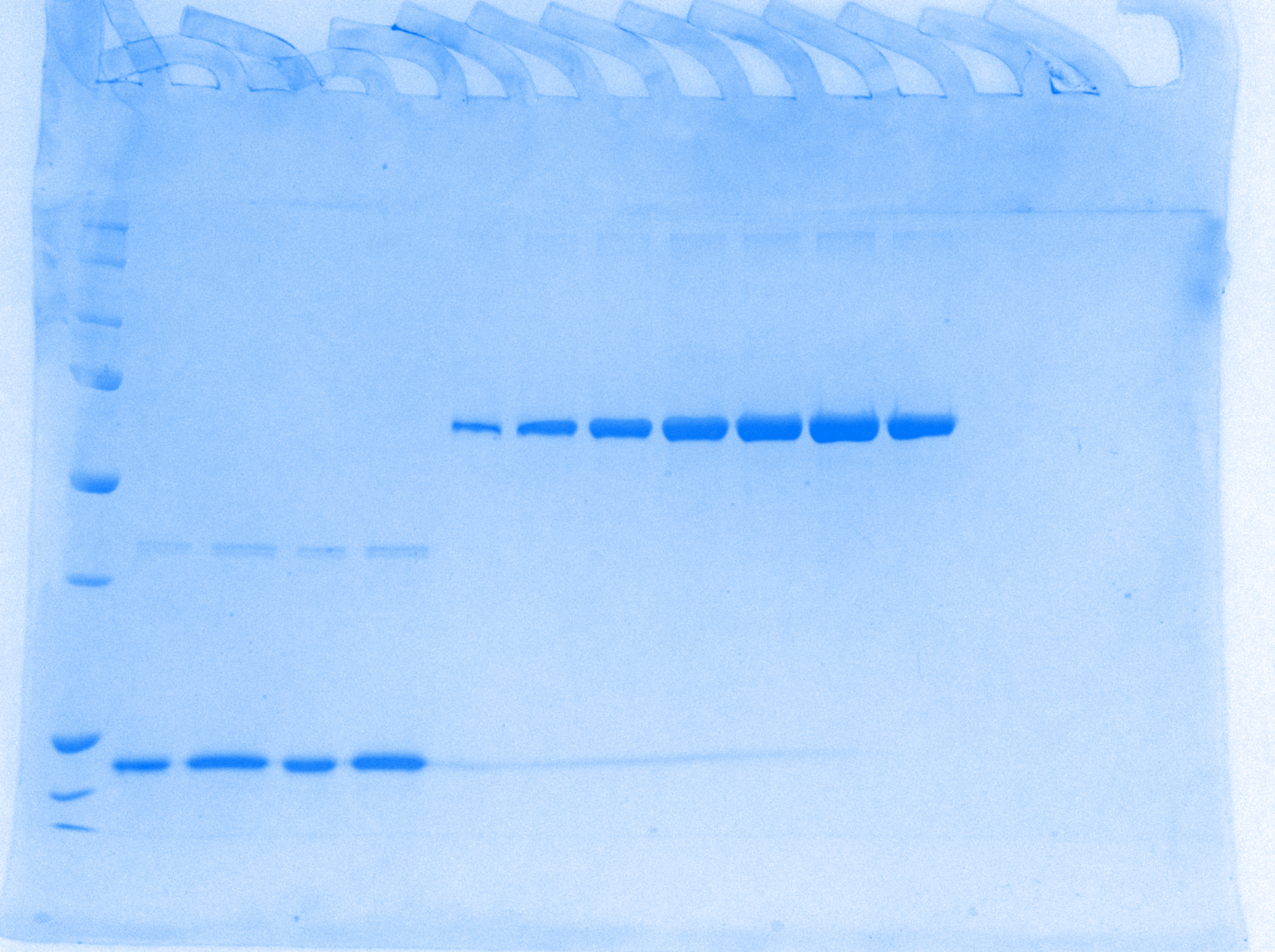

Supplement: Figure 4—figure supplement 3—source data 1. — KRAS probed with RAS (E8N8L) XP Rabbit mAb (Cell Signaling #67648); NTs probed with anti-His antibody. [file elife-88836-fig4-figsupp3-data1.zip › Figure 4- figure supplement 3- source data 1/KRAS quant.tif]

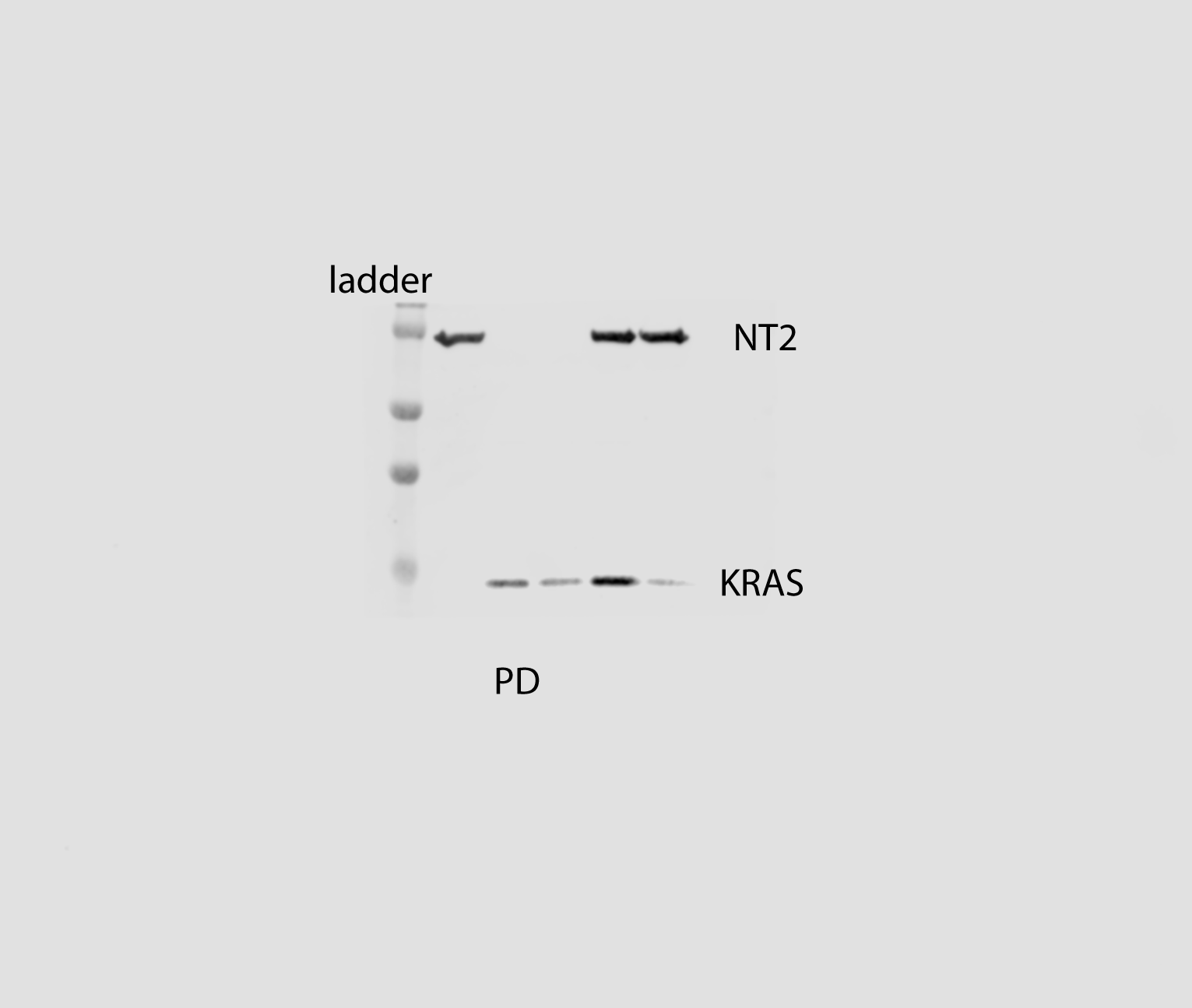

Supplement: Figure 4—figure supplement 3—source data 1. — KRAS probed with RAS (E8N8L) XP Rabbit mAb (Cell Signaling #67648); NTs probed with anti-His antibody. [file elife-88836-fig4-figsupp3-data1.zip › Figure 4- figure supplement 3- source data 1/NT2_K2_PD_1-24-23_low bckgrd-01.png]

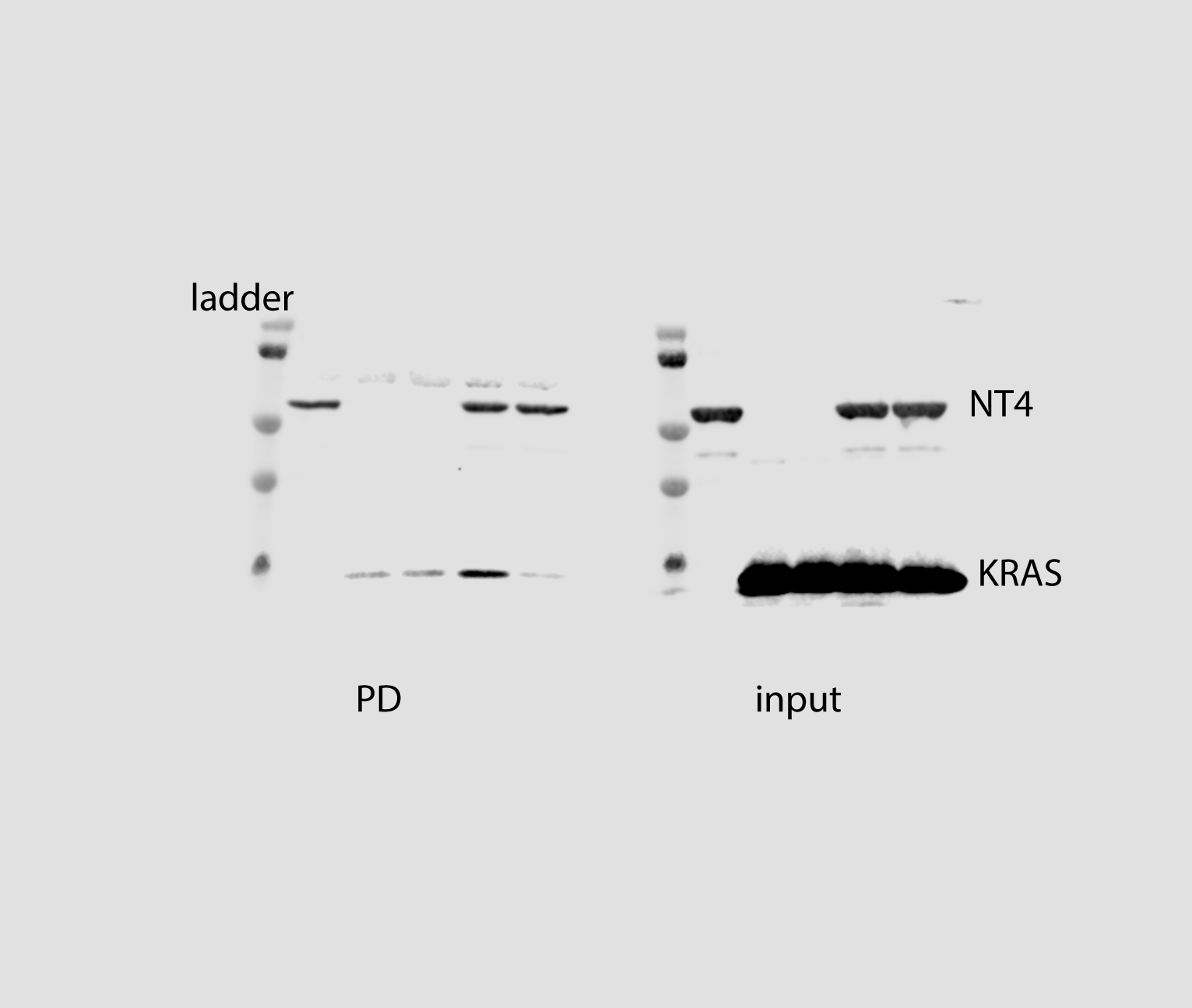

Supplement: Figure 4—figure supplement 3—source data 1. — KRAS probed with RAS (E8N8L) XP Rabbit mAb (Cell Signaling #67648); NTs probed with anti-His antibody. [file elife-88836-fig4-figsupp3-data1.zip › Figure 4- figure supplement 3- source data 1/NT4_K2_PD-left_inp-right-01.png]

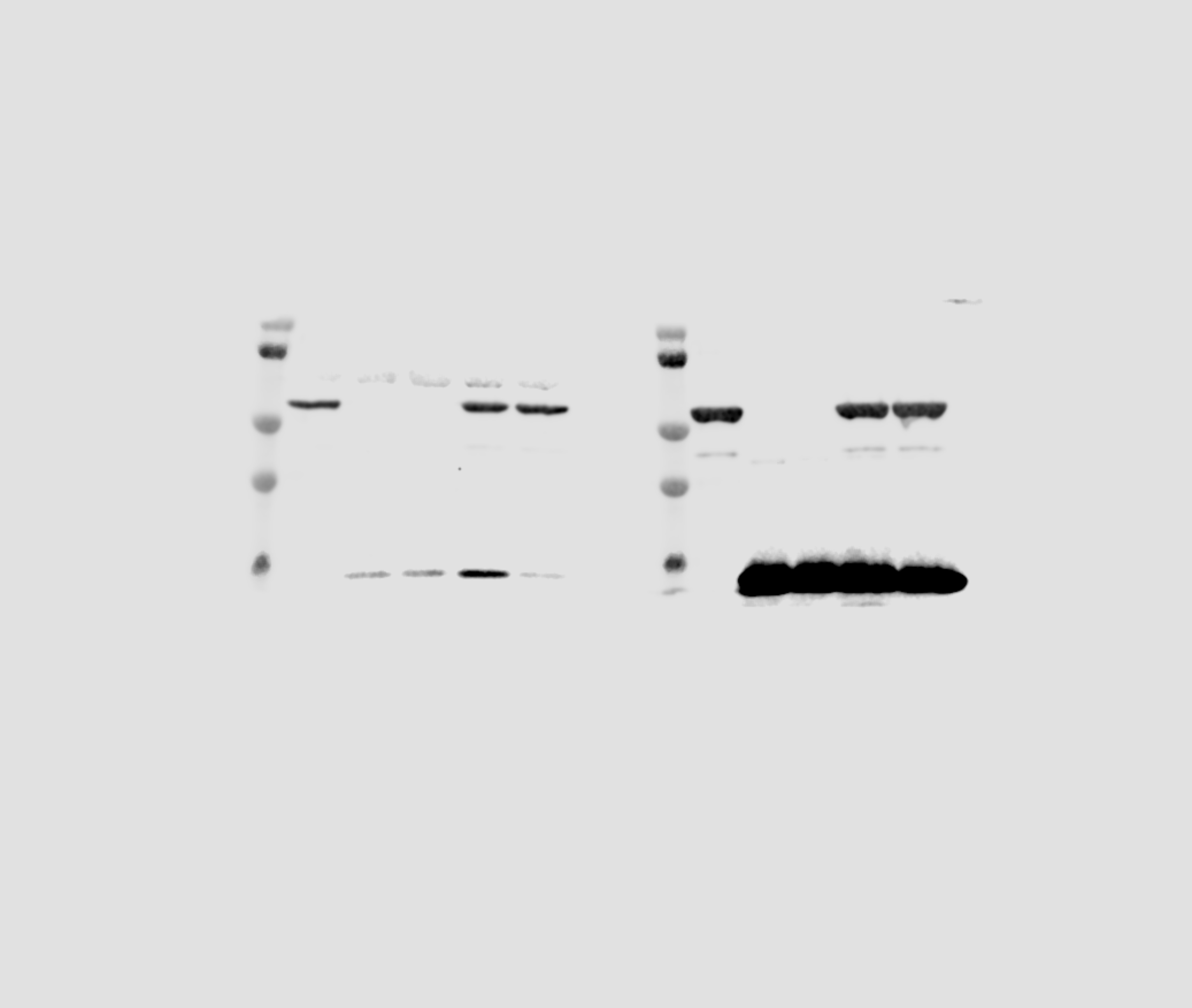

Supplement: Figure 4—figure supplement 3—source data 1. — KRAS probed with RAS (E8N8L) XP Rabbit mAb (Cell Signaling #67648); NTs probed with anti-His antibody. [file elife-88836-fig4-figsupp3-data1.zip › Figure 4- figure supplement 3- source data 1/NT4_K2_PD-left_inp-right.tif]

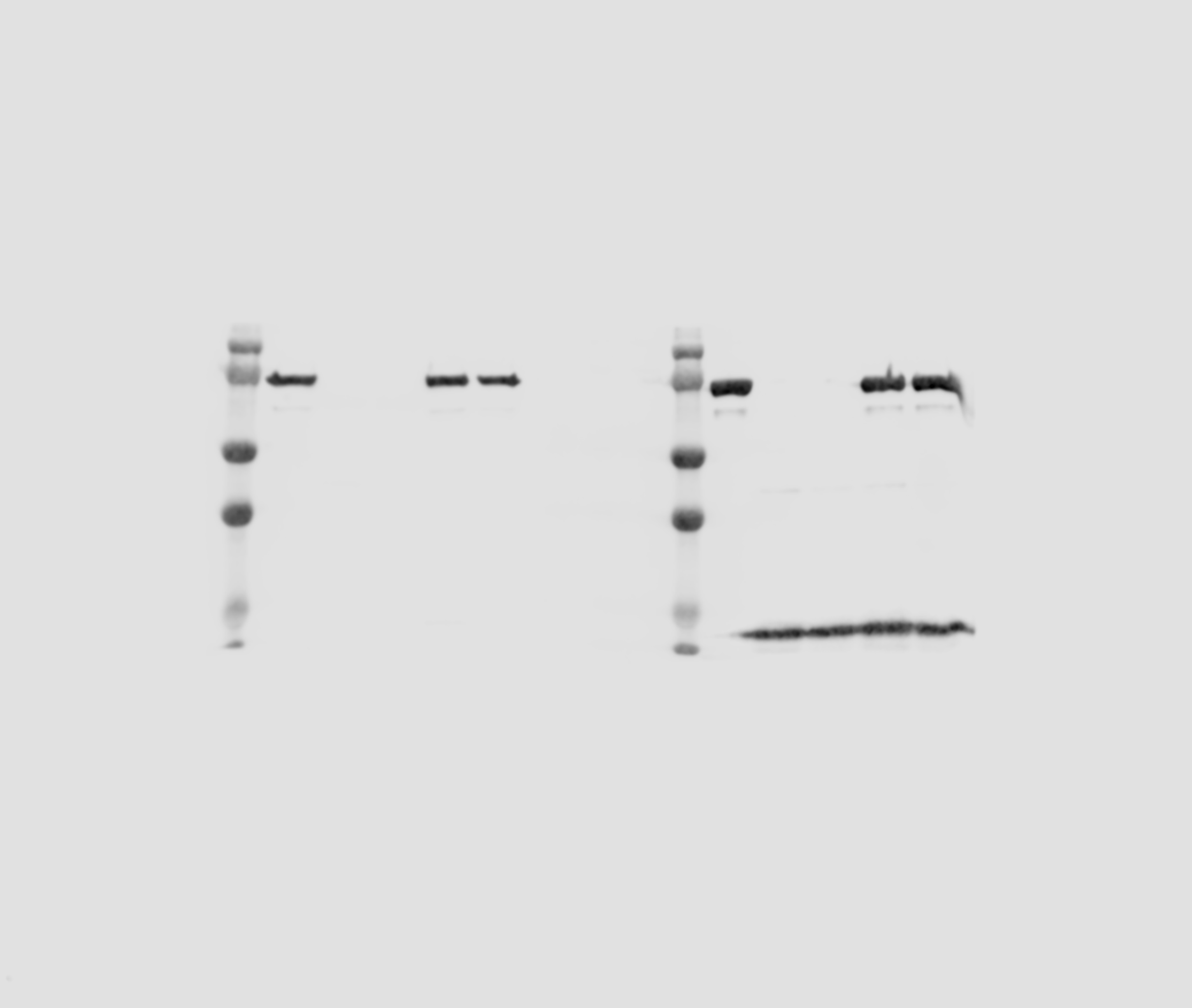

Supplement: Figure 4—figure supplement 3—source data 1. — KRAS probed with RAS (E8N8L) XP Rabbit mAb (Cell Signaling #67648); NTs probed with anti-His antibody. [file elife-88836-fig4-figsupp3-data1.zip › Figure 4- figure supplement 3- source data 1/NT2_K2_inp-right.tif]

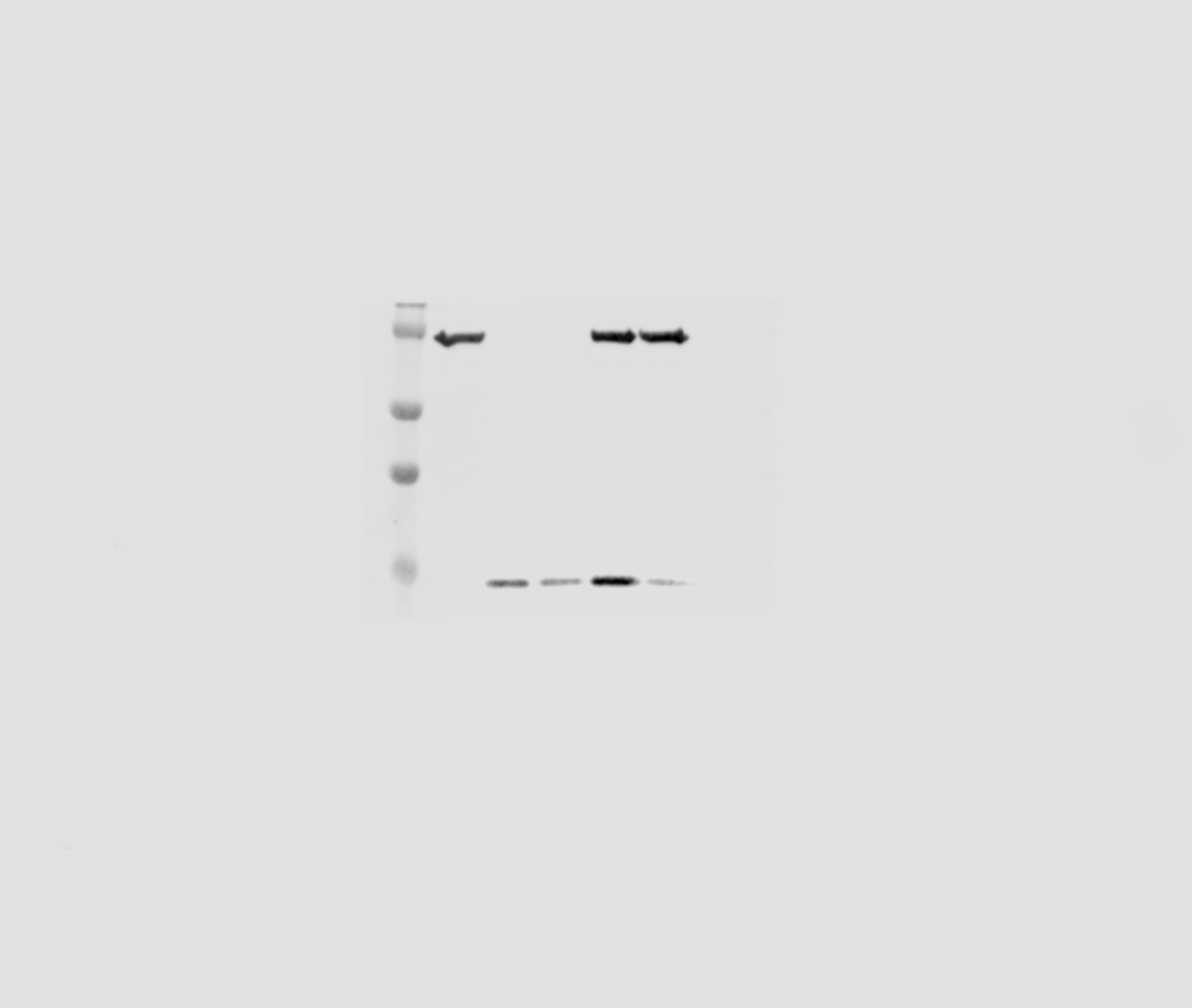

Supplement: Figure 4—figure supplement 3—source data 1. — KRAS probed with RAS (E8N8L) XP Rabbit mAb (Cell Signaling #67648); NTs probed with anti-His antibody. [file elife-88836-fig4-figsupp3-data1.zip › Figure 4- figure supplement 3- source data 1/NT2_K2_PD_1-24-23_low bckgrd.tif]

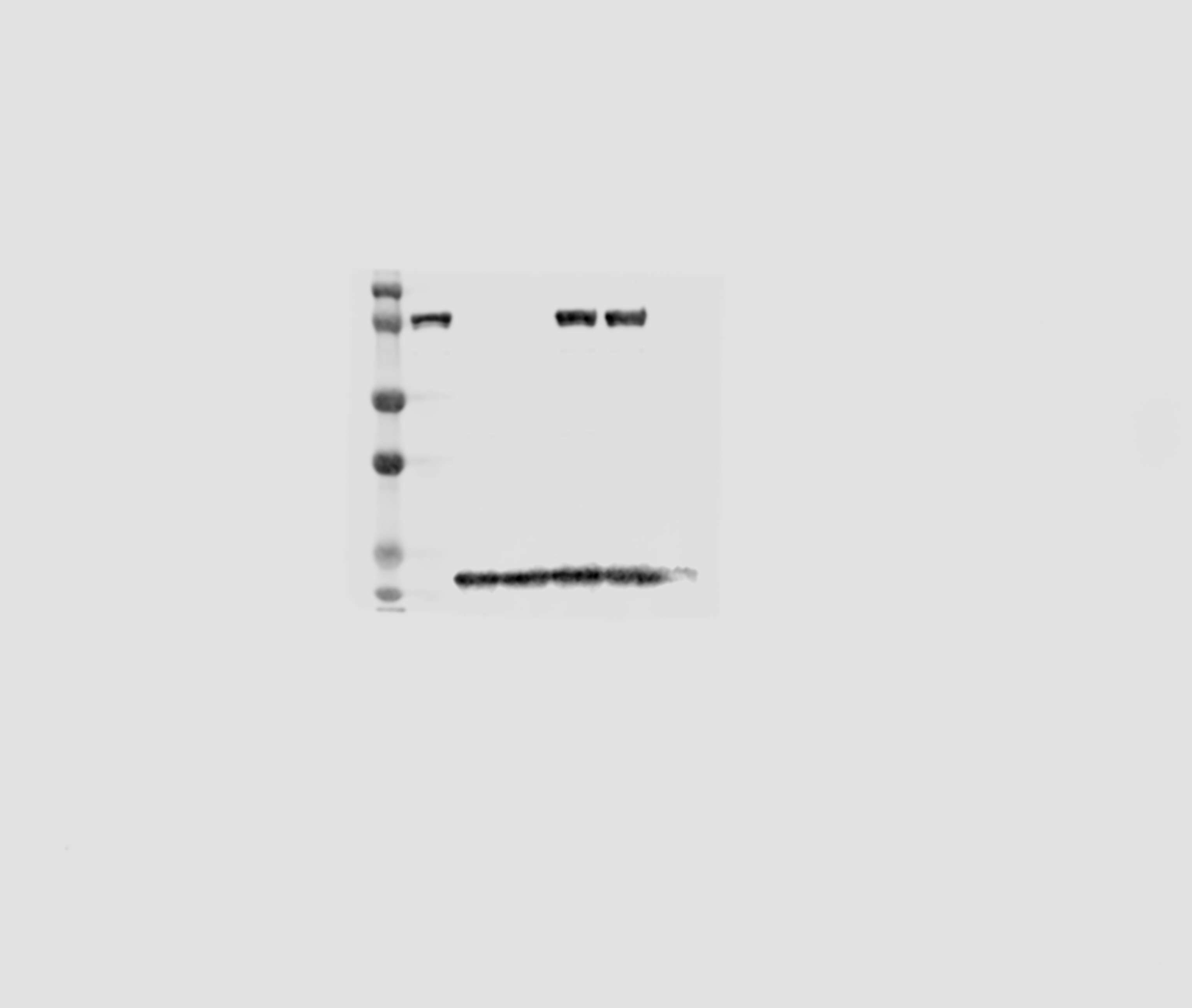

Supplement: Figure 4—figure supplement 3—source data 1. — KRAS probed with RAS (E8N8L) XP Rabbit mAb (Cell Signaling #67648); NTs probed with anti-His antibody. [file elife-88836-fig4-figsupp3-data1.zip › Figure 4- figure supplement 3- source data 1/NT1_K2_inp.tif]

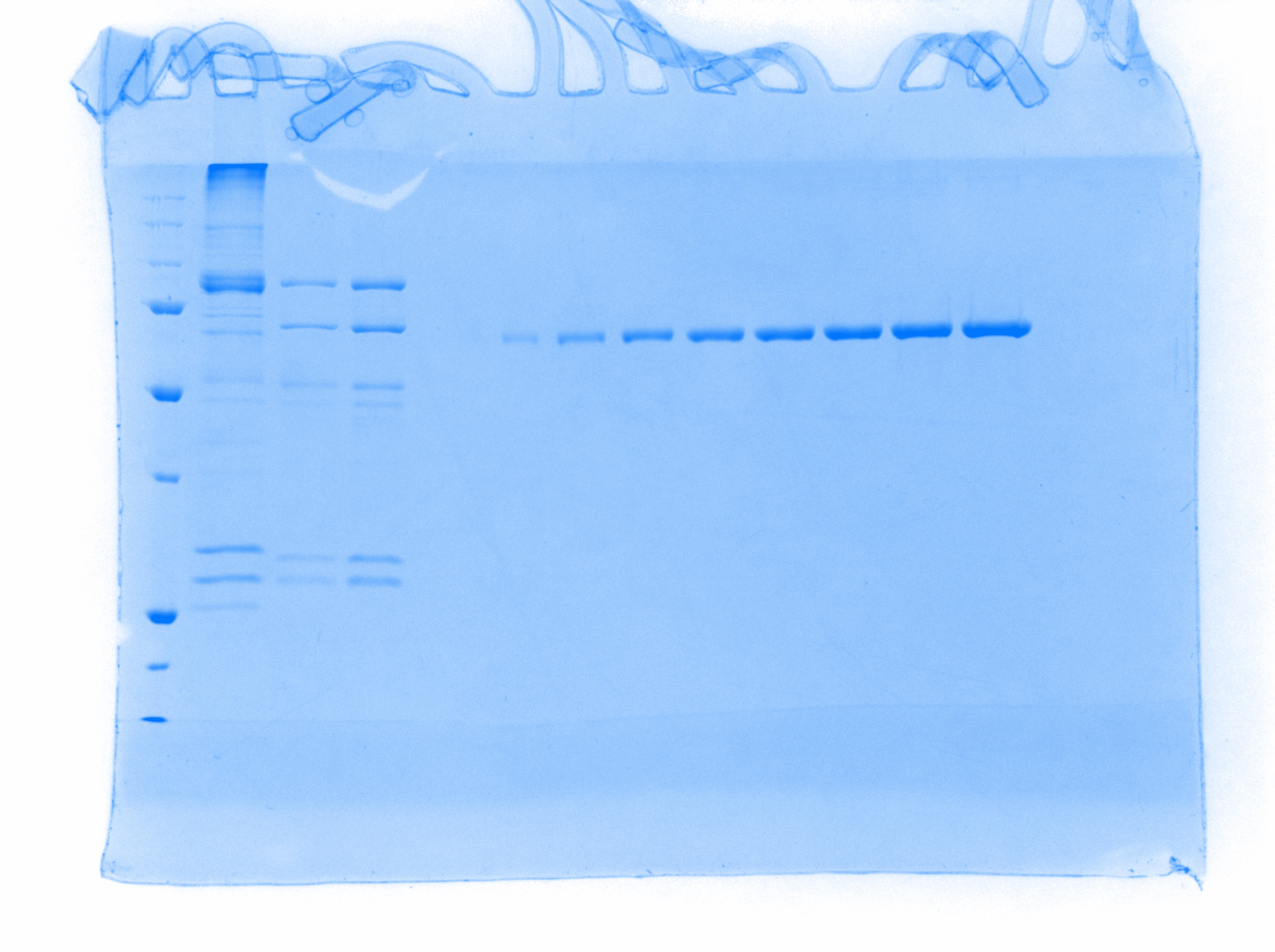

Supplement: Figure 4—figure supplement 4—source data 1. [file elife-88836-fig4-figsupp4-data1.zip › Figure 4- figure supplement 4- source data 1/FL-WT-BRAF quant 6-23-22.tif]

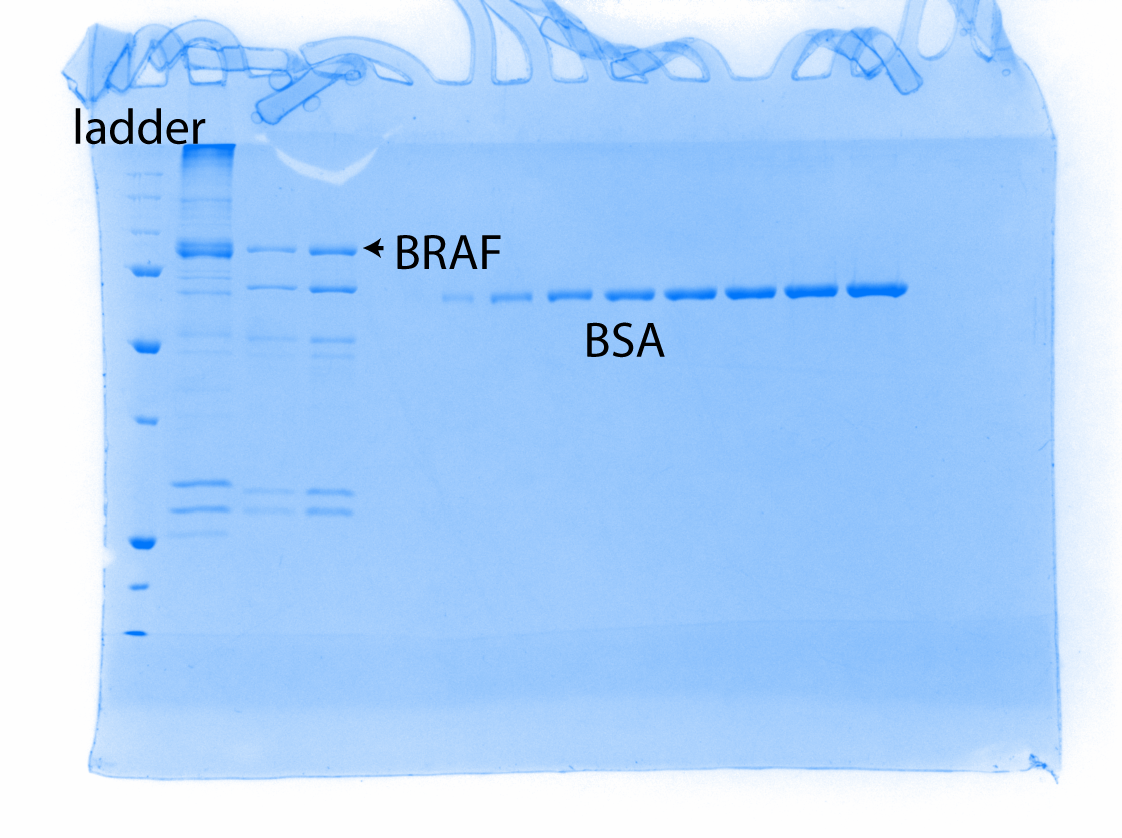

Supplement: Figure 4—figure supplement 4—source data 1. [file elife-88836-fig4-figsupp4-data1.zip › Figure 4- figure supplement 4- source data 1/FL-WT-BRAF quant 6-23-22-01.png]

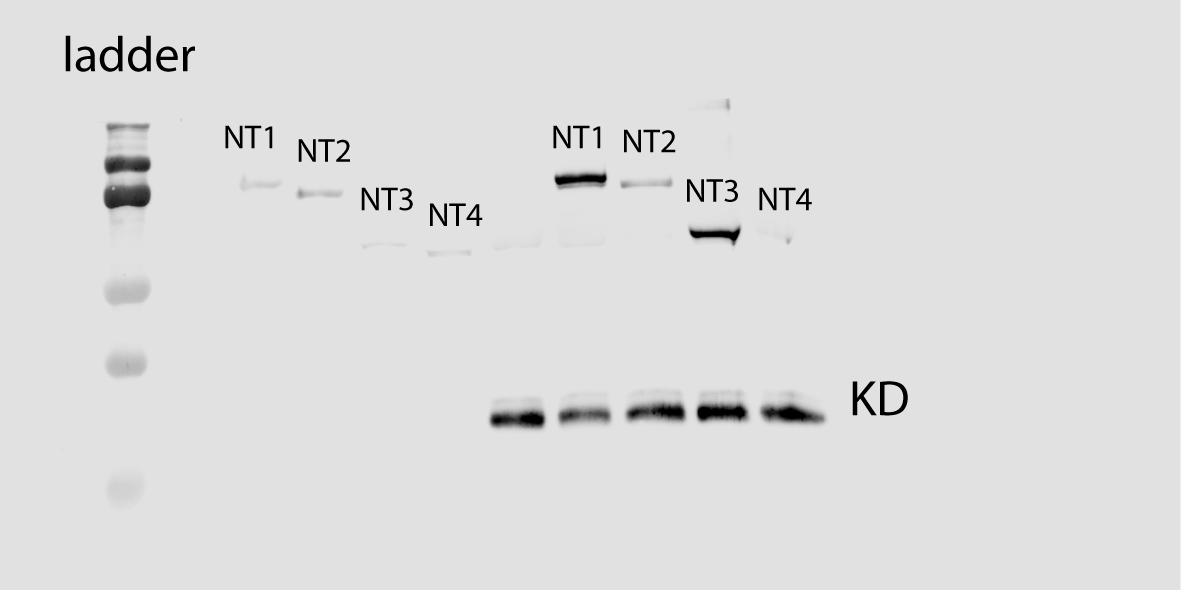

Supplement: Figure 5—source data 1. — KD and NTs probed with anti-His antibody. [file elife-88836-fig5-data1.zip › Figure 5- source data 1/btn-KD_NTs_PD-01.png]

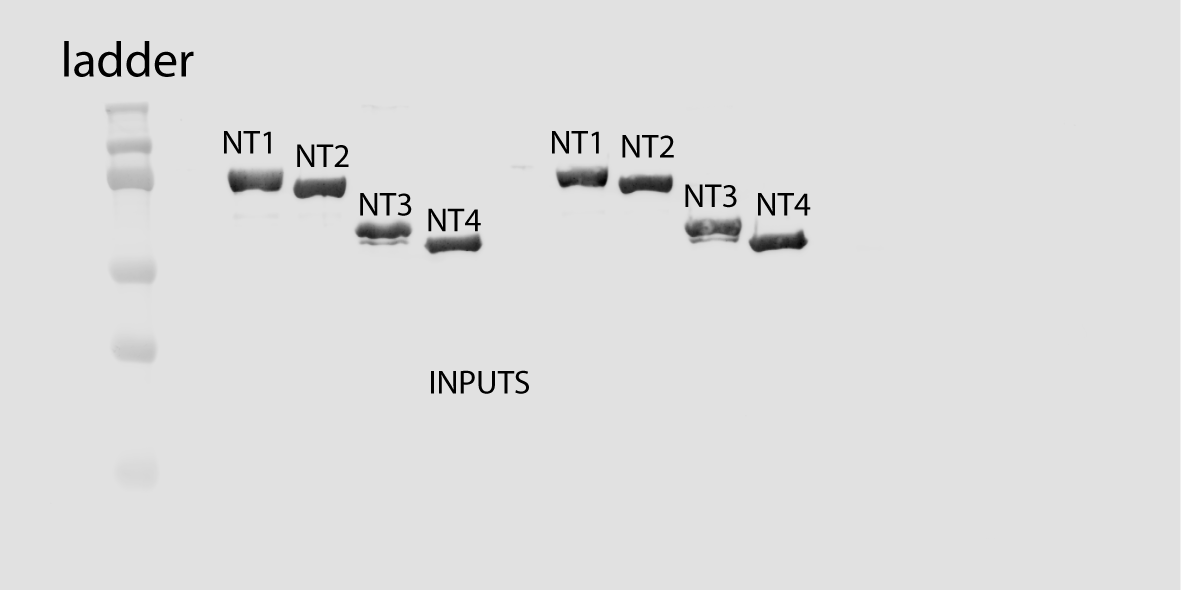

Supplement: Figure 5—source data 1. — KD and NTs probed with anti-His antibody. [file elife-88836-fig5-data1.zip › Figure 5- source data 1/btn-KD_NTs_INPUT-01.png]

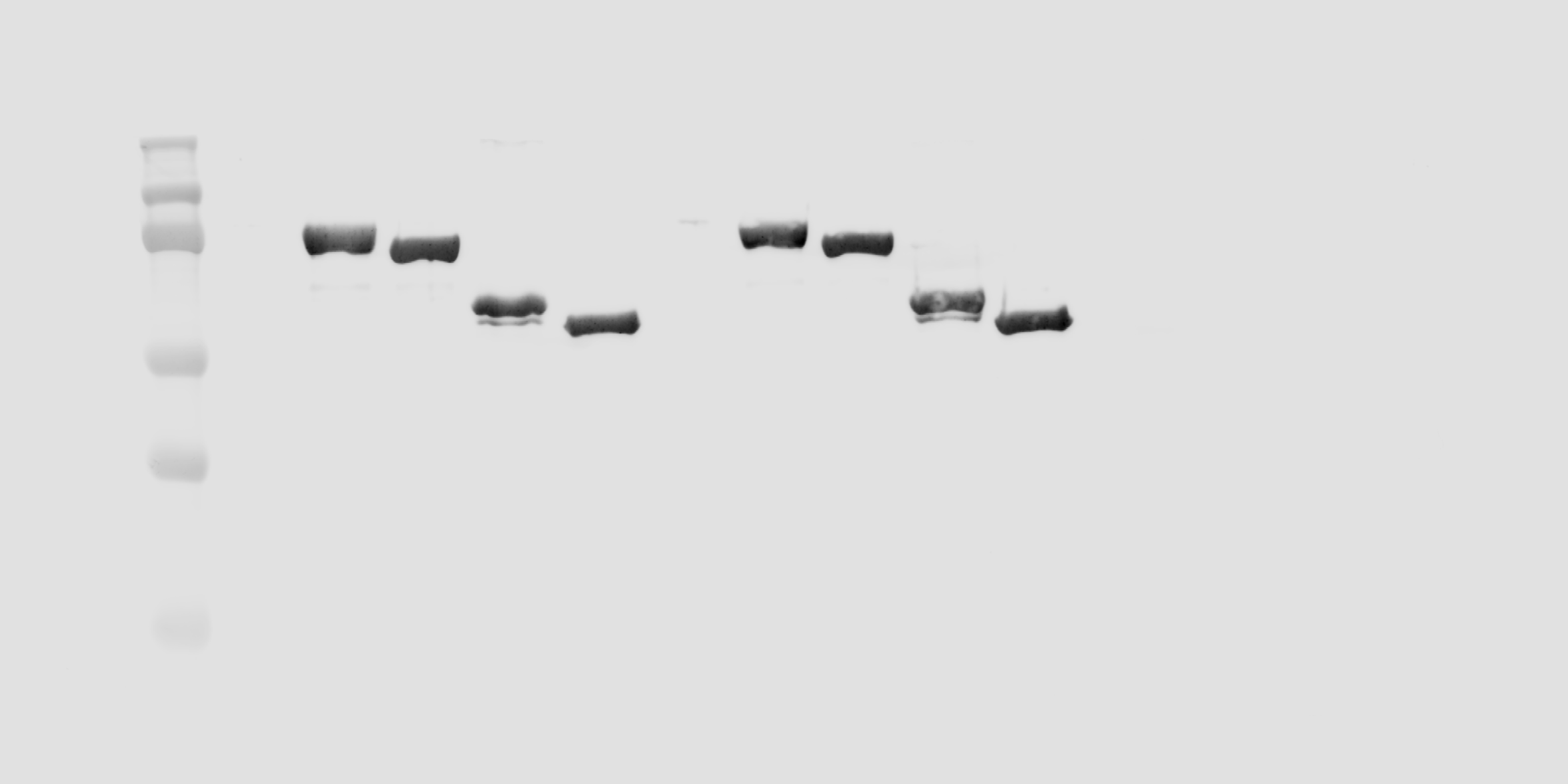

Supplement: Figure 5—source data 1. — KD and NTs probed with anti-His antibody. [file elife-88836-fig5-data1.zip › Figure 5- source data 1/btn-KD_NTs_INPUT.tif]

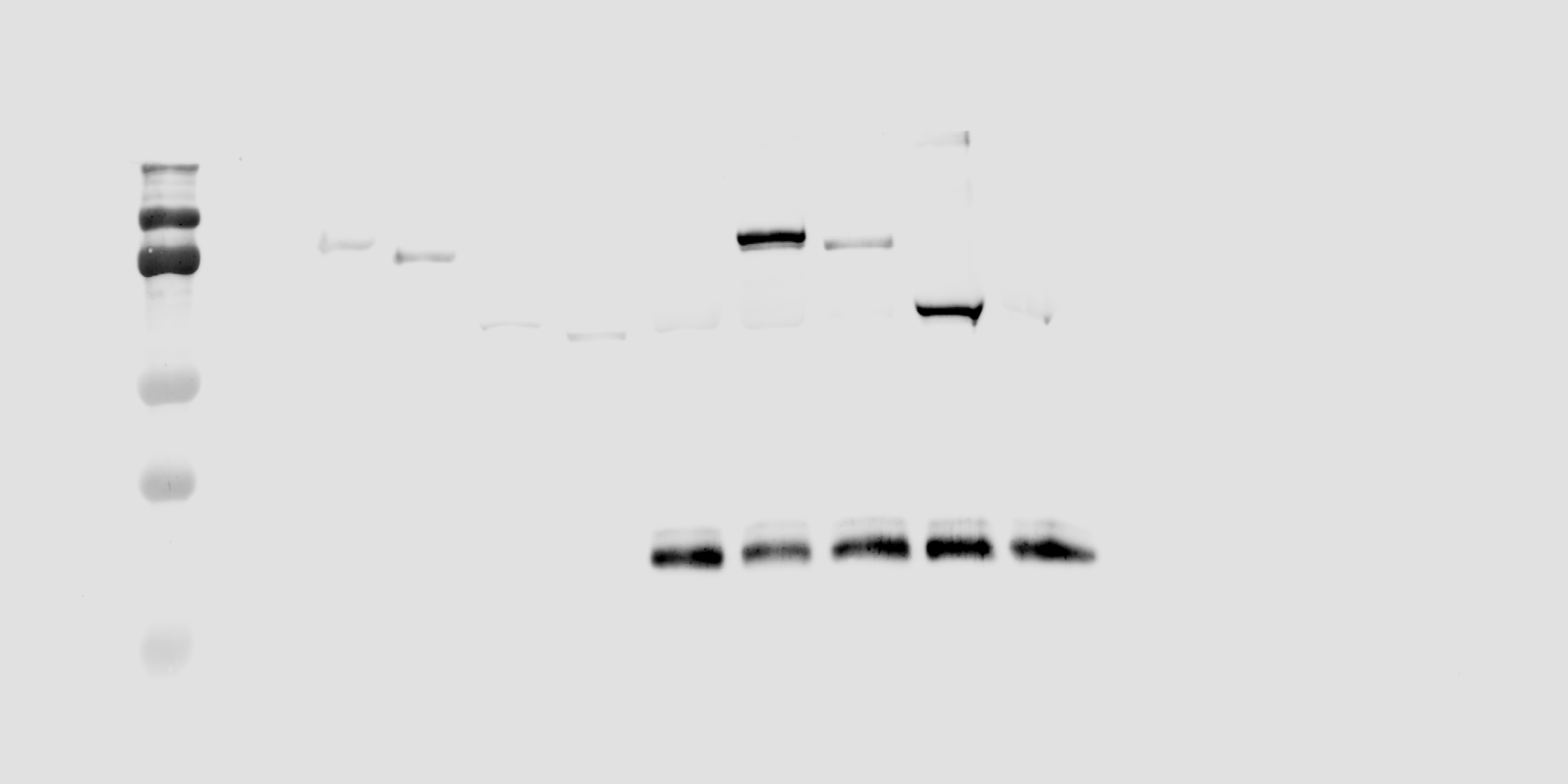

Supplement: Figure 5—source data 1. — KD and NTs probed with anti-His antibody. [file elife-88836-fig5-data1.zip › Figure 5- source data 1/btn-KD_NTs_PD.tif]

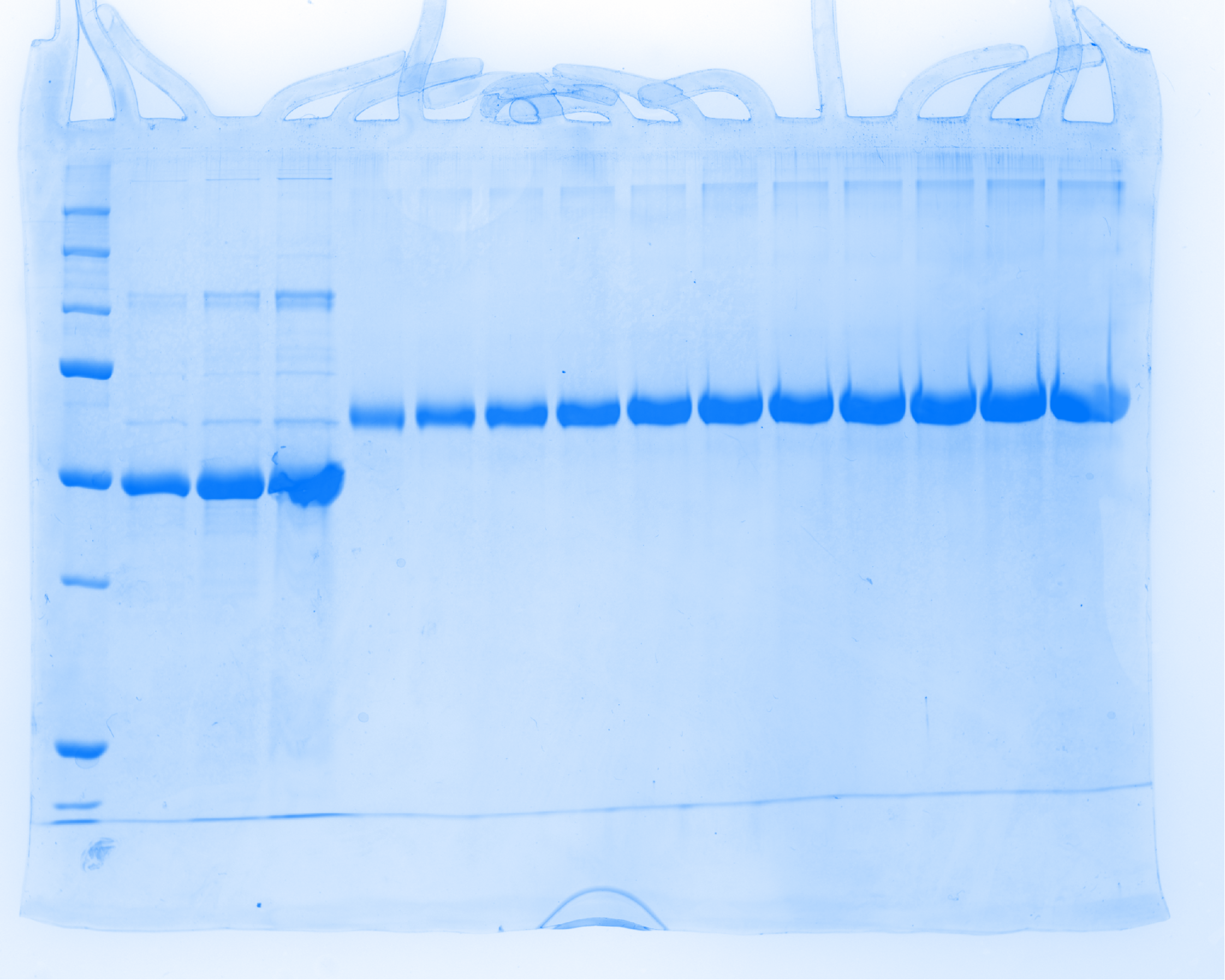

Supplement: Figure 5—source data 3. [file elife-88836-fig5-data3.zip › Figure 5- source data 3/NT5 final quant.tif]

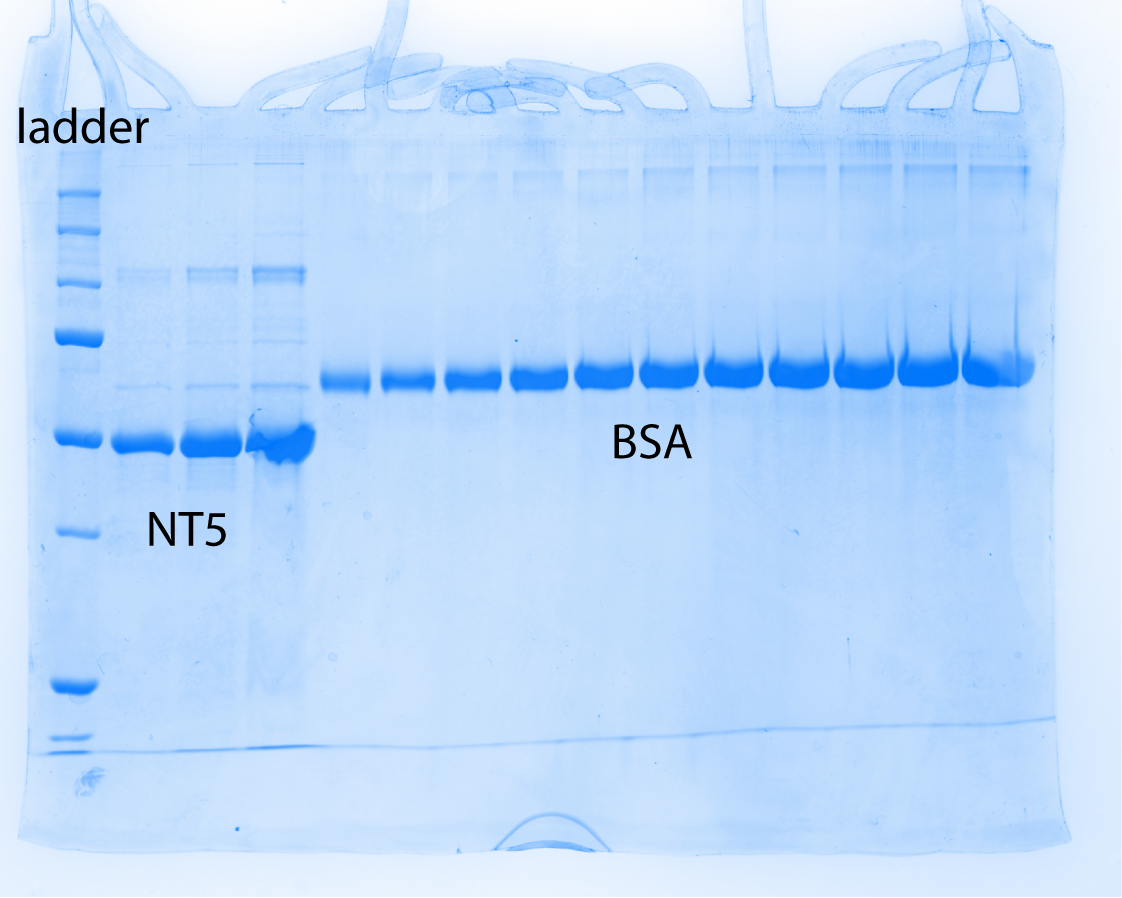

Supplement: Figure 5—source data 3. [file elife-88836-fig5-data3.zip › Figure 5- source data 3/NT5 final quant-01.png]

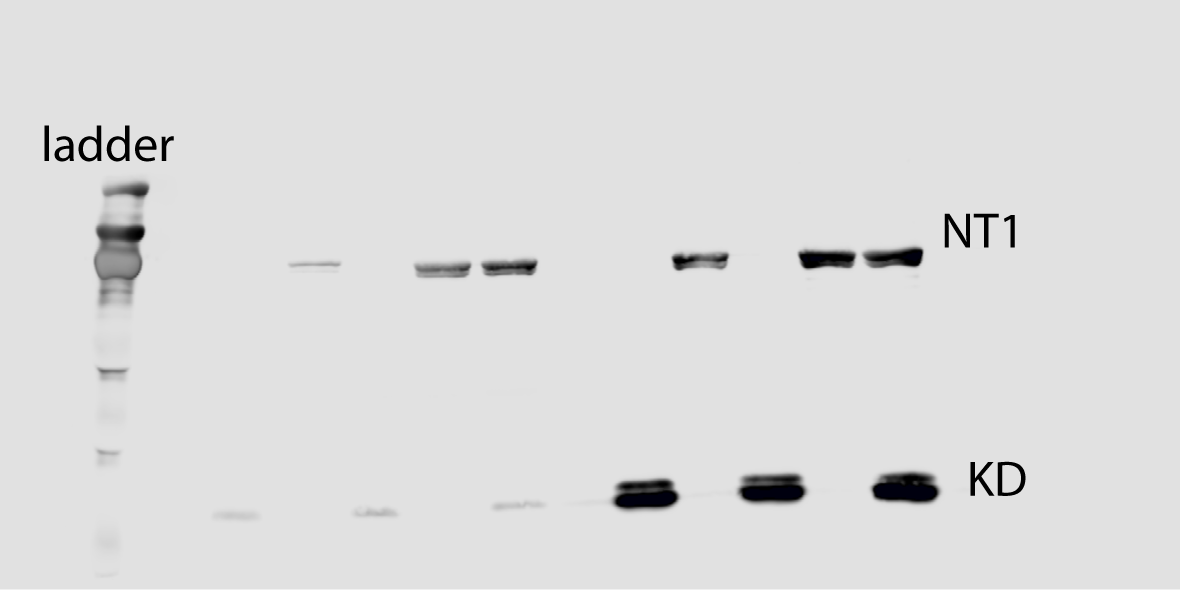

Supplement: Figure 6—source data 1. — HRAS probed with anti-GST antibody; KD and NT1 probed with anti-His antibody. [file elife-88836-fig6-data1.zip › Figure 6- source data 1/HRAS_1-288_KD PD-01.png]

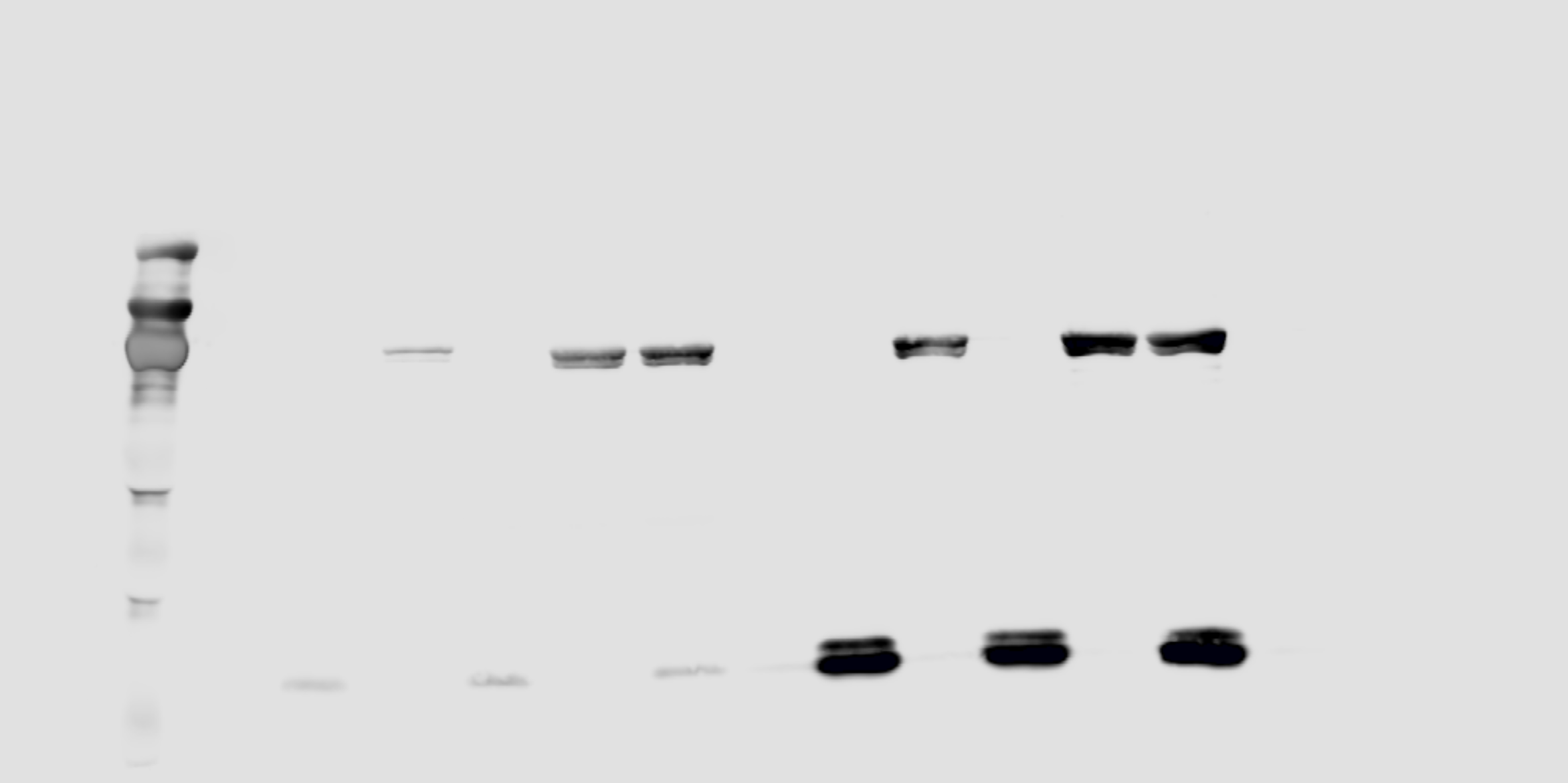

Supplement: Figure 6—source data 1. — HRAS probed with anti-GST antibody; KD and NT1 probed with anti-His antibody. [file elife-88836-fig6-data1.zip › Figure 6- source data 1/HRAS_1-288_KD PD.tif]

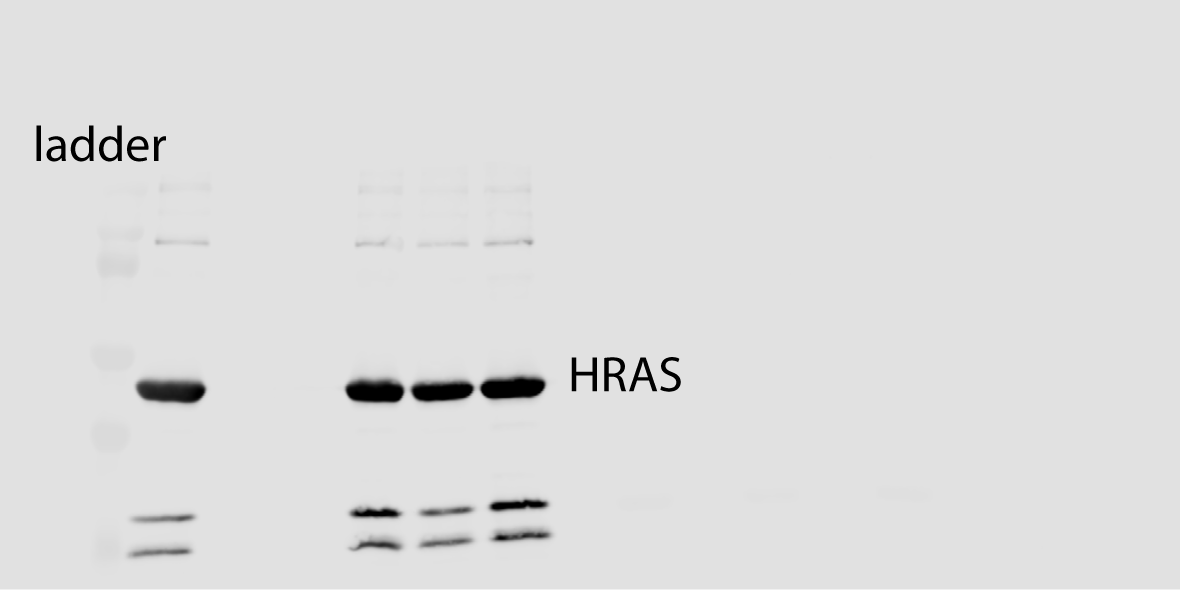

Supplement: Figure 6—source data 1. — HRAS probed with anti-GST antibody; KD and NT1 probed with anti-His antibody. [file elife-88836-fig6-data1.zip › Figure 6- source data 1/HRAS_1-288_KD GST PD-01.png]

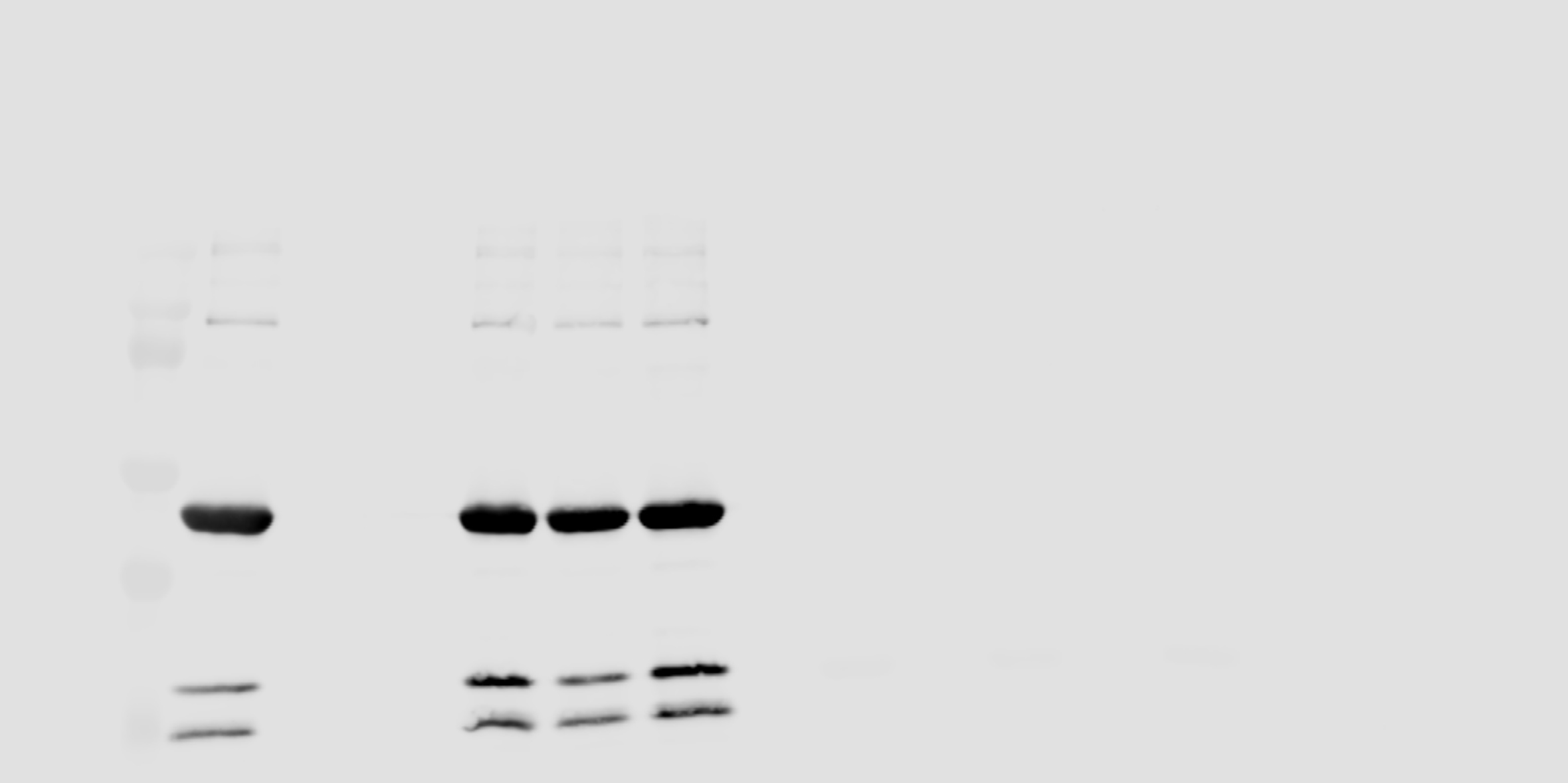

Supplement: Figure 6—source data 1. — HRAS probed with anti-GST antibody; KD and NT1 probed with anti-His antibody. [file elife-88836-fig6-data1.zip › Figure 6- source data 1/HRAS_1-288_KD GST PD.tif]

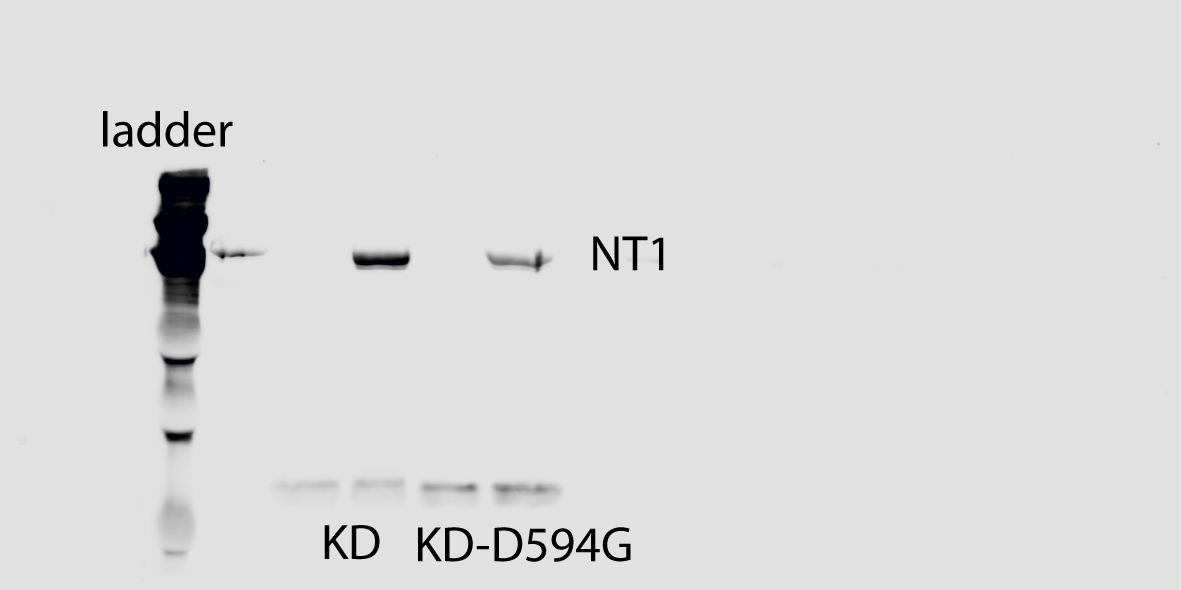

Supplement: Figure 6—source data 2. — KD and NTs probed with anti-His antibody. [file elife-88836-fig6-data2.zip › Figure 6- source data 2/btn-KD-WTvDG PD- 1-288-01.png]

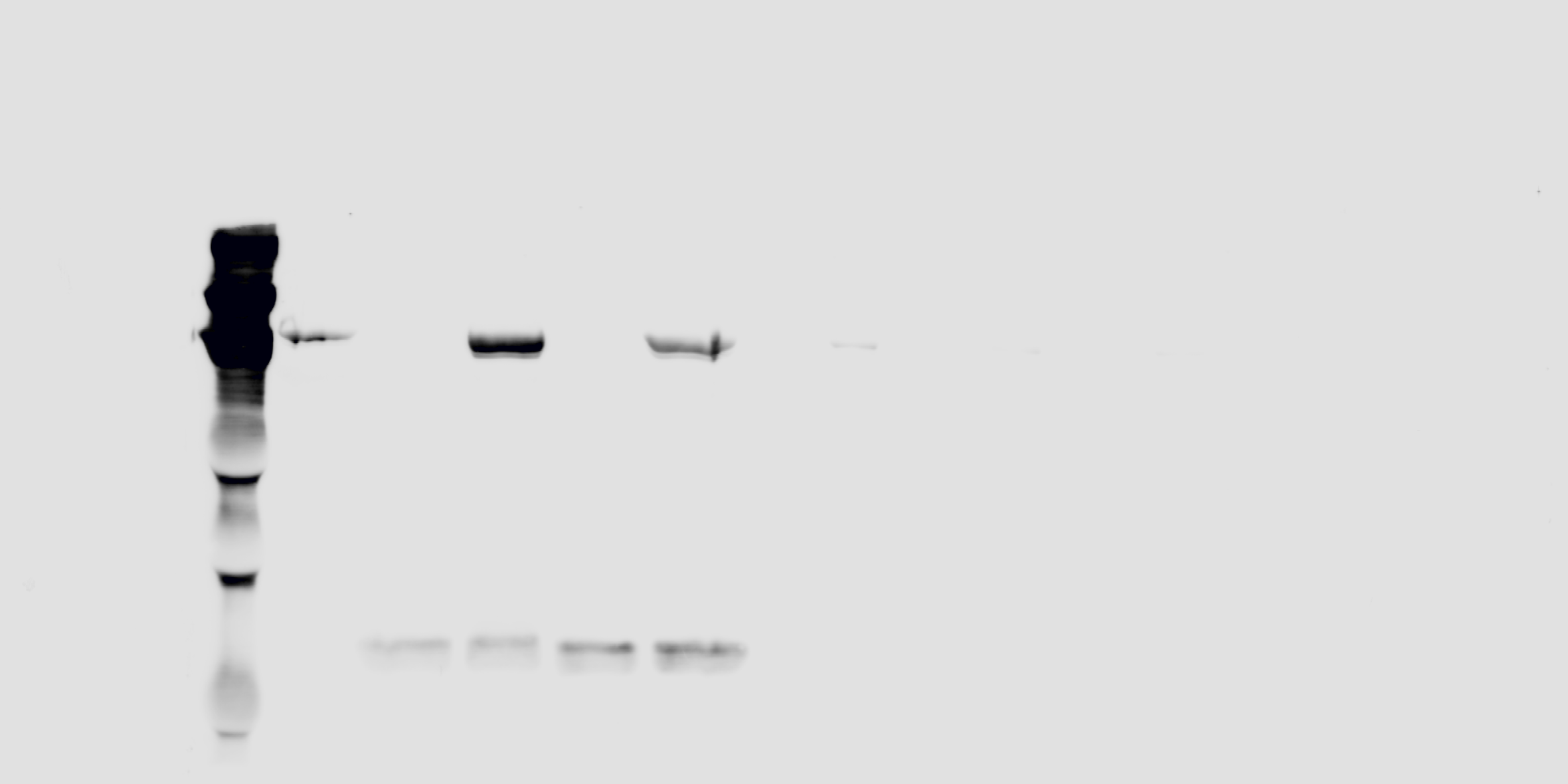

Supplement: Figure 6—source data 2. — KD and NTs probed with anti-His antibody. [file elife-88836-fig6-data2.zip › Figure 6- source data 2/btn-KD-WTvDG PD- 1-288.tif]
