# Supplementary material for: Unveiling the domain-specific and RAS isoform-specific details of BRAF kinase regulation
Source: eLife. 2023 Dec 27;12:RP88836. doi: 10.7554/eLife.88836 (PMC10752582; doi:10.7554/eLife.88836)
Supplement: Figure 3—source data 1. — Full test preview provided in .txt format for NT2, NT3, and NT4. Excel file of all replicate SPR runs included in this article. Refer to this document for Figures 3—6. [file elife-88836-fig3-data1.zip › Figure 3- source data 1/NT3_HRAS_12-2-21 fit.pdf]

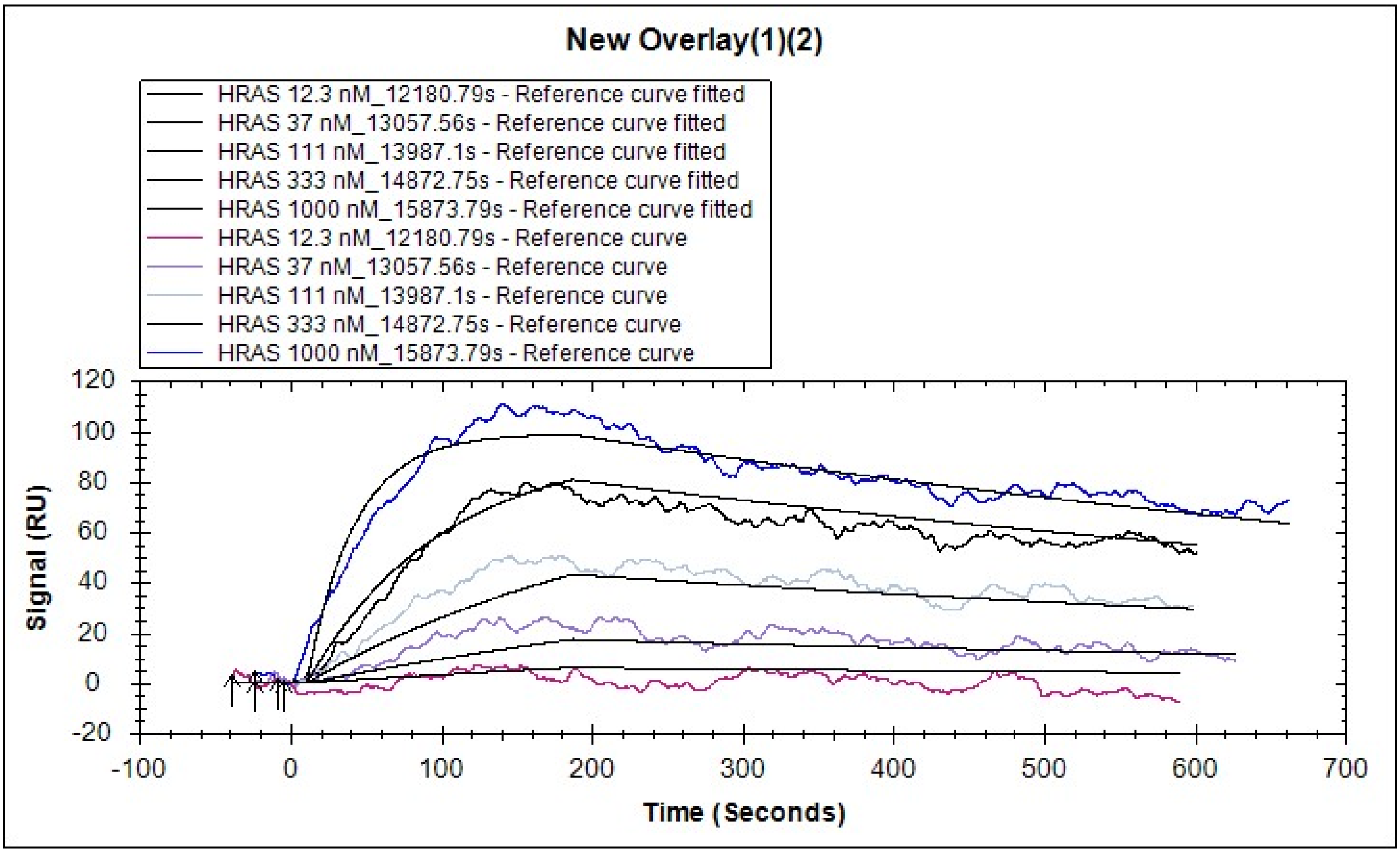

Evaluation type: OneToOne

Curve name	Bmax ([Signal (RU)])	ka (1/(M*s))	kd (1/s)	KD (M)
HRAS 12.3 nM_12180.79s - Reference curve fitted	101.98 (±2.33e-2)	3.09e4 (±3.60e2)	9.29e-4 (±1.11e-6)	3.01e-8 (±3.86e-10)
HRAS 37 nM_13057.56s - Reference curve fitted	101.98 (±2.33e-2)	3.09e4 (±3.60e2)	9.29e-4 (±1.11e-6)	3.01e-8 (±3.86e-10)
HRAS 111 nM_13987.1s - Reference curve fitted	101.98 (±2.33e-2)	3.09e4 (±3.60e2)	9.29e-4 (±1.11e-6)	3.01e-8 (±3.86e-10)
HRAS 333 nM_14872.75s - Reference curve fitted	101.98 (±2.33e-2)	3.09e4 (±3.60e2)	9.29e-4 (±1.11e-6)	3.01e-8 (±3.86e-10)
HRAS 1000 nM_15873.79s - Reference curve fitted	101.98 (±2.33e-2)	3.09e4 (±3.60e2)	9.29e-4 (±1.11e-6)	3.01e-8 (±3.86e-10)

Curve name	BI ([Signal (RU)])	Chi2 ([Signal (RU)]^2)	U-value: kd (%)
HRAS 12.3 nM_12180.79s - Reference curve fitted	0.10	29.01	5.00
HRAS 37 nM_13057.56s - Reference curve fitted	0.10	29.01	5.00
HRAS 111 nM_13987.1s - Reference curve fitted	0.10	29.01	5.00
HRAS 333 nM_14872.75s - Reference curve fitted	0.10	29.01	5.00
HRAS 1000 nM_15873.79s - Reference curve fitted	0.10	29.01	5.00

Run	Date	Source
New Overlay(1)	-	New Overlay(1)
