# Supplementary material for: Unveiling the domain-specific and RAS isoform-specific details of BRAF kinase regulation
Source: eLife. 2023 Dec 27;12:RP88836. doi: 10.7554/eLife.88836 (PMC10752582; doi:10.7554/eLife.88836)
Supplement: Figure 3—source data 1. — Full test preview provided in .txt format for NT2, NT3, and NT4. Excel file of all replicate SPR runs included in this article. Refer to this document for Figures 3—6. [file elife-88836-fig3-data1.zip › Figure 3- source data 1/NT4_HRAS_ 4-11-22 fit.pdf]

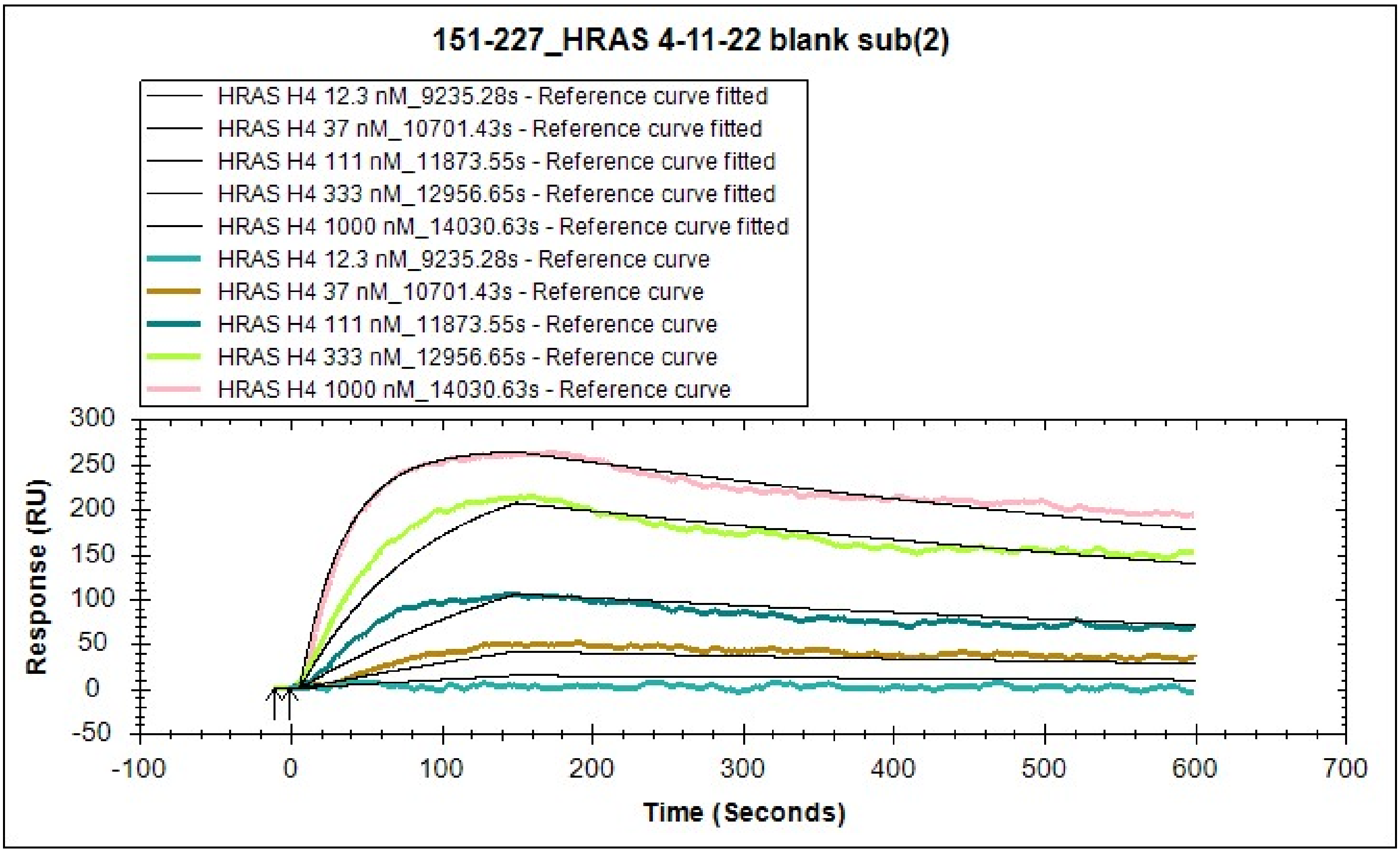

Evaluation type: OneToOne

Curve name	Bmax ([Signal (RU)])	ka (1/(M*s))	kd (1/s)
HRAS H4 12.3 nM_9235.28s - Reference curve fitted	272.84 (±2.81e-2)	3.30e4 (±7.10e1)	8.80e-4 (±1.99e-6)
HRAS H4 37 nM_10701.43s - Reference curve fitted	272.84 (±2.81e-2)	3.30e4 (±7.10e1)	8.80e-4 (±1.99e-6)
HRAS H4 111 nM_11873.55s - Reference curve fitted	272.84 (±2.81e-2)	3.30e4 (±7.10e1)	8.80e-4 (±1.99e-6)
HRAS H4 333 nM_12956.65s - Reference curve fitted	272.84 (±2.81e-2)	3.30e4 (±7.10e1)	8.80e-4 (±1.99e-6)
HRAS H4 1000 nM_14030.63s - Reference curve fitted	272.84 (±2.81e-2)	3.30e4 (±7.10e1)	8.80e-4 (±1.99e-6)

Curve name	KD (M)	BI ([Signal (RU)])	Chi2 ([Signal (RU)]^2)
HRAS H4 12.3 nM_9235.28s - Reference curve fitted	2.66e-8 (±1.18e-10)	0.10	97.70
HRAS H4 37 nM_10701.43s - Reference curve fitted	2.66e-8 (±1.18e-10)	0.10	97.70
HRAS H4 111 nM_11873.55s - Reference curve fitted	2.66e-8 (±1.18e-10)	0.10	97.70
HRAS H4 333 nM_12956.65s - Reference curve fitted	2.66e-8 (±1.18e-10)	0.10	97.70
HRAS H4 1000 nM_14030.63s - Reference curve fitted	2.66e-8 (±1.18e-10)	0.10	97.70

Curve name	U-value: kd (%)
HRAS H4 12.3 nM_9235.28s - Reference curve fitted	2.70
HRAS H4 37 nM_10701.43s - Reference curve fitted	2.70
HRAS H4 111 nM_11873.55s - Reference curve fitted	2.70
HRAS H4 333 nM_12956.65s - Reference curve fitted	2.70
HRAS H4 1000 nM_14030.63s - Reference curve fitted	2.70

Run	Date	Source
New Overlay(2)	-	New Overlay(2)
