# Supplementary material for: Unveiling the domain-specific and RAS isoform-specific details of BRAF kinase regulation
Source: eLife. 2023 Dec 27;12:RP88836. doi: 10.7554/eLife.88836 (PMC10752582; doi:10.7554/eLife.88836)
Supplement: Figure 3—source data 1. — Full test preview provided in .txt format for NT2, NT3, and NT4. Excel file of all replicate SPR runs included in this article. Refer to this document for Figures 3—6. [file elife-88836-fig3-data1.zip › Figure 3- source data 1/NT2_HRAS_3-15-22 fit.pdf]

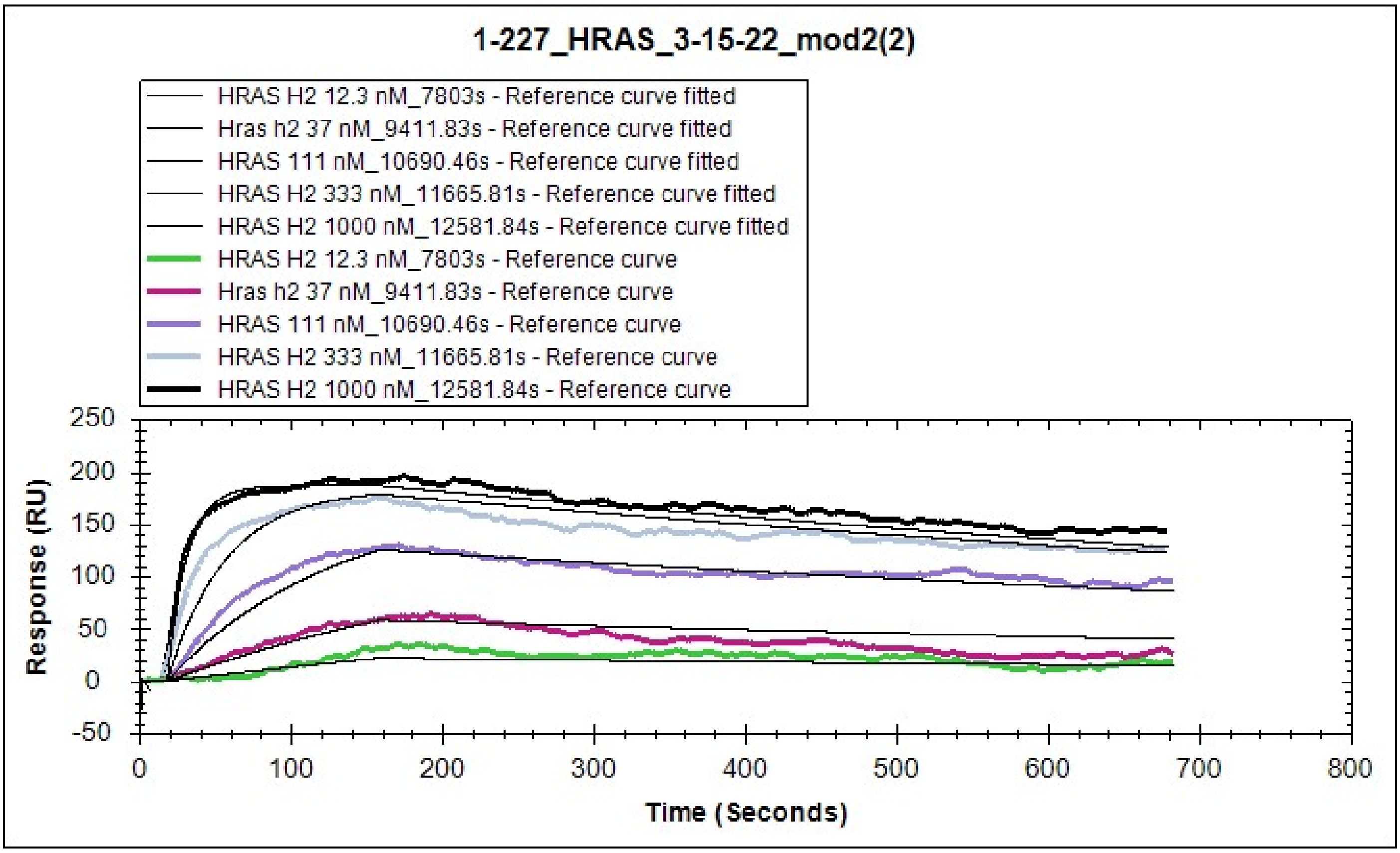

Evaluation type: OneToOne

Curve name	Bmax ([Response (RU)])	ka (1/(M*s))	kd (1/s)
HRAS H2 12.3 nM_7803s - Reference curve fitted	188.57 (±1.99e-2)	7.56e4 (±1.26e2)	7.24e-4 (±1.88e-6)
Hras h2 37 nM_9411.83s - Reference curve fitted	188.57 (±1.99e-2)	7.56e4 (±1.26e2)	7.24e-4 (±1.88e-6)
HRAS 111 nM_10690.46s - Reference curve fitted	188.57 (±1.99e-2)	7.56e4 (±1.26e2)	7.24e-4 (±1.88e-6)
HRAS H2 333 nM_11665.81s - Reference curve fitted	188.57 (±1.99e-2)	7.56e4 (±1.26e2)	7.24e-4 (±1.88e-6)
HRAS H2 1000 nM_12581.84s - Reference curve fitted	188.57 (±1.99e-2)	7.56e4 (±1.26e2)	7.24e-4 (±1.88e-6)

Curve name	KD (M)	BI ([Response (RU)])	Chi2 ([Response (RU)]^2)
HRAS H2 12.3 nM_7803s - Reference curve fitted	9.58e-9 (±4.08e-11)	0.10	81.28
Hras h2 37 nM_9411.83s - Reference curve fitted	9.58e-9 (±4.08e-11)	0.10	81.28
HRAS 111 nM_10690.46s - Reference curve fitted	9.58e-9 (±4.08e-11)	0.10	81.28
HRAS H2 333 nM_11665.81s - Reference curve fitted	9.58e-9 (±4.08e-11)	0.10	81.28
HRAS H2 1000 nM_12581.84s - Reference curve fitted	9.58e-9 (±4.08e-11)	0.10	81.28

Curve name	U-value: kd (%)
HRAS H2 12.3 nM_7803s - Reference curve fitted	3.00
Hras h2 37 nM_9411.83s - Reference curve fitted	3.00
HRAS 111 nM_10690.46s - Reference curve fitted	3.00
HRAS H2 333 nM_11665.81s - Reference curve fitted	3.00
HRAS H2 1000 nM_12581.84s - Reference curve fitted	3.00

Run	Date	Source
1-227_HRAS_3-15-22_mod2	-	New Overlay(1)
