# Supplementary material for: Unveiling the domain-specific and RAS isoform-specific details of BRAF kinase regulation
Source: eLife. 2023 Dec 27;12:RP88836. doi: 10.7554/eLife.88836 (PMC10752582; doi:10.7554/eLife.88836)
Supplement: Figure 4—source data 2. — Full test preview provided in .txt format for NT1, NT2, NT3, and NT4. NT2 data also applies for Figure 4—figure supplement 2. [file elife-88836-fig4-data2.zip › Figure 4- source data 2/NT1_GST-KRAS 3-21-23_TT_fit.pdf]

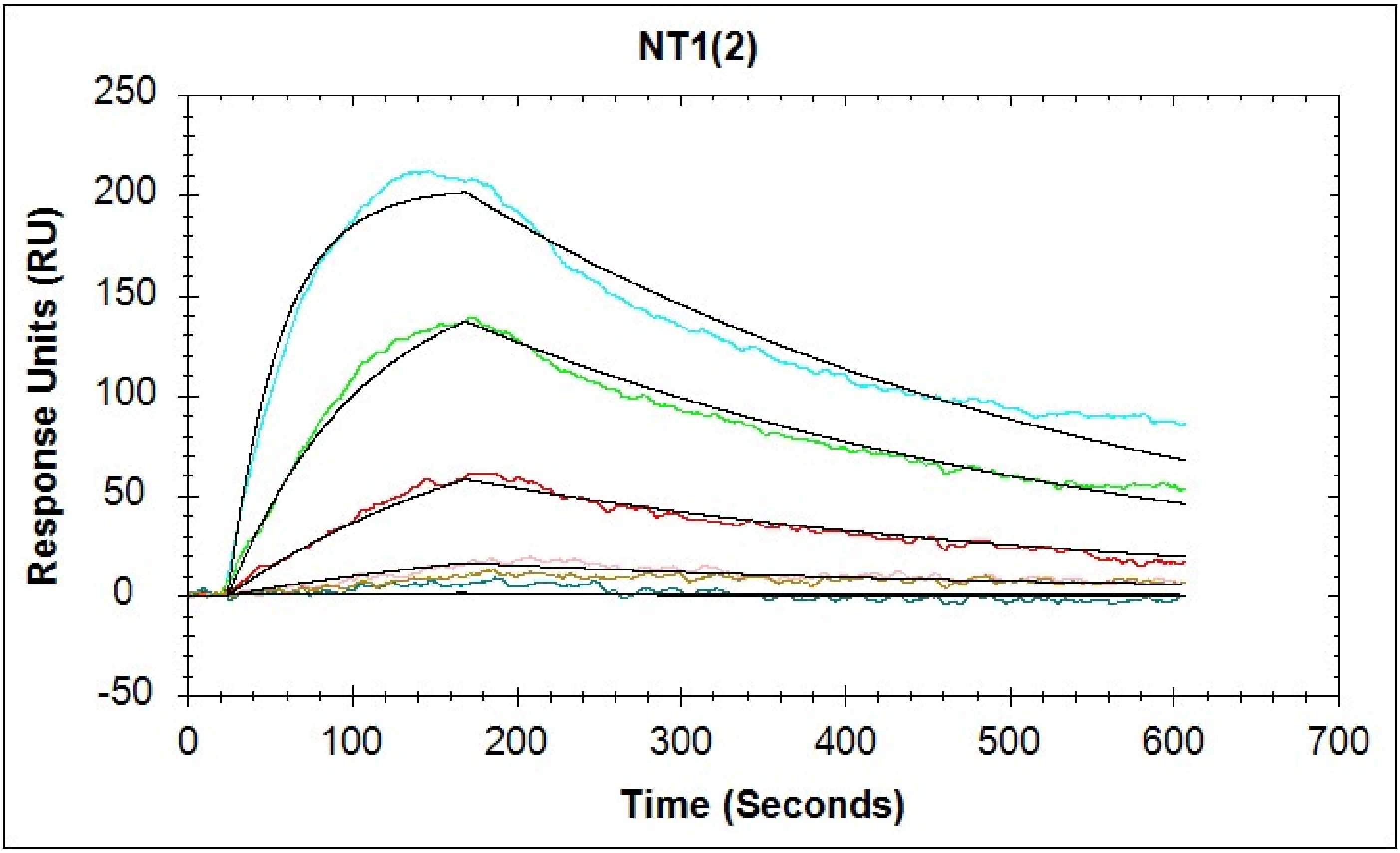

Evaluation type: OneToOne

Curve name	Bmax ([Response Units (RU)])	ka (1/(M*s))	kd (1/s)
KRAS 12 nM_8051.86s - Reference curve fitted	9.40 (±1.53e-1)	9.62e3 (±4.86e1)	2.50e-3 (±3.83e-7)
KRAS 37 nM_9041.9s - Reference curve fitted	23.38 (±1.39e-1)	9.62e3 (±4.86e1)	2.50e-3 (±3.83e-7)
KRAS 111 nM_10551.8s - Reference curve fitted	134.30 (±2.33e-2)	9.62e3 (±4.86e1)	2.50e-3 (±3.83e-7)
KRAS 333 nM_11395.84s - Reference curve fitted	183.68 (±2.47e-2)	9.62e3 (±4.86e1)	2.50e-3 (±3.83e-7)
KRAS 1 μM_12405.47s - Reference curve fitted	208.46 (±2.78e-3)	9.62e3 (±4.86e1)	2.50e-3 (±3.83e-7)
KRAS 3 μM_13407.7s - Reference curve fitted	221.15 (±1.93e-3)	9.62e3 (±4.86e1)	2.50e-3 (±3.83e-7)

Curve name	KD (M)	BI ([Response Units (RU)])	Chi2 ([Response Units (RU)]^2)
KRAS 12 nM_8051.86s - Reference curve fitted	2.60e-7 (±1.35e-9)	0.10	29.91
KRAS 37 nM_9041.9s - Reference curve fitted	2.60e-7 (±1.35e-9)	0.10	29.91
KRAS 111 nM_10551.8s - Reference curve fitted	2.60e-7 (±1.35e-9)	0.10	29.91
KRAS 333 nM_11395.84s - Reference curve fitted	2.60e-7 (±1.35e-9)	0.10	29.91
KRAS 1 μM_12405.47s - Reference curve fitted	2.60e-7 (±1.35e-9)	0.10	29.91
KRAS 3 μM_13407.7s - Reference curve fitted	2.60e-7 (±1.35e-9)	0.10	29.91

Curve name	U-value: ka/kd (%)
KRAS 12 nM_8051.86s - Reference curve fitted	1.60
KRAS 37 nM_9041.9s - Reference curve fitted	1.60
KRAS 111 nM_10551.8s - Reference curve fitted	1.60
KRAS 333 nM_11395.84s - Reference curve fitted	1.60
KRAS 1 μM_12405.47s - Reference curve fitted	1.60
KRAS 3 μM_13407.7s - Reference curve fitted	1.60

Run	Date	Source
NT1	-	New Overlay

	Curve	Ligand	Conc. (M)	Target	Source	Description
	KRAS 12 nM_8051.86s - Reference curve fitted		0		Kinetics evaluation.EvalItem(2)	Kinetic fit to curve KRAS 12 nM_8051.86s - Reference curve
	KRAS 37 nM_9041.9s - Reference curve fitted		0		Kinetics evaluation.EvalItem(2)	Kinetic fit to curve KRAS 37 nM_9041.9s - Reference curve
	KRAS 111 nM_10551.8s - Reference curve fitted		0		Kinetics evaluation.EvalItem(2)	Kinetic fit to curve KRAS 111 nM_10551.8s - Reference curve
	KRAS 333 nM_11395.84s - Reference curve fitted		0		Kinetics evaluation.EvalItem(2)	Kinetic fit to curve KRAS 333 nM_11395.84s - Reference curve
	KRAS 1 μM_12405.47s - Reference curve fitted		0		Kinetics evaluation.EvalItem(2)	Kinetic fit to curve KRAS 1 μM_12405.47s - Reference curve
	KRAS 3 μM_13407.7s - Reference curve fitted		0		Kinetics evaluation.EvalItem(2)	Kinetic fit to curve KRAS 3 μM_13407.7s - Reference curve
	KRAS 12 nM_8051.86s - Reference curve		1.20e-8, 0.00e0		New Overlay	
	KRAS 37 nM_9041.9s - Reference curve		3.70e-8, 0.00e0		New Overlay	
	KRAS 111 nM_10551.8s - Reference curve		1.11e-7, 0.00e0		New Overlay	
	KRAS 333 nM_11395.84s - Reference curve		3.33e-7, 0.00e0		New Overlay	
	KRAS 1 μM_12405.47s - Reference curve		1.00e-6, 0.00e0		New Overlay	
	KRAS 3 μM_13407.7s - Reference curve		3.00e-6, 0.00e0		New Overlay	
