# Supplementary material for: Unveiling the domain-specific and RAS isoform-specific details of BRAF kinase regulation
Source: eLife. 2023 Dec 27;12:RP88836. doi: 10.7554/eLife.88836 (PMC10752582; doi:10.7554/eLife.88836)
Supplement: Figure 4—source data 2. — Full test preview provided in .txt format for NT1, NT2, NT3, and NT4. NT2 data also applies for Figure 4—figure supplement 2. [file elife-88836-fig4-data2.zip › Figure 4- source data 2/NT2_GST-KRAS_3-23-23_fit.pdf]

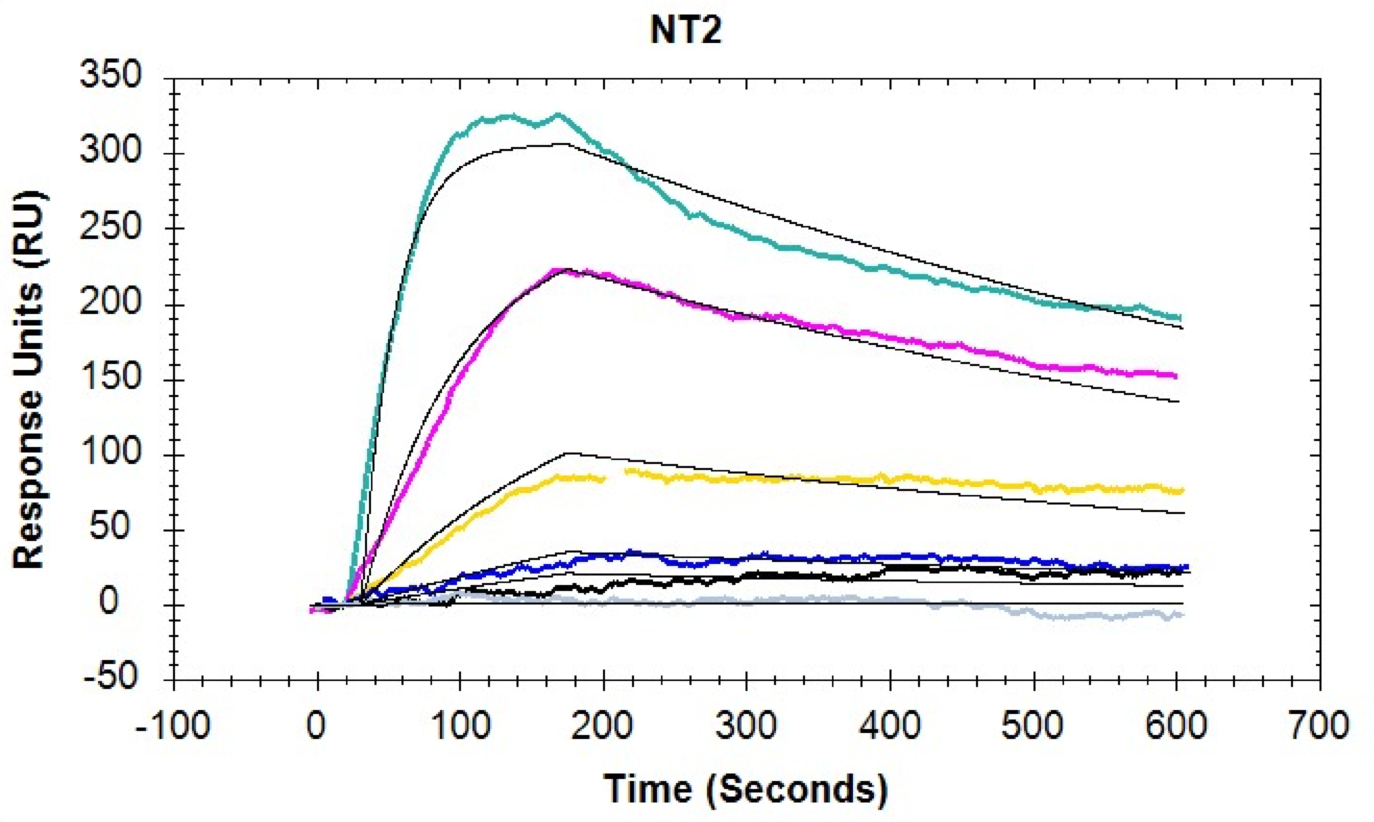

Description:
New Overlay(1)(6)

Evaluation type: OneToOne

Curve name	Bmax ([Signal (RU)])	ka (1/(M*s))	kd (1/s)	KD (M)
KRAS 4 nM_13132.64s - Reference curve fitted	32.03 (±5.99e1)	4.12e4 (±7.53e1)	1.19e-3 (±1.62e-6)	2.88e-8 (±9.20e-11)
KRAS 12 nM_14153.95s - Reference curve fitted	326.72 (±5.65e-2)	4.12e4 (±7.53e1)	1.19e-3 (±1.62e-6)	2.88e-8 (±9.20e-11)
KRAS 37 nM_16352.32s - Reference curve fitted	192.37 (±2.26e-2)	4.12e4 (±7.53e1)	1.19e-3 (±1.62e-6)	2.88e-8 (±9.20e-11)
KRAS 111 nM_17623.73s - Reference curve fitted	225.12 (±7.50e-3)	4.12e4 (±7.53e1)	1.19e-3 (±1.62e-6)	2.88e-8 (±9.20e-11)
KRAS 333 nM_18797.97s - Reference curve fitted	274.12 (±1.73e-3)	4.12e4 (±7.53e1)	1.19e-3 (±1.62e-6)	2.88e-8 (±9.20e-11)
KRAS 1 µM_20263.09s - Reference curve fitted	315.28 (±6.91e-4)	4.12e4 (±7.53e1)	1.19e-3 (±1.62e-6)	2.88e-8 (±9.20e-11)

Curve name	BI ([Signal (RU)])	Chi2 ([Signal (RU)]^2)	U-value: kd (%)
KRAS 4 nM_13132.64s - Reference curve fitted	0.10	70.18	2.20
KRAS 12 nM_14153.95s - Reference curve fitted	0.10	70.18	2.20
KRAS 37 nM_16352.32s - Reference curve fitted	0.10	70.18	2.20
KRAS 111 nM_17623.73s - Reference curve fitted	0.10	70.18	2.20
KRAS 333 nM_18797.97s - Reference curve fitted	0.10	70.18	2.20
KRAS 1 µM_20263.09s - Reference curve fitted	0.10	70.18	2.20

Run	Date	Source
New Overlay(1)	-	New Overlay(1)

	Curve	Ligand	Conc. (M)	Target	Source	Description
■	KRAS 4 nM_13132.64s - Reference curve fitted		0		Kinetics evaluation.EvalItem(6)	Kinetic fit to curve KRAS 4 nM_13132.64s - Reference curve
■	KRAS 12 nM_14153.95s - Reference curve fitted		0		Kinetics evaluation.EvalItem(6)	Kinetic fit to curve KRAS 12 nM_14153.95s - Reference curve
■	KRAS 37 nM_16352.32s - Reference curve fitted		0		Kinetics evaluation.EvalItem(6)	Kinetic fit to curve KRAS 37 nM_16352.32s - Reference curve
■	KRAS 111 nM_17623.73s - Reference curve fitted		0		Kinetics evaluation.EvalItem(6)	Kinetic fit to curve KRAS 111 nM_17623.73s - Reference curve
■	KRAS 333 nM_18797.97s - Reference curve fitted		0		Kinetics evaluation.EvalItem(6)	Kinetic fit to curve KRAS 333 nM_18797.97s - Reference curve
■	KRAS 1 µM_20263.09s - Reference curve fitted		0		Kinetics evaluation.EvalItem(6)	Kinetic fit to curve KRAS 1 µM_20263.09s - Reference curve
■	KRAS 4 nM_13132.64s - Reference curve		4.00e-9, 0.00e0		New Overlay(1)	
■	KRAS 12 nM_14153.95s - Reference curve		1.20e-8, 0.00e0		New Overlay(1)	
■	KRAS 37 nM_16352.32s - Reference curve		3.70e-8, 0.00e0		New Overlay(1)	
■	KRAS 111 nM_17623.73s - Reference curve		1.11e-7, 0.00e0		New Overlay(1)	
■	KRAS 333 nM_18797.97s - Reference curve		3.33e-7, 0.00e0		New Overlay(1)	
■	KRAS 1 µM_20263.09s - Reference curve		1.00e-6, 0.00e0		New Overlay(1)	
