# Supplementary material for: Unveiling the domain-specific and RAS isoform-specific details of BRAF kinase regulation
Source: eLife. 2023 Dec 27;12:RP88836. doi: 10.7554/eLife.88836 (PMC10752582; doi:10.7554/eLife.88836)
Supplement: Figure 4—source data 2. — Full test preview provided in .txt format for NT1, NT2, NT3, and NT4. NT2 data also applies for Figure 4—figure supplement 2. [file elife-88836-fig4-data2.zip › Figure 4- source data 2/NT3_GST-KRAS_8-17-23_fit.pdf]

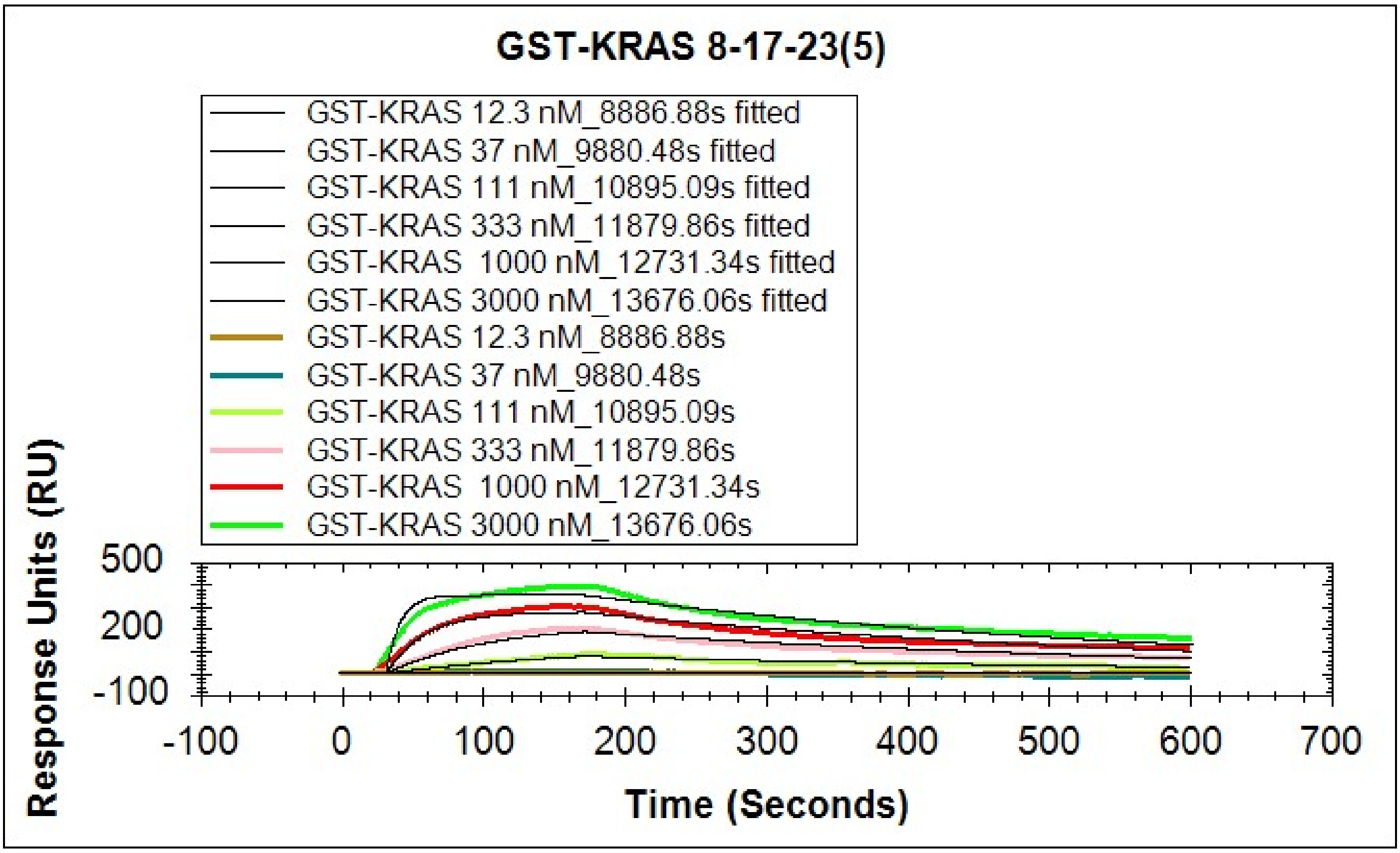

Description:
Bmax=local; BI=constant
no blank sub

Evaluation type: OneToOne

Curve name	Bmax ([Response Units (RU)])	ka (1/(M*s))	kd (1/s)	KD (M)
GST-KRAS 12.3 nM_8886.88s fitted	5.28 (±3.37e0)	3.42e4 (±1.15e2)	2.36e-3 (±9.75e-6)	6.90e-8 (±5.16e-10)
GST-KRAS 37 nM_9880.48s fitted	24.75 (±1.15e-1)	3.42e4 (±1.15e2)	2.36e-3 (±9.75e-6)	6.90e-8 (±5.16e-10)
GST-KRAS 111 nM_10895.09s fitted	215.43 (±1.84e-2)	3.42e4 (±1.15e2)	2.36e-3 (±9.75e-6)	6.90e-8 (±5.16e-10)
GST-KRAS 333 nM_11879.86s fitted	264.39 (±6.79e-3)	3.42e4 (±1.15e2)	2.36e-3 (±9.75e-6)	6.90e-8 (±5.16e-10)
GST-KRAS 1000 nM_12731.34s fitted	293.82 (±4.83e-3)	3.42e4 (±1.15e2)	2.36e-3 (±9.75e-6)	6.90e-8 (±5.16e-10)
GST-KRAS 3000 nM_13676.06s fitted	358.81 (±2.31e-3)	3.42e4 (±1.15e2)	2.36e-3 (±9.75e-6)	6.90e-8 (±5.16e-10)

Curve name	BI ([Response Units (RU)])	Chi2 ([Response Units (RU)]^2)	U-value: ka (%)
GST-KRAS 12.3 nM_8886.88s fitted	0.10	158.83	3.10
GST-KRAS 37 nM_9880.48s fitted	0.10	158.83	3.10
GST-KRAS 111 nM_10895.09s fitted	0.10	158.83	3.10
GST-KRAS 333 nM_11879.86s fitted	0.10	158.83	3.10
GST-KRAS 1000 nM_12731.34s fitted	0.10	158.83	3.10
GST-KRAS 3000 nM_13676.06s fitted	0.10	158.83	3.10

Run	Date	Source
GST-KRAS 8-17-23	-	New Overlay

	Curve	Ligand	Conc. (M)	Target	Source	Description
■	GST-KRAS 12.3 nM_8886.88s fitted		0		Kinetics evaluation.EvalItem(3)	Kinetic fit to curve GST-KRAS 12.3 nM_8886.88s
■	GST-KRAS 37 nM_9880.48s fitted		0		Kinetics evaluation.EvalItem(3)	Kinetic fit to curve GST-KRAS 37 nM_9880.48s
■	GST-KRAS 111 nM_10895.09s fitted		0		Kinetics evaluation.EvalItem(3)	Kinetic fit to curve GST-KRAS 111 nM_10895.09s
■	GST-KRAS 333 nM_11879.86s fitted		0		Kinetics evaluation.EvalItem(3)	Kinetic fit to curve GST-KRAS 333 nM_11879.86s
■	GST-KRAS 1000 nM_12731.34s fitted		0		Kinetics evaluation.EvalItem(3)	Kinetic fit to curve GST-KRAS 1000 nM_12731.34s
■	GST-KRAS 3000 nM_13676.06s fitted		0		Kinetics evaluation.EvalItem(3)	Kinetic fit to curve GST-KRAS 3000 nM_13676.06s
■	GST-KRAS 12.3 nM_8886.88s		1.23e-8, 0.00e0		New Overlay	
■	GST-KRAS 37 nM_9880.48s		3.70e-8, 0.00e0		New Overlay	
■	GST-KRAS 111 nM_10895.09s		1.11e-7, 0.00e0		New Overlay	
■	GST-KRAS 333 nM_11879.86s		3.33e-7, 0.00e0		New Overlay	
■	GST-KRAS 1000 nM_12731.34s		1.00e-6, 0.00e0		New Overlay	
■	GST-KRAS 3000 nM_13676.06s		3.00e-6, 0.00e0		New Overlay	
