# Supplementary material for: Unveiling the domain-specific and RAS isoform-specific details of BRAF kinase regulation
Source: eLife. 2023 Dec 27;12:RP88836. doi: 10.7554/eLife.88836 (PMC10752582; doi:10.7554/eLife.88836)
Supplement: Figure 4—source data 2. — Full test preview provided in .txt format for NT1, NT2, NT3, and NT4. NT2 data also applies for Figure 4—figure supplement 2. [file elife-88836-fig4-data2.zip › Figure 4- source data 2/NT4_GST-KRAS 8-3-23_fit.pdf]

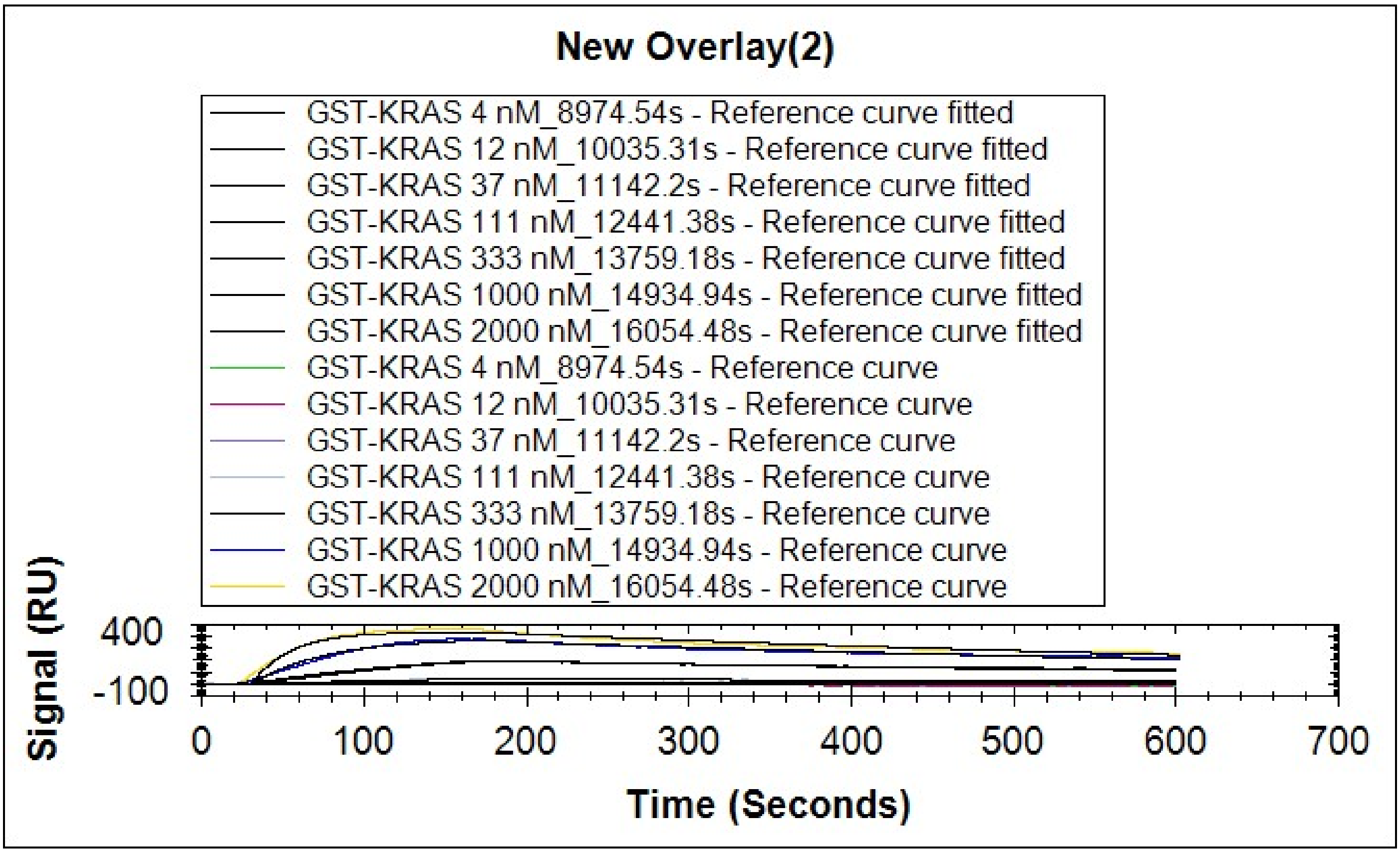

Evaluation type: OneToOne

Curve name	Bmax ([Signal (RU)])	ka (1/(M*s))	kd (1/s)
GST-KRAS 4 nM_8974.54s - Reference curve fitted	1.53 (±3.99e-1)	2.01e4 (±5.74e1)	1.37e-3 (±5.11e-7)
GST-KRAS 12 nM_10035.31s - Reference curve fitted	0.00 (±3.26e4)	2.01e4 (±5.74e1)	1.37e-3 (±5.11e-7)
GST-KRAS 37 nM_11142.2s - Reference curve fitted	11.17 (±3.26e-3)	2.01e4 (±5.74e1)	1.37e-3 (±5.11e-7)
GST-KRAS 111 nM_12441.38s - Reference curve fitted	160.63 (±7.69e-3)	2.01e4 (±5.74e1)	1.37e-3 (±5.11e-7)
GST-KRAS 333 nM_13759.18s - Reference curve fitted	337.55 (±3.30e-4)	2.01e4 (±5.74e1)	1.37e-3 (±5.11e-7)
GST-KRAS 1000 nM_14934.94s - Reference curve fitted	402.46 (±5.33e-5)	2.01e4 (±5.74e1)	1.37e-3 (±5.11e-7)
GST-KRAS 2000 nM_16054.48s - Reference curve fitted	442.89 (±9.60e-5)	2.01e4 (±5.74e1)	1.37e-3 (±5.11e-7)

Curve name	KD (M)	BI ([Signal (RU)])	Chi2 ([Signal (RU)]^2)
GST-KRAS 4 nM_8974.54s - Reference curve fitted	6.81e-8 (±2.20e-10)	0.10	121.02
GST-KRAS 12 nM_10035.31s - Reference curve fitted	6.81e-8 (±2.20e-10)	0.10	121.02
GST-KRAS 37 nM_11142.2s - Reference curve fitted	6.81e-8 (±2.20e-10)	0.10	121.02
GST-KRAS 111 nM_12441.38s - Reference curve fitted	6.81e-8 (±2.20e-10)	0.10	121.02
GST-KRAS 333 nM_13759.18s - Reference curve fitted	6.81e-8 (±2.20e-10)	0.10	121.02
GST-KRAS 1000 nM_14934.94s - Reference curve fitted	6.81e-8 (±2.20e-10)	0.10	121.02
GST-KRAS 2000 nM_16054.48s - Reference curve fitted	6.81e-8 (±2.20e-10)	0.10	121.02

Curve name	U-value: ka/kd (%)
GST-KRAS 4 nM_8974.54s - Reference curve fitted	1.70
GST-KRAS 12 nM_10035.31s - Reference curve fitted	1.70
GST-KRAS 37 nM_11142.2s - Reference curve fitted	1.70
GST-KRAS 111 nM_12441.38s - Reference curve fitted	1.70
GST-KRAS 333 nM_13759.18s - Reference curve fitted	1.70
GST-KRAS 1000 nM_14934.94s - Reference curve fitted	1.70
GST-KRAS 2000 nM_16054.48s - Reference curve fitted	1.70

Run	Date	Source
New Overlay	-	New Overlay

	Curve	Ligand	Conc. (M)	Target	Source	Description
■	GST-KRAS 4 nM_8974.54s - Reference curve fitted		0		Kinetics evaluation.EvalItem	Kinetic fit to curve GST-KRAS 4 nM_8974.54s - Reference curve
■	GST-KRAS 12 nM_10035.31s - Reference curve fitted		0		Kinetics evaluation.EvalItem	Kinetic fit to curve GST-KRAS 12 nM_10035.31s - Reference curve
■	GST-KRAS 37 nM_11142.2s - Reference curve fitted		0		Kinetics evaluation.EvalItem	Kinetic fit to curve GST-KRAS 37 nM_11142.2s - Reference curve
■	GST-KRAS 111 nM_12441.38s - Reference curve fitted		0		Kinetics evaluation.EvalItem	Kinetic fit to curve GST-KRAS 111 nM_12441.38s - Reference curve
■	GST-KRAS 333 nM_13759.18s - Reference curve fitted		0		Kinetics evaluation.EvalItem	Kinetic fit to curve GST-KRAS 333 nM_13759.18s - Reference curve
■	GST-KRAS 1000 nM_14934.94s - Reference curve fitted		0		Kinetics evaluation.EvalItem	Kinetic fit to curve GST-KRAS 1000 nM_14934.94s - Reference curve
■	GST-KRAS 2000 nM_16054.48s - Reference curve fitted		0		Kinetics evaluation.EvalItem	Kinetic fit to curve GST-KRAS 2000 nM_16054.48s - Reference curve
■	GST-KRAS 4 nM_8974.54s - Reference curve		4.00e-9, 0.00e0		New Overlay	
■	GST-KRAS 12 nM_10035.31s - Reference curve		1.20e-8, 0.00e0		New Overlay	
■	GST-KRAS 37 nM_11142.2s - Reference curve		3.70e-8, 0.00e0		New Overlay	
■	GST-KRAS 111 nM_12441.38s - Reference curve		1.11e-7, 0.00e0		New Overlay	
■	GST-KRAS 333 nM_13759.18s - Reference curve		3.33e-7, 0.00e0		New Overlay	
■	GST-KRAS 1000 nM_14934.94s - Reference curve		1.00e-6, 0.00e0		New Overlay	
■	GST-KRAS 2000 nM_16054.48s - Reference curve		2.00e-6, 0.00e0		New Overlay	
