# Supplementary material for: Unveiling the domain-specific and RAS isoform-specific details of BRAF kinase regulation
Source: eLife. 2023 Dec 27;12:RP88836. doi: 10.7554/eLife.88836 (PMC10752582; doi:10.7554/eLife.88836)
Supplement: Figure 4—source data 3. — Full test preview provided in .txt format for FL-BRAF. Data applies for Figure 4—figure supplement 4b. [file elife-88836-fig4-data3.zip › Figure 4- source data 3/FL-BRAF_GST-KRAS_8-11-23_fit.pdf]

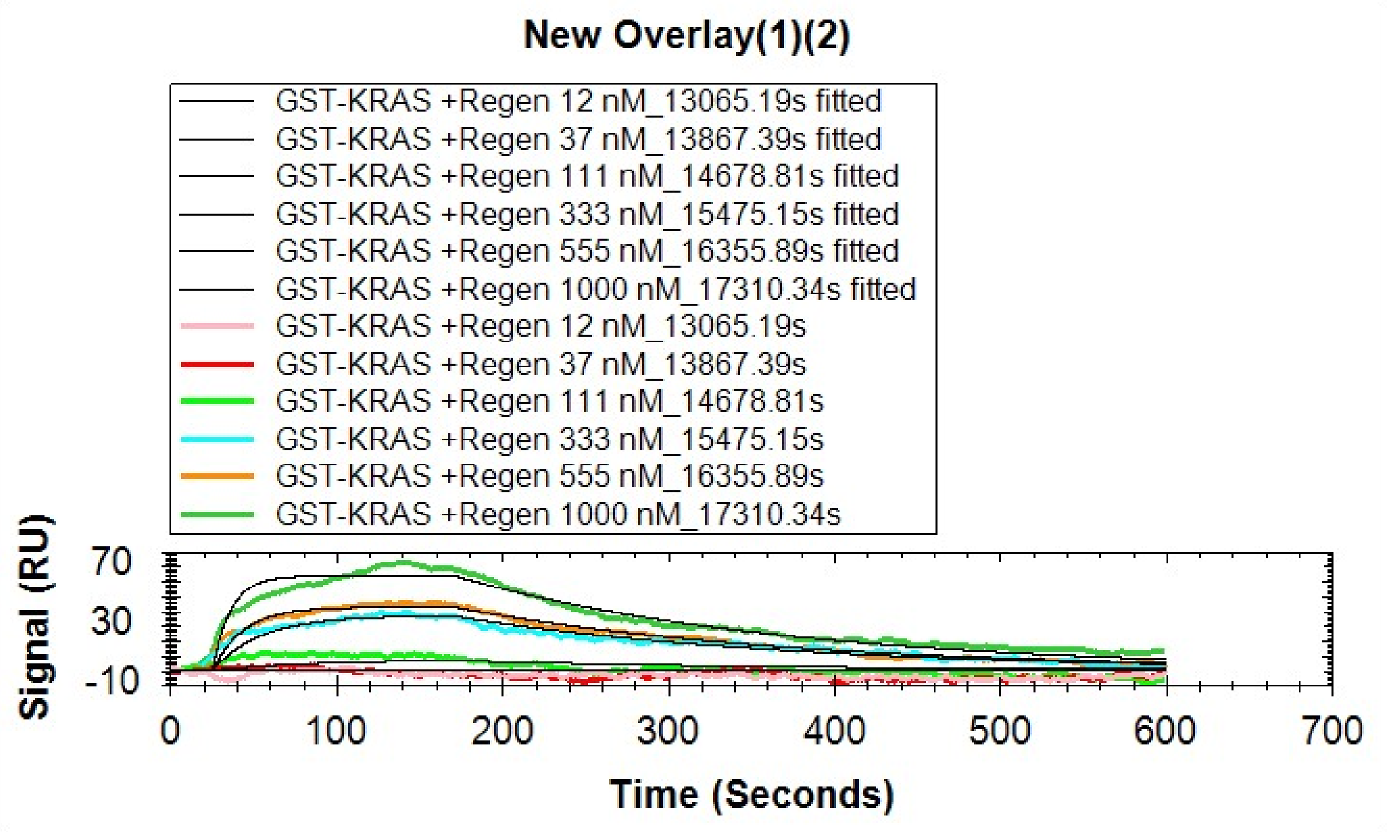

Evaluation type: OneToOne

Curve name	Bmax ([Signal (RU)])	ka (1/(M*s))	kd (1/s)	KD (M)
GST-KRAS +Regen 12 nM_13065.19s fitted	2.05 (±2.02e-2)	8.75e4 (±1.31e3)	5.00e-3 (±1.66e-6)	5.72e-8 (±8.74e-10)
GST-KRAS +Regen 37 nM_13867.39s fitted	1.22 (±1.23e-2)	8.75e4 (±1.31e3)	5.00e-3 (±1.66e-6)	5.72e-8 (±8.74e-10)
GST-KRAS +Regen 111 nM_14678.81s fitted	11.19 (±3.21e-2)	8.75e4 (±1.31e3)	5.00e-3 (±1.66e-6)	5.72e-8 (±8.74e-10)
GST-KRAS +Regen 333 nM_15475.15s fitted	42.89 (±3.50e-3)	8.75e4 (±1.31e3)	5.00e-3 (±1.66e-6)	5.72e-8 (±8.74e-10)
GST-KRAS +Regen 555 nM_16355.89s fitted	46.85 (±3.07e-3)	8.75e4 (±1.31e3)	5.00e-3 (±1.66e-6)	5.72e-8 (±8.74e-10)
GST-KRAS +Regen 1000 nM_17310.34s fitted	66.89 (±1.31e-3)	8.75e4 (±1.31e3)	5.00e-3 (±1.66e-6)	5.72e-8 (±8.74e-10)

Curve name	BI ([Signal (RU)])	Chi2 ([Signal (RU)]^2)	U-value: ka (%)
GST-KRAS +Regen 12 nM_13065.19s fitted	0.10	14.03	8.30
GST-KRAS +Regen 37 nM_13867.39s fitted	0.10	14.03	8.30
GST-KRAS +Regen 111 nM_14678.81s fitted	0.10	14.03	8.30
GST-KRAS +Regen 333 nM_15475.15s fitted	0.10	14.03	8.30
GST-KRAS +Regen 555 nM_16355.89s fitted	0.10	14.03	8.30
GST-KRAS +Regen 1000 nM_17310.34s fitted	0.10	14.03	8.30

Run	Date	Source
New Overlay(1)	-	New Overlay(1)

	Curve	Ligand	Conc. (M)	Target	Source	Description
■	GST-KRAS +Regen 12 nM_13065.19s fitted		0		Kinetics evaluation.EvalItem(1)	Kinetic fit to curve GST-KRAS +Regen 12 nM_13065.19s
■	GST-KRAS +Regen 37 nM_13867.39s fitted		0		Kinetics evaluation.EvalItem(1)	Kinetic fit to curve GST-KRAS +Regen 37 nM_13867.39s
■	GST-KRAS +Regen 111 nM_14678.81s fitted		0		Kinetics evaluation.EvalItem(1)	Kinetic fit to curve GST-KRAS +Regen 111 nM_14678.81s
■	GST-KRAS +Regen 333 nM_15475.15s fitted		0		Kinetics evaluation.EvalItem(1)	Kinetic fit to curve GST-KRAS +Regen 333 nM_15475.15s
■	GST-KRAS +Regen 555 nM_16355.89s fitted		0		Kinetics evaluation.EvalItem(1)	Kinetic fit to curve GST-KRAS +Regen 555 nM_16355.89s
■	GST-KRAS +Regen 1000 nM_17310.34s fitted		0		Kinetics evaluation.EvalItem(1)	Kinetic fit to curve GST-KRAS +Regen 1000 nM_17310.34s
■	GST-KRAS +Regen 12 nM_13065.19s		1.20e-8, 0.00e0		New Overlay(1)	
■	GST-KRAS +Regen 37 nM_13867.39s		3.70e-8, 0.00e0		New Overlay(1)	
■	GST-KRAS +Regen 111 nM_14678.81s		1.11e-7, 0.00e0		New Overlay(1)	
■	GST-KRAS +Regen 333 nM_15475.15s		3.33e-7, 0.00e0		New Overlay(1)	
■	GST-KRAS +Regen 555 nM_16355.89s		5.55e-7, 0.00e0		New Overlay(1)	
■	GST-KRAS +Regen 1000 nM_17310.34s		1.00e-6, 0.00e0		New Overlay(1)	
