# Supplementary material for: Unveiling the domain-specific and RAS isoform-specific details of BRAF kinase regulation
Source: eLife. 2023 Dec 27;12:RP88836. doi: 10.7554/eLife.88836 (PMC10752582; doi:10.7554/eLife.88836)
Supplement: Figure 4—figure supplement 3—source data 2. — Full test preview provided in .txt format for NT2. [file elife-88836-fig4-figsupp3-data2.zip › Figure 4- figure supplement 3- source data 2/NT2_KRAS2_2-17-23_fit.pdf]

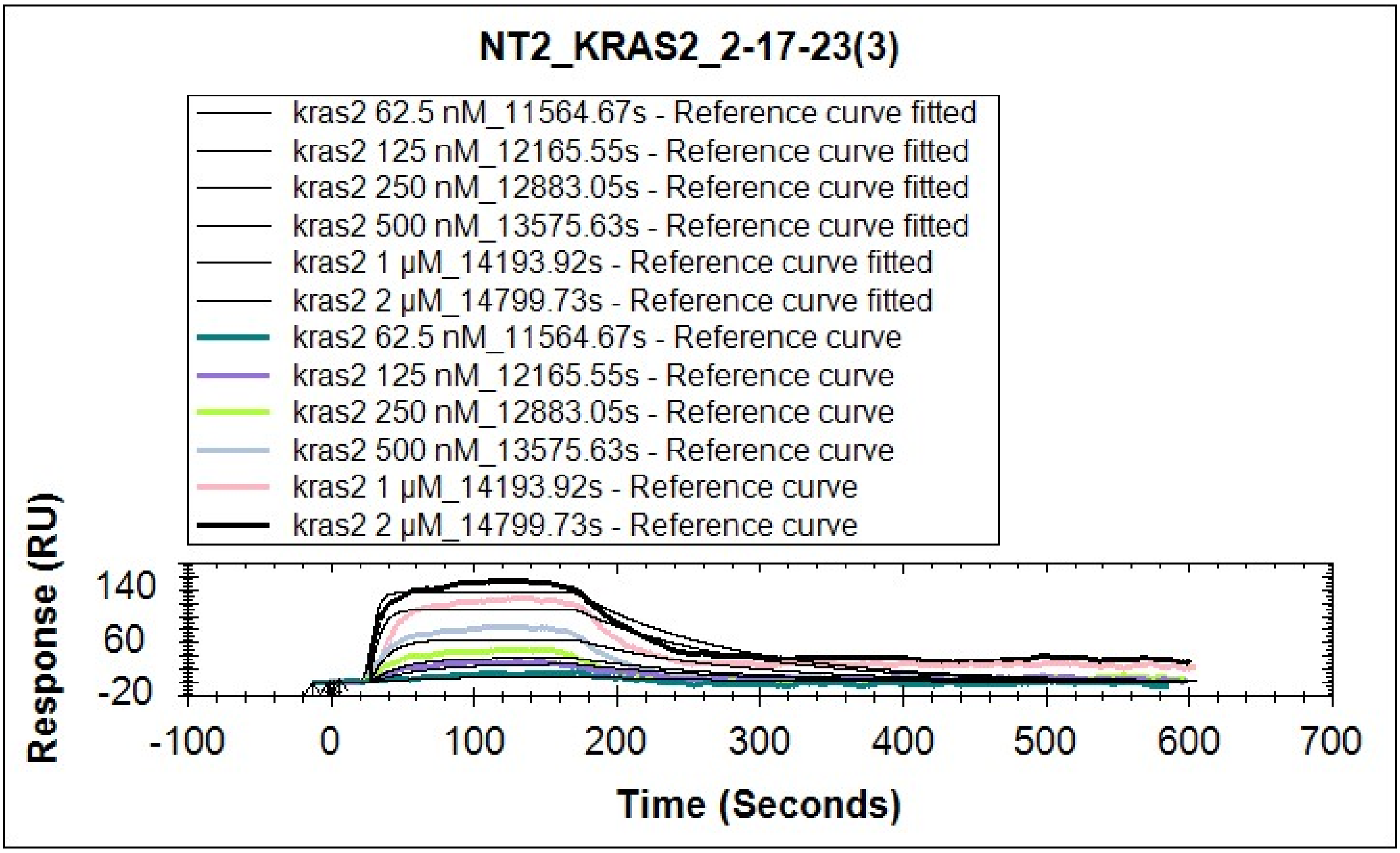

Evaluation type: OneToOne

Curve name	Bmax ([Response (RU)])	ka (1/(M*s))	kd (1/s)	KD (M)
kras2 62.5 nM_11564.67s - Reference curve fitted	19.81 (±4.33e-1)	1.41e5 (±3.08e3)	9.07e-3 (±1.32e-5)	6.42e-8 (±1.49e-9)
kras2 125 nM_12165.55s - Reference curve fitted	41.88 (±1.13e0)	1.41e5 (±3.08e3)	9.07e-3 (±1.32e-5)	6.42e-8 (±1.49e-9)
kras2 250 nM_12883.05s - Reference curve fitted	45.42 (±1.85e0)	1.41e5 (±3.08e3)	9.07e-3 (±1.32e-5)	6.42e-8 (±1.49e-9)
kras2 500 nM_13575.63s - Reference curve fitted	71.92 (±3.10e-1)	1.41e5 (±3.08e3)	9.07e-3 (±1.32e-5)	6.42e-8 (±1.49e-9)
kras2 1 µM_14193.92s - Reference curve fitted	116.28 (±4.83e-2)	1.41e5 (±3.08e3)	9.07e-3 (±1.32e-5)	6.42e-8 (±1.49e-9)
kras2 2 µM_14799.73s - Reference curve fitted	138.14 (±6.60e-2)	1.41e5 (±3.08e3)	9.07e-3 (±1.32e-5)	6.42e-8 (±1.49e-9)

Curve name	BI ([Response (RU)])	Chi2 ([Response (RU)]^2)	U-value: ka (%)
kras2 62.5 nM_11564.67s - Reference curve fitted	0.10	138.49	16.70
kras2 125 nM_12165.55s - Reference curve fitted	0.10	138.49	16.70
kras2 250 nM_12883.05s - Reference curve fitted	0.10	138.49	16.70
kras2 500 nM_13575.63s - Reference curve fitted	0.10	138.49	16.70
kras2 1 µM_14193.92s - Reference curve fitted	0.10	138.49	16.70
kras2 2 µM_14799.73s - Reference curve fitted	0.10	138.49	16.70

Run	Date	Source
NT2_KRAS2_2-17-23	-	New Overlay

	Curve	Ligand	Conc. (M)	Target	Source	Description
■	kras2 62.5 nM_11564.67s - Reference curve fitted		0		Kinetics evaluation.EvalItem(1)	Kinetic fit to curve kras2 62.5 nM_11564.67s - Reference curve
■	kras2 125 nM_12165.55s - Reference curve fitted		0		Kinetics evaluation.EvalItem(1)	Kinetic fit to curve kras2 125 nM_12165.55s - Reference curve
■	kras2 250 nM_12883.05s - Reference curve fitted		0		Kinetics evaluation.EvalItem(1)	Kinetic fit to curve kras2 250 nM_12883.05s - Reference curve
■	kras2 500 nM_13575.63s - Reference curve fitted		0		Kinetics evaluation.EvalItem(1)	Kinetic fit to curve kras2 500 nM_13575.63s - Reference curve
■	kras2 1 µM_14193.92s - Reference curve fitted		0		Kinetics evaluation.EvalItem(1)	Kinetic fit to curve kras2 1 µM_14193.92s - Reference curve
■	kras2 2 µM_14799.73s - Reference curve fitted		0		Kinetics evaluation.EvalItem(1)	Kinetic fit to curve kras2 2 µM_14799.73s - Reference curve
■	kras2 62.5 nM_11564.67s - Reference curve		6.25e-8, 0.00e0		New Overlay	
■	kras2 125 nM_12165.55s - Reference curve		1.25e-7, 0.00e0		New Overlay	
■	kras2 250 nM_12883.05s - Reference curve		2.50e-7, 0.00e0		New Overlay	
■	kras2 500 nM_13575.63s - Reference curve		5.00e-7, 0.00e0		New Overlay	
■	kras2 1 µM_14193.92s - Reference curve		1.00e-6, 0.00e0		New Overlay	
■	kras2 2 µM_14799.73s - Reference curve		2.00e-6, 0.00e0		New Overlay	
