# Supplementary material for: Unveiling the domain-specific and RAS isoform-specific details of BRAF kinase regulation
Source: eLife. 2023 Dec 27;12:RP88836. doi: 10.7554/eLife.88836 (PMC10752582; doi:10.7554/eLife.88836)
Supplement: Figure 5—source data 2. — Full test preview provided in .txt format for NT1 and NT3. Data for NT1 applies to curves in Figure 6B and C. [file elife-88836-fig5-data2.zip › Figure 5- source data 2/NT1_KD_3-25-2021_fit.pdf]

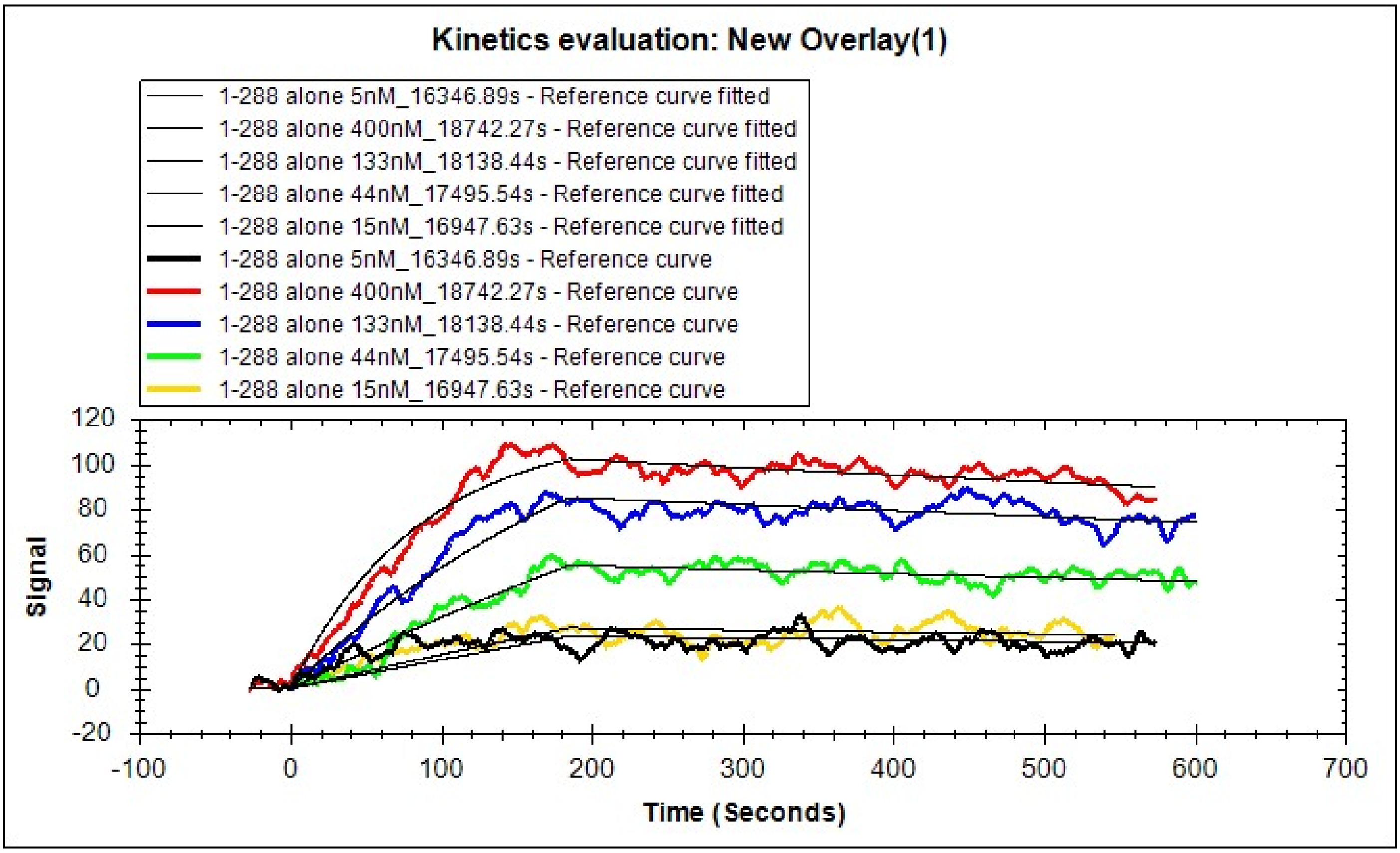

Evaluation type: OneToOne

Curve name	Bmax (Signal)	ka (1/(M*s))	kd (1/s)	KD (M)
1-288 alone 15nM_16947.63s - Reference curve fitted	372.33 (±5.23e-1)	2.81e4 (±3.71e1)	3.35e-4 (±6.77e-6)	1.19e-8 (±2.56e-10)
1-288 alone 133nM_18138.44s - Reference curve fitted	175.34 (±1.50e-2)	2.81e4 (±3.71e1)	3.35e-4 (±6.77e-6)	1.19e-8 (±2.56e-10)
1-288 alone 44nM_17495.54s - Reference curve fitted	276.89 (±6.85e-2)	2.81e4 (±3.71e1)	3.35e-4 (±6.77e-6)	1.19e-8 (±2.56e-10)
1-288 alone 5nM_16346.89s - Reference curve fitted	936.55 (±7.01e0)	2.81e4 (±3.71e1)	3.35e-4 (±6.77e-6)	1.19e-8 (±2.56e-10)
1-288 alone 400nM_18742.27s - Reference curve fitted	119.45 (±1.09e-2)	2.81e4 (±3.71e1)	3.35e-4 (±6.77e-6)	1.19e-8 (±2.56e-10)

Curve name	BI (Signal)	Chi2 (Signal^2)	U-value: kd (%)
1-288 alone 15nM_16947.63s - Reference curve fitted	0.00	23.37	7.50
1-288 alone 133nM_18138.44s - Reference curve fitted	0.00	23.37	7.50
1-288 alone 44nM_17495.54s - Reference curve fitted	0.00	23.37	7.50
1-288 alone 5nM_16346.89s - Reference curve fitted	0.00	23.37	7.50
1-288 alone 400nM_18742.27s - Reference curve fitted	0.00	23.37	7.50

Run	Source
New Overlay(1)	New Overlay(1)
