# Supplementary material for: Unveiling the domain-specific and RAS isoform-specific details of BRAF kinase regulation
Source: eLife. 2023 Dec 27;12:RP88836. doi: 10.7554/eLife.88836 (PMC10752582; doi:10.7554/eLife.88836)
Supplement: Figure 5—source data 2. — Full test preview provided in .txt format for NT1 and NT3. Data for NT1 applies to curves in Figure 6B and C. [file elife-88836-fig5-data2.zip › Figure 5- source data 2/NT3_KD_11-19-21_fit.pdf]

New Overlay(1)(2)

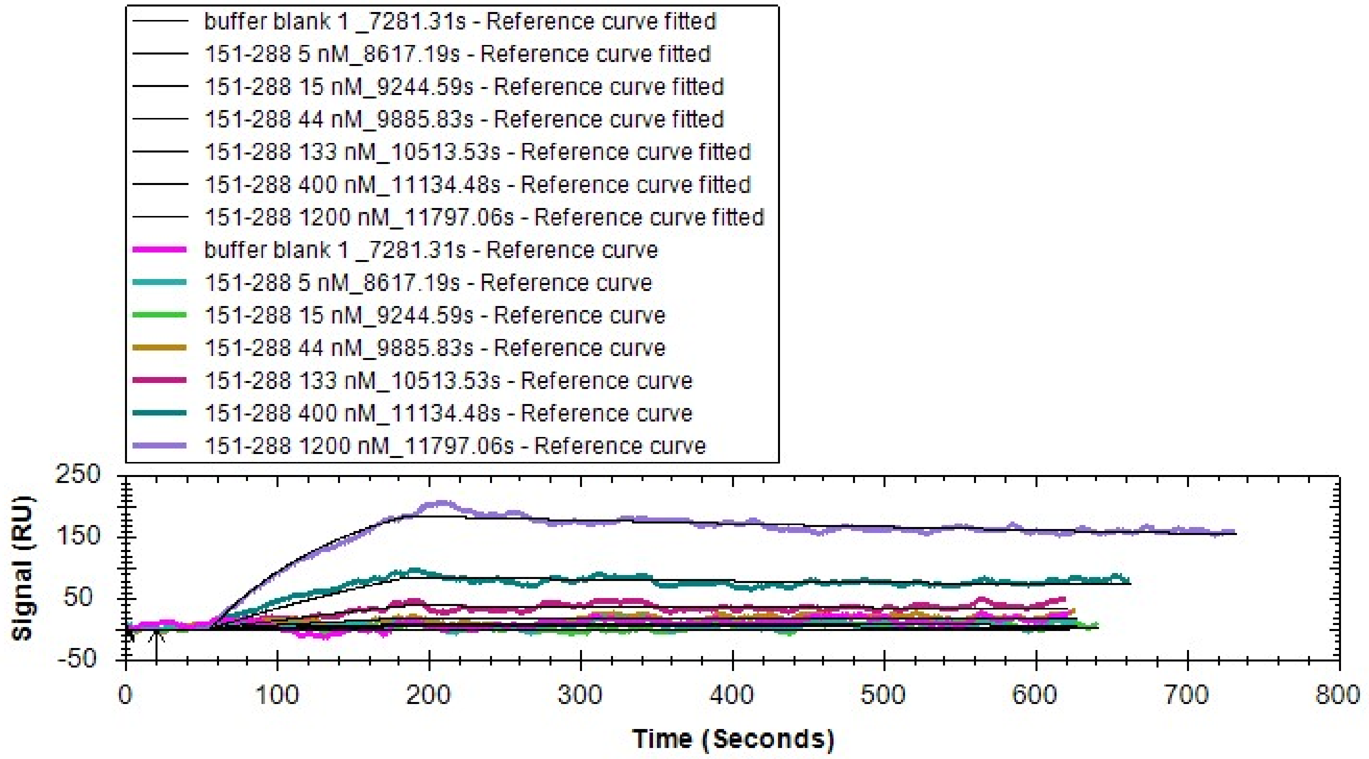

Evaluation type: OneToOne

Curve name	Bmax ([Signal (RU)])	ka (1/(M*s))	kd (1/s)	KD (M)
buffer blank 1 _7281.31s - Reference curve fitted	127901.14 (±4.58e4)	8.67e3 (±4.22e1)	3.19e-4 (±1.21e-5)	3.69e-8 (±1.57e-9)
151-288 5 nM_8617.19s - Reference curve fitted	1335.12 (±4.04e-1)	8.67e3 (±4.22e1)	3.19e-4 (±1.21e-5)	3.69e-8 (±1.57e-9)
151-288 15 nM_9244.59s - Reference curve fitted	225.95 (±5.27e-1)	8.67e3 (±4.22e1)	3.19e-4 (±1.21e-5)	3.69e-8 (±1.57e-9)
151-288 44 nM_9885.83s - Reference curve fitted	386.46 (±3.13e-1)	8.67e3 (±4.22e1)	3.19e-4 (±1.21e-5)	3.69e-8 (±1.57e-9)
151-288 133 nM_10513.53s - Reference curve fitted	280.22 (±2.33e-1)	8.67e3 (±4.22e1)	3.19e-4 (±1.21e-5)	3.69e-8 (±1.57e-9)
151-288 400 nM_11134.48s - Reference curve fitted	236.24 (±2.59e-2)	8.67e3 (±4.22e1)	3.19e-4 (±1.21e-5)	3.69e-8 (±1.57e-9)
151-288 1200 nM_11797.06s - Reference curve fitted	252.49 (±4.31e-3)	8.67e3 (±4.22e1)	3.19e-4 (±1.21e-5)	3.69e-8 (±1.57e-9)

Curve name	BI ([Signal (RU)])	Chi2 ([Signal (RU)]^2)	U-value: kd (%)
buffer blank 1 _7281.31s - Reference curve fitted	0.10	54.00	7.10
151-288 5 nM_8617.19s - Reference curve fitted	0.10	54.00	7.10
151-288 15 nM_9244.59s - Reference curve fitted	0.10	54.00	7.10
151-288 44 nM_9885.83s - Reference curve fitted	0.10	54.00	7.10
151-288 133 nM_10513.53s - Reference curve fitted	0.10	54.00	7.10
151-288 400 nM_11134.48s - Reference curve fitted	0.10	54.00	7.10
151-288 1200 nM_11797.06s - Reference curve fitted	0.10	54.00	7.10

Run	Date	Source
New Overlay(1)	-	New Overlay(1)
